# Do Robotic Tutors Compromise the Social-Emotional Development of Children?

**DOI:** 10.3389/frobt.2022.734955

**Published:** 2022-01-21

**Authors:** Matthijs H. J. Smakman, Elly A. Konijn, Paul A. Vogt

**Affiliations:** ^1^ Department of Communication Science, VU University Amsterdam, Amsterdam, Netherlands; ^2^ Institute for Information and Communication Technology, HU University of Applied Sciences Utrecht, Utrecht, Netherlands; ^3^ School of Communication, Media and IT, Research Group Digital Transformation, Hanze University of Applied Sciences Groningen, Groningen, Netherlands

**Keywords:** social robots, child-robot interaction, education, social development, primary school, social skills, bonding, friendship

## Abstract

Social robots are reported to hold great potential for education. However, both scholars and key stakeholders worry about children’s social-emotional development being compromised. In aiming to provide new insights into the impact that social robots can have on the social-emotional development of children, the current study interviewed teachers who use social robots in their day-to-day educational practice. The results of our interviews with these experienced teachers indicate that the social robots currently used in education pose little threat to the social-emotional development of children. Children with special needs seem to be more sensitive to social-affective bonding with a robot compared to regular children. This bond seems to have positive effects in enabling them to more easily connect with their human peers and teachers. However, when robots are being introduced more regularly, daily, without the involvement of a human teacher, new issues could arise. For now, given the current state of technology and the way social robots are being applied, other (ethical) issues seem to be more urgent, such as privacy, security and the workload of teachers. Future studies should focus on these issues first, to ensure a safe and effective educational environment for both children and teachers.

## Introduction

Social robots are gradually being introduced in primary education. They provide new opportunities for improving cognitive outcomes, such as learning a second language (Vogt et al., 2019; Konijn et al., 2021), rehearsing the times tables (Konijn and Hoorn, 2020), learning sign language (Luccio and Gaspari, 2020) and training handwriting (Aktar Mispa and Sojib, 2020). In addition, social robots are used to support motivational and affective elements of learning (e.g., the learner being attentive, receptive, responsive, reflective, or inquisitive) (Belpaeme et al., 2018). Although social robots show potential as learning or teaching companions for children, according to a recent literature review (Johal, 2020), other studies on the use of social robots in education have reported that it is too early to conclude that robots are, for instance, effective as language tutors (van den Berghe et al., 2019), or more effective than human teachers or other types of technology (Woo et al., 2021). Furthermore, both scholars (Sharkey, 2016; Woo et al., 2021) and stakeholders (Smakman et al., 2021a) have voiced concerns related to social robots potentially harming children’s social-emotional development.

*Social* robots differ from other types of robots used in education, such as STEM robots. Other than STEM robots, social robots are designed to take on social roles such as that of a tutor or peer that assists children during their learning process. Having physical embodiment, the option to act (semi-) autonomously, and the capability to interact with humans by following social norms, can be considered as the three defining capacities for social robots (Hegel et al., 2009). Using these capacities, a robot can act as a social entity, such as in the role of a tutor, a peer, or that of a naïve learner (Hood et al., 2015). The feeling that users are socially connected with robots is central to the field of social robotics (Belpaeme et al., 2013).

Children’s social-emotional development is not only important during childhood, but also for adulthood and public health, because it is associated with academic performance, substance abuse, mental health, workplace and academic performance (Cherniss, 2000; Denham, 2006; Tremblay, 2020). Children’s social-emotional development can be characterized by five domains: 1) social competence, 2) attachment, 3) emotional competence, 4) self-perceived competence, and 5) temperament/personality (Denham et al., 2009). Milestones in social-emotional development domains differ per developmental period of children. For the purpose of this study, we will focus on the milestones associated with the primary school period. The first domain, social competence, can be defined as a child’s ability and effectiveness in social interaction (Rose-Krasnor, 1997). Children’s general developmental tasks related to social competence that should be assessed in primary school are the formation of dyadic friendships, solidification of peer status, and general diminution of physical aggression. Related to attachment, children in primary school should begin to balance the connection to parents and peers. The milestones for children in primary school related to emotional competence are the ability to understand complex emotions, such as unique perspective and ambivalence, and to be able to apply cognitive strategies to regulate emotions. Children’s self-perception of competence can be defined as “*one’s evaluations of one’s own abilities, including the child’s own assessment of his/her cognitive, physical and social abilities, especially in comparison with those of others”* (Denham et al., 2009, p. 44). During primary school, children’s views of their own competence become more complex, earlier notions of self-perceived competence are solidified and social evaluations by peers and teachers become more important (Denham et al., 2009). Lastly, for the domain temperament/personality, children’s personality attributes become increasingly differentiated during primary school. In earlier research, social robots have been reported to potentially influence several aspects of the social-emotional development domains, such as social competence (Peter et al., 2021) and attachment (Coeckelbergh et al., 2016).

Key stakeholders, such as teachers, parents, and policymakers, have also voiced concerns related to the potential social-affective bond that children may develop with a robot (Serholt et al., 2017; Smakman et al., 2020a, Smakman et al., 2020b). They report worries in the field that such a bond could harm children’s social-emotional development (Smakman et al., 2021b). Children bonding with robots could lead to children preferring the interaction with robots over that of their human friends and teachers, potentially resulting in the loss of human contact (Sharkey, 2016; Pandey and Gelin, 2017), social isolation (Kennedy et al., 2016), and dehumanization (Serholt et al., 2017). Children could also start to expect too much from robots, which could lead to children ending up feeling deceived or feeling anxious when the robot is absent (Sharkey, 2016). These potential risks related to the social-affective bond that children may develop with a robot might harm the children’s social-emotional development. According to a recent study (Pashevich, 2021), it is still unclear what kind of effect social robots might have on the social-emotional development of children.

Children have been reported to perceive social robots as entities with whom they will likely form social relationships (van Straten et al., 2020). What kind of relationships children form with robots is still unclear. For example, children are reported to perceive social robots as potential private tutors (Shin and Kim, 2007), possible rivals (Shin and Kim, 2007), and even friends (Lin et al., 2009). Various scholars argue that this newly perceived bond with technology might influence children’s behavior, both positively and negatively. Researchers have found that robots seem able to elicit socially desirable behavior among children, such as sharing, but they may also elicit socially undesirable behavior, such as aggressive behavior (Peter et al., 2021). Children have also been recorded to express bullying behavior towards an educational robot (Kanda et al., 2012) and others have expressed concerns related to the robot becoming a bully or becoming subject to bullying (Diep et al., 2015). What type of children are more susceptible to the influence of the robot on social-emotional domains, however, is still unclear. According to a recent study (Tolksdorf et al., 2021), the influence of individual variables, such as shyness, are still understudied in the field of child-robot interaction (CRI).

Measuring social-emotional development is complex. For each domain of children’s social-emotional development, there exist multiple measurement instruments such as the *Rothbart Child Behavior Questionnaire* for emotional competence (Putnam and Rothbart, 2006), and the *Social Skills Rating System* for social competence (Van der Oord et al., 2005). Furthermore, these scales differ per developmental period and pose challenges in their use in longitudinal studies (Denham et al., 2009). Child-robot interaction studies in education are often short-term studies and rarely deploy robots for more than a few days, according to reviews on social robots in classrooms (Rosanda and Starčič, 2019; Woo et al., 2021). Systematic, long-term evaluation of the potential negative impact of social robots’ potential on children’s social-emotional development is lacking. This might be explained by social robots still being a nascent technology. An accepted approach to evaluate the potential long term (negative) impact of nascent technology is to include stakeholders into the design and evaluation of technology (Friedman et al., 2008).

Teachers are one of the most important stakeholders when implementing social robots in education. They are not only responsible for the learning process in a classroom, but they also play a key role in children’s social-emotional development (Denham et al., 2009). They could therefore provide insights into the potential compromising role of social robots. However, in the extant literature on teachers’ perspectives on social robots, teachers have had little experience with robots (Van Ewijk et al., 2020; Xia and LeTendre, 2020; Chootongchai et al., 2021). Additionally, researchers have pointed out that the level of experience with robots could influence stakeholders’ perspectives (Serholt et al., 2014). People with experience to working with robots are significantly more likely to have a positive attitude towards social robots, compared to people with little to no experience (Smakman et al., 2021a). This makes it hard to evaluate the potential harms and benefits voiced by teachers in earlier studies.

The lack of experience of stakeholders combined with the limited empirical data, make it hard to evaluate the reported potential risks related to children’s social-emotional development. Given that studies are often short-term and stakeholders’ worries are hard to evaluate, there is a need to examine the impact that social robots have on children’s social-emotional development now that social robots are entering day-to-day education for longer periods of time. Therefore, this study aims to assess the impact of social robots in primary education on the social-emotional development of children. To this aim, we conducted in-depth interviews with teachers who have applied social robots in their day-to-day education. These primary school teachers all have a thorough knowledge of the social-emotional development of the children in their classroom, as this is part of their daily job. Therefore, in our opinion, they are most appropriate persons to assess the impact of social robots on children. Besides the impact on children’s social-emotional development, we examined which children, according to the teachers, would be more susceptive to social robots, and what the teachers would consider best practices for using social robots responsibly. In the next section, we will first describe our methodology, followed by our results. Thereafter, we will discuss our main findings in light of earlier research and discuss our conclusions.

## Materials and Methods

### Participants

For qualitative research, such as this interview study, participants can best be selected based on their understanding of the phenomenon (Kuper et al., 2008; Creswell and Creswell, 2009). Therefore, via purposeful sampling, participants were selected. The criterion for participants to be included in our study was: being a primary school teacher in the Netherlands with first-hand experience in using social robots in a real-life educational setting. Participants were recruited through newsletters of robotic companies, messages on social media, snowballing (Ghaljaie et al., 2017) and direct e-mails. Nine experienced teachers agreed to participate in our research (Mean age = 36 SD = 10, 8 Female, 1 Male). On average, they had 12 years of working experience, ranging from 1.5 to 35 years. The participants ranked their own experience with robots on a 1–5-point rating scale (1 = having very little experience and 5 = having very much experience). The mean score for the experience with robots was 3.66 (SD = 0.82). In total, the participants supervised/facilitated the child-robot interaction of 2,660 primary school children from all primary school levels/grades. General information about the teachers who participated in the interviews is shown in Table 1.

**TABLE 1:** Data on participants in the interviews.

Interview #	Gender	Age	Experience as a teacher (years)	Experience with robots (1–5 scale)	# Children interacting with a robot
1	F	39	14	4	600
2	F	25	3	3	57
3	F	36	13	2	20
4	F	42	10	5	700
5	F	57	35	4	540
6	F	28	7	3	200
7	M	39	12	4	500
8	F	25	1,5	4	25
9	F	35	14	4	18

### Materials and Measures

In setting up our interview guidelines (Taylor, 2005), we followed the five phases of the framework for the development of a qualitative semi-structured interview guide created by Kallio et al. (2016). First, we established that a semi-structured interview would be a rigorous data collection method in relation to our research question, because it allows the interviewer to improvise follow-up questions based on the teachers’ answers and it allows room for participants’ verbal expressions. Second, we created an initial set of questions targeting teachers’ perspectives on the robot’s influence on children’s social-emotional development based on existing literature. These questions included four main themes. The first questions were related to the social demographic data of the participant, such as age and gender, because these are shown to influence people’s perception of robots (European Commission, Directorate-General for Communication, 2017). The second type of questions was about how the teachers applied the robots in their classroom. These included which robot they used, but also what role the robot was given in the classroom. Earlier research has shown that children react differently to, for example, a robot as a peer, compared to that of a robot as a teacher (Zaga et al., 2015). Furthermore, role switching has also been shown to have potential as a motivational strategy (Ros et al., 2016). The third and fourth themes were related to the possible perceived social-affective bond of children with the robot and its potential influence on children’s social-emotional development. After setting up the initial interview protocol, two expert scholars in social robotics reviewed the interview guide to validate the coverage and relevance of the content. Furthermore, as prescribed by Kallio et al. (2016), the feedback of the experts was used to reformulate the questions and to test the implementation. This resulted in the final list of interview questions, which can be found online (https://osf.io/qne96/).

### Procedure and Analysis

Over a span of 2 months, from February to April 2021, the data for this study were collected. Due to the COVID19 pandemic, all interviews were conducted online via Microsoft Teams. The interviews started with a short introduction about the purpose of the study, after which the questions started. As mentioned, the interviews were semi-structured (Kallio et al., 2016), which allowed us to deviate somewhat from the formal set of interview questions when needed, and to explore the thoughts and beliefs of participants in more detail. In general, each interview lasted between 45 min and 1 h. At the end of the interview, we inquired whether participants would like to voice any other potentially relevant information related to child development and robots in education. Lastly, we asked participants if they could provide us with names of other teachers who had applied social robots in their education and might be willing to participate in this study. All interviews were recorded, for which all participants provided active verbal consent. Afterwards, the recordings were transcribed. All transcriptions were then analyzed using an inductive and deductive coding process through a qualitative data analysis application (ATLAS.ti, version 9). To identify patterns within and across the data, we used a thematic analysis method (Braun and Clarke, 2012). First, we coded the text based on the main themes of the interview questions (participant data, use of robots, social-affective bond, and social-emotional development). Thereafter, we randomly read samples of the data and created thematic codes, shown in Table 1. We then applied the codes onto new sample texts derived from our interview transcriptions. Using this iterative process, we created our final coding scheme which we applied to all data collected. The themes were coded by a scholar with considerable experience in conducting qualitative studies in social robotics and education. The final coding scheme can be found online (https://osf.io/qne96/). Lastly, the effects of the robots on children derived from the thematic analysis were linked to the appropriate domains of children’s social-emotional development reported in the literature (Denham et al., 2009). This was done during a mapping workshop by the first author and two undergraduate students.

## Results

All participants had experience with applying humanoid robots in their education, being either with the Nao robot (SoftBank Robotics, 2020) or the Alpha mini-robot (Ubtech, 2021). One participant also had experience with other types of robots, such as the Innobot, Probot, Bluebot, Microbot, and Ozobot. Experience with applying robots ranged from 6 years to a couple of months. The participants had applied the robots in their day-to-day education for teaching children arithmetic, language, geography, presentation skills, physical education, and computational thinking. Eight participants had used the robots as a social entity (as a tutor or peer), sometimes combined with using the robot purely as a tool, such as for learning programming. One participant had used the robot just as a tool for teaching programming. The number of interactions with the robot per child ranged from just one to sixteen times per period of 10 weeks. The time children had spent working with the robot ranged from 15 min to 1 hour per interaction. None of the participants systematically measured the effect of the robot during their lectures. The teachers used robots in all classes of the primary school, which included children from age 4 up to 12 years.

### Place in Education

Eight out of nine teachers mentioned that social robots (should) have a place in primary education. They considered the robot a good educational tool, mainly because it can enrich the lessons. *“Some children learn more easily from books, another child learns more easily from a screen with interactivity, and a robot gives an extra dimension to education […] it is one of the means by which you prepare children for a future”* as one teacher indicated. The teachers overall stated that they viewed the robots as additional support for the teacher, or to provide help for solving problems (such as knowledge gaps) by means of targeted help. Teachers had applied the robot in small groups and in one-to-one interaction settings. Most teachers indicated that the robot has a clear novelty effect and that children are fascinated and amazed by the robot. Most of the teachers stated that the children are enthusiastic about the robot and are (more) motivated to work and learn with the robot.

One teacher did not consider social robots to have a place in primary education, for two reasons: 1) because of the high cost and 2) because of a lack of impact in primary education. Although the teacher stated that the robot does create a deeper kind of learning, because of the social interaction, she considered the robot best for special education. In special education, the teaching methods would be more open-minded for using robots and not so restricted and formalized as in regular primary education, according to this teacher. Three other teachers also indicated that the high cost of the NAO robot was an issue. Especially for teaching programming skills, they considered nonsocial or non-humanlike robots cheaper and therefore more appropriate.

Overall, the teachers indicated that the current social robots require a lot of work from the teacher. As one teacher explained: *“It is really labour-intensive for the person who sets up and prepares the robot, and this is still an impeding factor.”* Teachers also indicated that it will take some time before other teachers are acquainted with robots because the educational methods change rapidly every few years, which also takes time to implement. Furthermore, the lack of evidence that robots are (more) effective makes it hard to convince school management to invest in the implementation of social robots, according to one teacher.

### Impact on Social-Emotional Development?

It should first be noted that none of the teachers systematically measured the robot’s effect on the children’s social-emotional development. Due to the relatively broad age range of the children that interacted with the robot (4–12 years), which covers both the primary school period and the preschool/early childhood period, and because the general developmental tasks that should be assessed in each dimension of social-emotional development differs for each developmental period, we decided to describe the perceived impact based on the themes derived from our thematic analysis (Braun and Clarke, 2012).

**FIGURE 1:**
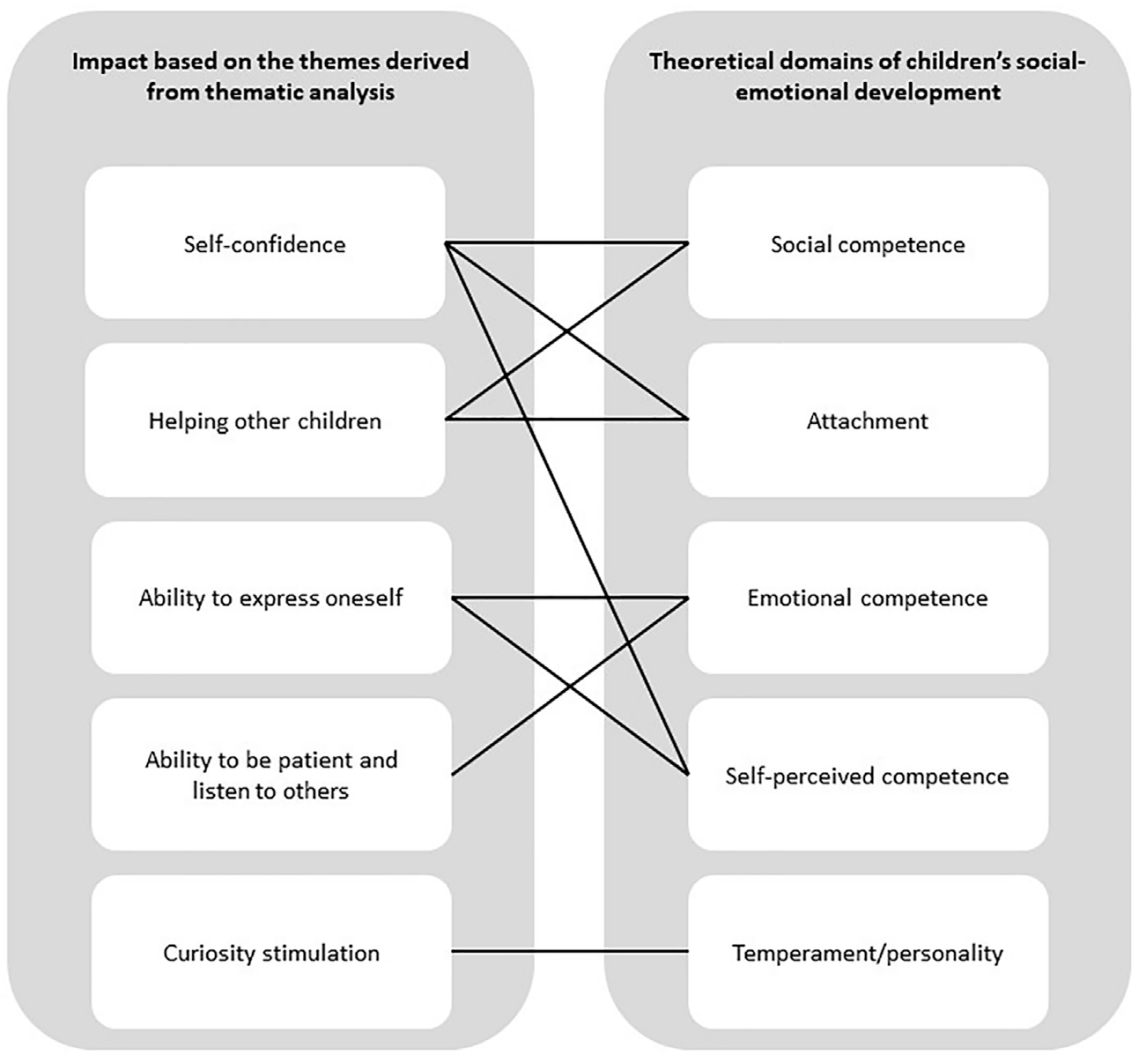
Overview of themes based on the interviews and the linked theoretical constructs of social-emotional development, based on the literature.

All teachers indicated that social robots can have a positive impact on the social-emotional development of children. They reported several examples of how children’s social-emotional development could be affected by social robots, such as by boosting children’s self-confidence and by increasing children’s ability to express themselves. All reported impact was considered positive. Only a few occasions were reported where some (mainly young) children were afraid of the robot. Based on the thematic analysis, we were able to distinguish five positive effects which were reported by the teachers, being: 1) Self-confidence, 2) helping other children, 3) ability to express oneself, 4) ability to be patient and listen to others, and 5) curiosity stimulation. Thereafter, we linked the themes to the appropriate domains of children’s social-emotional development, shown in Figure 1. In the next sections, we will present the results based on the derived themes and discuss their potential effect on the theoretical domains of children’s social-emotional development.

#### Self-Confidence

Almost half of the teachers reported higher self-confidence as a positive result of child-robot interaction. Children who were shy to talk in public or in groups could give presentations together with the robot, which could bolster the self-confidence of the children. One teacher explained: *“Giving presentations causes a lot of stress in children. I think it is good if you give them a choice, that they can give the presentation, in the first instance, completely by the robot, and then for example together, so that children, perhaps unconsciously, are presenting in front of groups. This way they will get used to it, in a very safe manner […] you actually take away a lot of stress”*. Also, teachers indicated that children who are a bit shy or socially less capable, could become the robot expert of the class, which would boost their self-confidence: *“I could put children who are socially not very strong in the spotlight so that they would become a robot expert. They were then able to teach other children or help the teacher, so they grew in their whole being because of this … , this changed their [social] position and place in the group”,* as one teacher explained. Furthermore, teachers reported that children more easily practice subjects they find difficult with the robot because a robot does not judge or laugh at them when they give a wrong answer. This is also reported to create more social interaction between children, as one teacher who used the robots for extra support in language learning described: “*We have a school where several children come from a different culture. They have difficulty speaking Dutch, and they don’t speak Dutch at home. They find it difficult to speak in public, and a robot helps them with this and thus helps with their own language development, which also makes it easier for them to make contact with peers. That is what we have seen, it absolutely had an impact*”. None of the teachers reported negative outcomes related to the self-confidence of children. Although, some teachers reported practical issues related to the speech of the robot that sometimes lacks the proper pronunciation, especially with longer words.

The capability of the robot to contribute to children’s self-confidence can be (in)directly linked to three of the five social-emotional domains. First, the increased social interaction between children, caused by the increased self-confidence of shy children, could lead to the formation of dyadic friendships, which is linked to the social-emotional domain of *social competence.* Furthermore, this could lead to a more balanced connection with their peers, which is related to the social-emotional domain of *attachment*. Lastly, the robot could contribute to the domain of *self-perceived competence*, because it could result in a child’s increased ability to assess one’s own social abilities in comparison with those of others.

#### Helping Other Children

Several teachers indicated that they applied the robot to enhance social interaction between children. For example, by giving some children the role of robot expert, they created a new role in the group. According to teachers, this did not only increase children self-confidence (cf. *Self-Confidence*), but it also allowed the robot experts (often the socially weaker children) to more easily interact with other classmates. Also, by letting children work with the robot in small groups, the interaction between children in the groups was stimulated. Furthermore, when the robot was used by multiple groups in sequence, the last group could help the next group when they encountered difficulties. One teacher expressed concerns about when the robot would be used for one-on-one tutoring, which could potentially lower the contact with other children. The teacher considered this as part of the broader trend of (smart)phone use and time spent on a computer, which seems to lower personal, face-to-face contact. However, the teacher could not tell whether the robot caused children to interact less with each other. Likewise, this was not reported by any of the other interviewed teachers.

The option to apply a social robot to stimulate helping behavior can be (in)directly linked to two of the five social-emotional domains. The introduction of social robots, which allows for the creation of new roles in the classroom as indicated by the teachers to stimulate interaction, and can be linked to the domains of *social competence* and *attachment*.

#### Ability to Express Oneself

Some teachers reported on children who, before the introduction of the robot, would not be willing to talk to the teacher, or did not want to learn. However, after the robot was introduced in the classroom, these children started to talk. First to the robot, and thereafter to the teacher. Teachers said that they expected that children would more easily express certain things to robots than to their teachers. *“I think that a robot could definitely be used for that [emotional support] as well […] because it is something that is a bit further away from you and a bit less personal, so I think it is easier to discuss more difficult things […] and certainly in the social, emotional area,”* as voiced by one of the teachers. Some teachers used the robot as a means to let children talk about their feelings by letting the robot express emotions. This has led to the opportunity to talk about emotional feelings. One teacher compared this to hand puppets that are currently used in the Dutch educational system to start confirmations on difficult subjects, which the teacher considered a similar tool.

Children opening up to a robot about their feelings relates to two of the five social-emotional domains. First, it could allow children to cope with negative emotions, learn about emotions and emotional expressiveness, which is linked to *emotional competence*. Second, it allows for the possibility for children to get more insight into their own social competence, which is related to the domain of *self-perceived competence*.

#### Ability to be Patient and Listen to Others

Two of the teachers reported on the robot’s ability to teach children to be patient and listen more carefully to others. This was mainly caused by the robot’s script that did not allow a child to go any faster, according to the teachers. “*You have to keep calm and you also have to keep your impulses in check […] you also have to be careful, children are normally rumbling everywhere, in a manner of speaking, but that is really not possible. So yes, there is really something being asked of them*”, as one teacher reported. The teachers indicated that the robot made children listen more to others and wait their turn. However, they also indicated that the robot would need to be in the classroom for longer periods to make a lasting impact on these skills.

The ability to be patient and more carefully listen to others could, in theory, contribute to understanding the unique perspective of others, which can be linked to *emotional competence*.

#### Curiosity Stimulation

Several teachers indicated that they have seen how robots can stimulate children’s curiosity. Most teachers reported on the robot being something “magical” or “special”. This made children curious to learn about and from the robot, also for subjects they would otherwise dislike or even avoid. One teacher experienced the following: “*I had one child at that time, who did not want to learn. That does not happen often, but he really did not want to, he had no interest at all in reading or in letters or in math or something else, but that robot that was really it. Once that robot was there, he did everything. That was so special, he did everything he had to do, but not with me, but with the robot. With me, he just closed down, but with the robot, he did it all.”* The teacher indicated that she did not encounter this behavior often. Other teachers mentioned that they had also experienced how robots stimulated and motivated children, although they voiced that they did not consider the currently limited interactions enough to have a long-lasting effect on children’s curiosity.

The ability of the robot to stimulate curiosity can contribute to the social-emotional domain of temperament/personality. By stimulating children’s curiosity, they could become more encouraged to follow and experience aspects that suit their personality, which can be linked to the *temperament/personality* domain.

In summary, the teachers expressed five ways by which social robots can impact the theoretical domains of children’s social-emotional development, which is illustrated in Figure 1. Children potentially getting attached to the robot was a topic that came up regularly during the interviews. Therefore, we decided to discuss attachment as a topic separately in the next section.

### Attachment

Almost half of the teachers indicated that children can feel emotionally attached to a robot. Some indicated that this attachment would not be different from how children attach to other objects children like, such as video games and toys. One teacher saw a child with bonding problems getting emotionally attached to the robot, but did not encounter this with other (typically developing) children. Some teachers indicated that while young children could feel attached to the robot, older children, around the age of 11-12, would consider the robots merely as a tool.

Another teacher reported on a child talking about the robot as his best friend, while other participants indicated that they have seen children interact with the robot as if it were their buddy. In several interviews, teachers indicated that children showed a kind of empathy and affection towards the robot. As one teacher experienced: “*They [children] also immediately asked when he [the robot] would come back, and everyone wanted to take care of it, you really noticed that the care aspect really came up there. Such that it had actually become a kind of a buddy.”* Another teacher indicated to be concerned that children would view the robot as a best friend, however, this teacher did not encounter this in her own classroom. Furthermore, several teachers indicated that for children to become attached to a robot, the robot would have to be present much more often than is possible in the current educational system.

#### What Would be Considered “Too Attached”?

When asked for signals that would indicate that children are too attached to the robot, teachers expressed two main indicators: 1) when it results in less contact with their human peers, and 2) when children would get upset when the robot was not around. However, four teachers indicated explicitly that they have not encountered this in their classes, and that the way robots are being applied nowadays poses little risk for children to become too attached. *“In the current education you don’t get it [attachment issues] very quickly, only if you always have a robot in class”* and *“I see few risks in the way in which we now use robots”* as explained by two other teachers. The other five teachers did mention encountering attachment issues in their classes.

Although the teachers did not encounter children becoming too attached to the social robots, this might be due to the short interaction time and the limited number of interactions children had with the robot. Therefore, we continued to further ask the teachers on what type of children would be more susceptible to getting attached to social robots.

#### Children who are More Susceptible to Getting Attached to Social Robots

The current literature does not provide a solid basis for deriving insights into what kind of children would be more susceptive to getting attached to social robots. To gain more insight into which children might be at risk to become ‘too attached’ to a social robot, we conducted a thematic analysis to differentiate between types of children based on the interview transcripts (Braun and Clarke, 2012). The teachers expressed four types of children who would be more susceptible to getting attached to social robots.

• The first type, indicated by seven of the nine teachers, is timid, socially less strong, and could have an autism spectrum disorder (ASD). However, regarding ASD, it should be noted that one teacher explicitly stated that these usually are children of which the teachers *think* they have ASD because it is mostly not yet diagnosed at this young age. Indeed, a number of studies reported successful interactions of social robots specifically focusing on children with ASD (e.g., Huijnen et al., 2016; Di Nuovo et al., 2020).
• The second type of children concerns children who are interested in science and engineering. “*The children who are just very interested in robots and programming”*, as one teacher explained. This is in line with common applications of robots for STEM education (e.g., Ahmad et al., 2020).
• The third type of children that can be considered more sensitive for the robot’s interaction, as indicated by two teachers, are children who are underachievers on a certain subject, such as language learning or math. Studies indeed reported good results for language learning (Vogt et al., 2019; Konijn et al., 2021) or rehearsing the times tables (Konijn and Hoorn, 2020).
• The fourth and final type of children who are more sensitive to social robots are children with special needs, such as children with attention deficit hyperactivity disorder (ADHD), highly sensitive children, highly gifted children, and children sensitive to game addiction. Seven teachers indicated that these children can be considered more sensitive for child-robot interaction in education: “*The children who have a certain need […] children with ADHD, or just children who are highly gifted, they could be attracted in a certain way if it suits them, and then there are many possibilities to work with this,”* as explained by one teacher*.* In earlier studies the potential for children with ADHD have been discussed before (e.g., Fridin and Yaakobi, 2011)

The teachers expressed several best practices to ensure that these types of children would not get too attached to the robot. The best practices expressed also included general remarks on how social robots could be implemented in a responsible way, according to these experienced teachers. In the next section, we present these findings.

### Best Practices and Success Factors for Child-Robot Interaction in Education

The interviewed teachers reported about what they considered best practices and success factors when applying social robots in primary education. In total, they reported eight best practices and success factors for applying social robots in primary education. To provide an overview of these best practices and their description we present them in Table 2.

**TABLE 2:** Best practices and success factors for applying social robots in primary education.

#	Title	Description
1	Apply when needed	Make sure there is a clear *why* for applying social robots, robots are means not ends. Social robots are considered to be an addition to the teacher, not a replacement. When applying the robot every day, the novelty effect can wear off. Use social robots for a specific aim or goal
2	Teacher stays involved	The role of the teachers stays very important, he/she should be present during the child-robot interaction, or at least close by. Also, the teacher can judge which children potentially get too attached to the robot, and which children would benefit most from the interaction. This might lead to an increase in the number of teaching assistants needed to facilitate the robot interaction
3	Proper introduction	Teachers should pay specific attention to the introduction of the robot. Children should first be told what a robot is, and what is it going to do, before they start to interact with a robot
4	Small groups	Learning with robots is best done in small groups. This not only allows children to continue communicating with their peers, but it can also stimulate children to interact with each other and not get socially isolated
5	Vertical groups	Let children of different age groups work together with the robot, make use of the older, more experienced children to introduce and guide younger children
6	Separate room	When a small group of children is working with the robot, this is distracting for the other children in the classroom. Therefore, the robot should not be in the same room as where other children are who do not work with the robot
7	Team effort and mindset	For robots to be sustainably implemented in schools, the technology needs to have the support of the teacher-team including the school management. A teacher in the role of a robot ambassador can be appointed to introduce the robot to other teachers, making it easier to implement the robot
8	Parents	The parents of the children should be informed pro-actively by the schools when social robots are going to be used. This is the responsibility of the school

## Discussion

The main goal of this interview study was to examine whether social robots in primary education compromise the social-emotional development of children. Therefore, we interviewed primary school teachers who supervised the child-robot interaction of more than 2,600 unique children in a real-life school environment. Nearly all child-robot interactions reported by our interviewees were one-on-one or small group interactions in which a humanoid robot took the role of a tutor or peer. Each robot was used for teaching children a specific subject or skill in a school environment.

The main finding of our study is that the participating teachers experienced no negative effects on the social-emotional development of children caused by the child-robot interactions that would have a lasting negative impact. In contrast, teachers expressed seeing five positive effects of social robots related to the social-emotional development of their pupils, being 1) increased self-confidence, 2) helping other children, 3) increased ability to express oneself, 4) increased ability to be patient and listen to others, and 5) curiosity stimulation. These five themes could be linked to all domains of children’s social development reported in developmental literature, as discussed in the introduction and summarized in Figure 1.

The social robots seemed especially useful for introducing the learning by teaching paradigm (Fiorella and Mayer, 2013). This allows for some children to take on new roles, such as that of an expert. This can have a positive effect on children’s social-emotional development. For example, by giving children an expert role, or by letting experienced groups help other groups. Novel technologies, such as social robots, seem appropriate to support children in such roles. The robot’s impact on the children’s ability to be patient and to listen carefully was reported to be caused mainly by the current state of technology that does not allow children to respond quickly, and due to the intonation of the robot which is sometimes lacking. Given that automatic speech recognition based on child-robot interaction has been shown to be a complex issue (Kennedy et al., 2017), it is unlikely that robots will be able to respond quickly to children’s verbal reactions in the near future. Therefore, we consider that the robot’s positive impact on children’s ability to be patient and to listen will remain for the foreseeable future. However, teachers indicated that they wondered whether the effect on children’s ability to be patient and to listen would impact the children in the long run. The other three effects, increased self-confidence, ability to express oneself, and curiosity stimulation, seem all specifically useful for children with special needs.

Four types of children were identified by the interviewed teachers, three of whom could specifically benefit from social robots and be receptive to interacting with a social robot. These children are considered to have special needs, either the timid, socially less strong children potentially with ASD, underachievers, or children with other special needs, such as ADHD or attachment issues. According to the teachers, these children could potentially benefit the most from social robots in education when it comes to their social-emotional development and are indeed often addressed in studies (e.g., Fridin and Yaakobi, 2011; Huijnen et al., 2016; Konijn and Hoorn, 2020). As a downside, the interviewed teachers reported that these children might get more attached to the robot in the long run, which could, in theory, lead to less human contact and children getting upset when the robot would not be around. However, this has not been observed by our teachers, and they further indicated that the robot would need to be present much more for this to occur.

To ensure that some children will not get too attached to the robot, teachers have indicated that they should supervise the child-robot interaction, or at least be close by. The teachers in our study mentioned that applying social robots in education is labor-intensive, and requires time and effort to use and implement. This is in line with another study reporting about teachers being worried that social robots would increase the workload of teachers (Reich-Stiebert and Eyssel, 2016). A recent review on robots in classrooms came to similar results, concluding that “the current generation of commercially available robots, like NAO or Pepper, do not have sufficient programming to be readily integrated into classrooms without extensive support and resource mobilization” (Woo et al., 2021, p.9).

The comparison of the bond between children and robots to the bond between children and other humans might not be the best way forward. Although some children seem to behave as if they are friends with a robot (Fior et al., 2010), robots are still a different entity. When comparing human-robot interaction to interaction between humans, Black (2019) argues against developing empathy with robots because children cannot experience the kind of affect toward robots that they develop with other humans, such as their human peers and teachers. However, if we use the robot to simulate human interaction, by letting children work together, this doesn’t seem to be a big problem. Furthermore, for social robots to be able to support children in primary education, there seems to be no need for very humanlike robots with extensive empathy capabilities; current studies on the use of social robots in education do, most of the time, not use very humanlike robots with extensive empathy capabilities, and still show promising results (e.g., Konijn and Hoorn, 2020). One might argue that robots need extensive empathy capabilities for teaching social skills to children who cannot learn these with their human peers because of disorders, such as ASD. Although humanoid robots with extensive empathy capabilities might help this specific group of children, there seems little reason to equip robots with far-reaching human embodiment when it comes to assisting regular children in their school process.

The social bond between child and robot challenges the fundamentals of friendship and relationships, according to Richards and Calvert (2017). However, according to the teachers in our study, such social bonds are infrequent and similar to the bond children have with other technologies or artefacts, such as smartphones and (hand) puppets. Thus, the negative impact of social robots on the fundamentals of friendship and relationships, for now, seems limited.

Other researchers have found that robots seem able to elicit socially desirable behavior among children, such as sharing (Peter et al., 2021). However, according to the same researchers, this may also apply to socially undesirable behavior, such as aggressive behavior (Peter et al., 2021). Children have been recorded to express bullying behavior towards an educational robot (Kanda et al., 2012). Others have also expressed concerns related to the robot becoming a bully or becoming subject to bullying (Diep et al., 2015). However, following the best practices of the participants in our study, when teachers stay involved in the child-robot interaction, this scenario seems unlikely. Teachers or teaching assistants could intervene when such undesirable behavior occurs. Nevertheless, the results of other researchers emphasize the importance to be careful in how robots are presented to children because robots (in videos) have been shown to negatively influence children’s pro-social behavior and willingness to share resources in an experimental setting (Nijssen et al., 2021).

The participating teachers did not report major privacy issues related to the child-robot interaction, except one related to IT security, and they did not use extensive personalized data collection by the robot. This might be due to the relatively simple, not highly personalized child robot interaction currently used in schools. In other studies, privacy has been reported to be a major issue related to social robots in education (Sharkey, 2016; Smakman et al., 2021a). Data collection allows personalized interaction, which is one of the key benefits, according to scholars (Kanda et al., 2012; Shimada et al., 2012; Jones et al., 2017; Jones and Castellano, 2018; Woo et al., 2021). Although the teachers in our study did not report on major privacy issues, given the need for data collection for personalized learning, we consider the issue crucial for integrating social robots in education in a responsible way and should therefore be subject for further research.

One limitation of this study is that, although the participants had experience with using a social robot in their day-to-day education and supervised the child-robot interaction of over 2,600 unique children, the total number of participants was limited. However, given that all participants had experience with using a social robot in their day-to-day education, combined with the large number of unique children they supervised, they still provide valuable insights into the currently observed effects of social robots on children. The gender distribution was unequally balanced, with only one male participating teacher. However, this can be considered a reflection of the gender distribution in Dutch primary education, where approximately 80% is female (Traag, 2018). It should also be noted that this study was carried out solely in the Netherlands, therefore the results may differ in other countries. Furthermore, none of the teachers systematically measured the robot’s effect on the social-emotional development of children. The evaluations in this study are solely based on the teachers’ previous experiences and observations. The experiences of these teachers could differ from how children experienced the robot interaction. Further studies could compare the perceptions of children to the perceptions of their teachers. Future studies in child-robot interaction could also include the Social Skills Rating System (SSRS) or the social Skills Improvement System-Rating Scales (SSIS-RS) (Gresham et al., 2011), to systematically measure the impact of social robots in children’s development.

In conclusion, our study indicates that the social robots currently used in education pose little threat to the social-emotional development of children according to teachers who applied these robots in their day-to-day education. Children with special needs seem to be more sensitive to social bonding with a robot compared to regular children. However, this social-affective bond seems to have more positive effects enabling them to more easily connect with their human peers and teachers.

Given that the best practices reported in this study are taken into account, we consider that social robots pose more benefits than harms concerning the social-emotional development of children. However, when robots are being introduced more regularly, daily, without the involvement of a human teacher, new issues could arise. For now, given the current state of technology and the way social robots are being applied, other (ethical) issues seem to be more urgent, such as privacy and security issues, and the workload of teachers.

## Data Availability

The datasets presented in this study can be found in online repositories. The names of the repository/repositories and accession number(s) can be found in the article/Supplementary Material.
