# Supplementary material for: Do Robotic Tutors Compromise the Social-Emotional Development of Children?
Source: Front Robot AI. 2022 Jan 21;9:734955. doi: 10.3389/frobt.2022.734955 (PMC8814517; doi:10.3389/frobt.2022.734955)
Supplement: Supplementary file 1 [file DataSheet1.DOC]

# Project: Interview study - social bonding/skills social robots

Report created by Matthijs Smakman on 1-7-2021

**Code Report**

Selected codes (56)

**
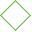
 ○ Attachement, usefull .**

**12 Quotations:**

**
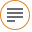
 2:31 ¶ 81 – 83 in Interview 2 12-02-2021.mp4--edited**

Ehm ja, alpha mini zou ikzelf vooral echt inzetten, want wat ik ook al zijn met de emoties bespreken, dus dan kun je meer het gesprek aangaan met de kinderen ehm. Maar ik denk ook dat jij kunt inzetten door kinderen gewoon te laten proberen. Laat hem gewoon mensen die dingen doen, die wil dat hij doet en hadden er plezier uit en hoe meer je eruit kunnen halen, hoe meer binding je er. ook meekrijgt omdat je snapt hem een beetje beter weten hoe hij werkt. Je kunt wel meer dingen uit te halen ehm en bij die PLEO is het gewoon daar, daar zie je eigenlijk gewoon al heel snel binding mee, gewoon omdat die op zich. Je hoeft niet heel vaardig te zijn op het gebied van het programmeren van van deze robot, want de stap om wat te laten doen zijn heel laag. Maar ja, hij doet eigenlijk meteen meteen wat jij vraagt en dat vinden kinderen ook meteen heel erg leuk. Dus ja, welke stappen dat vooral op sociaal emotioneel gebied zijn, ja, ik ik. Ik zou die de Alpha mini vooral inzetten om meer om het programeerstuk functionaliteit eruit halen en daarmee link een verbinding vinden met kinderen die dat wat meer zoeken, terwijl die olifant eigenlijk al voor de vaardigere kinderen meer enthousiasme kan opwekken voor het gebruiken van robots – en dan vooral het met elkaar er over te hebben. Laat ze vooral samen zo'n robot uitvogelen, ik denk dat dat ook vooral wat teweeg brent.

00:30:14 MS: Brengt dus de samenwerking tussen de kinderen samen met de robot.

00:30:19 Interview participant 2: Absoluut absoluut.

**
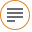
 2:49 ¶ 65 in Interview 2 12-02-2021.mp4--edited**

. Maar ja, toen moest ik hem wegstoppen en ik denk ik kan het niet uitzetten en in een doos pleuren en weg weet je wel, je moet wel de fantasie wel wel levend houden. Dus dan ging hij lekker slapen. En dan deden we een dekentje er over heen en zijn een koekje weg en deksel erop – en ze vonden dat super jammer – dat ie wegging ze hadden echt zoiets, hij mag

**
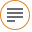
 3:30 ¶ 94 – 97 in Interview 1.mp4--edited**

Ja, want ik al zei de rol van de mens, vind ik altijd heel belangrijk, nog steeds vooral bij jonge kinderen, om het te nuanceren voor hun op te laten begrijpen hoe het zit, omdat ze dat dat begrip nog niet hebben. Mijn oudere kinderen vind ik het ook van belang om een stukje dan weer bewustwording te laten geven van. Maar waarom doen we dit dan.

00:31:09 MS: Ja.

00:31:10 Interview participant 1: Dus het is vooral bij de bovenbouw zou ik als reflectie pakken, de volwassenen leren ook moeten aanleren. Dingen is puur praktisch, maar ook een stukje reflectie en het het ja, ik vind ik – vind die rol van de docent of de de volwassenen op de de de mens erbij vind ik wel heel belangrijk, niet altijd samen dat ze er samen zijn, maar dat er naderhand op daarvoor wel een gesprek plaatsvindt over wat er gaat of is gebeurd, of hoe het ging en wat het dan met het kind – want dat zijn de interessante aspecten – vind ik altijd wat ze ervan. En dan weet je ook of je een keer de minder gang moet laten gaan of juist niet niet. Alle kinderen vinden het leuk om met er wel wat aan de gang te gaan. En dus ja, die de mens vind. Ik, dat vind ik één van de belangrijkste dingen. Als je je gaat zeggen dat je altijd iemand hebt die echt weten wat voor effect kan hebben op een bepaalde leeftijdcategorie, zeg maar wat dat vind ik wel heel belangrijk. Niet alle leerkrachten hebben, dat, zeg maar.

00:32:11

**
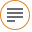
 5:24 ¶ 168 in Interview 3 17-02-2021**

In combinatie met een leerkracht of volwassenen, iedere klas een robot en structureel inzetten, ouders zijnnog niet toegerust om dit te waarborgen, school heeft een verantwoordelijkheid om ze goed voor te lichten. Robots ook gebruiken voor 21st c. skills, programeren. Als je robots gaan inzetten is er ruimte voor nodig in de school, en tijd. Ook een duidelijke Why, niet zoals nu eerst de tech en daarna een doel zoeken.

**
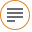
 5:25 ¶ 173 in Interview 3 17-02-2021**

Kinderen niet altijd laten werken met de robot, dat de robot niet de focus wordt van het leven van kinderen.

**
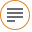
 7:13 ¶ 139 in Interview 5 3-3-2021.mp4--edited**

Ik weet het niet, ja sommige kinderen die vinden, er zijn natuulijk ook kinderen die die met de leerkracht of met iemand anders het moeilijk vinden om zichzelf te geven, want een een persoon dat dat ervaren ze toch wel, hoort anders dan zo'n robot, dus daarin als kinderen het echt spannend vinden om dingen te doen en je laat ze alleen met de robot aan de gang. Dan kun je natuurlijk wel krijgen – dat heb ik nog niet ervaren – dat kinderen daardoor wel verder durven te gaan. Maar dan moet je, dan zit je best wel in kinderen die heel veel problemen hebben, dat ze dat ze iets, zich niet durven te geven of iets niet durven te doen omdat de leerkracht het dan hoort. Dan durven ze misschien wel tegen een robot te zeggen, maar dat is meer, dat heb ik nog niet ervaren op school, snap je wat ik bedoel? Die faalangst die die komt dan tevoorschijn komt als als andere mensen iets van jou horen, waarvan je niet zeker weten of het goed is, een robot, ik kan me voorstellen dat ze dat wel kunnen ervaren, dat een robot niet oordeelt. En dat kan fijn zijn. Natuurlijk. Maar wij hebben hem nog niet zo ingezet, maar ik kan me voorstellen dat misschien in het speciaal onderwijs dat dat veel sterker is, wel veel eerder iets zou zijn waarvoor je hem zou kunnen gebruiken.

**
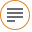
 8:9 ¶ 89 – 91 in Interview 6 12-03-2021.mp4--edited**

de limitering in gewoon de beschikbaarheid en de tijd dat een kind kan interacteren met een robot en het inzetten door middel van een samenwerking opdrachten.

00:29:57 Interview participant 6: En gelimiteerd is natuurlijk eigenlijk, daar ontkom je ook niet aan, want je moet ook gewoon rekenen en taal doen, zeg maar, dus, de tijd is gelimiteerd.

00:30:04 MS: Eigenlijk zeg je, in de in de praktijk kan dat al niet voorkomen, dat het continu zo is, want ja, de , we zitten gewoon in een schoolsysteem.

**
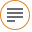
 9:5 ¶ 46 in Interview 7 12-03-2021.mp4--edited**

Ja, ik denk wel tot tot en met de kleuters moet je daar wel echt heel voorzichtig mee zijn, zodat je dat heel, je kan niet zo'n, apparaat zomaar in de klas zetten en zeg maar bij wijze van spreken, weglopen en kijken wat er gebeurt. Dan denk ik echt dat je dat kinderen slecht slapen 's nachts. Dus vanaf groep drie, vier dan dan zal dat wat afnemen. Maar dat is ook wat wij wel gezien hebben hoor in, de Tamara mijn collega, die heeft ook gewerkt met jonge kinderen die zegt, daar moet je echt voorzichtig mee zijn. Dus ja, dat zou ik ook zeker doen. Ik heb het zelf dus thuis ook gezien, was geen succes robot aanzetten waar hij bij was.

**
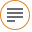
 9:17 ¶ 98 in Interview 7 12-03-2021.mp4--edited**

: Nou, je zou een robot dan in een kringgesprek kunnen laten deelnemen of in een groepje kinderen om dingen te te bespreken dat je de robot. Ja, dat zal vooral over vragen gaan, vragen laat stellen of zo, maar het doel blijft om kinderen met elkaar te laten communiceren. Dat moet eigenlijk al voorop staan. Er zijn misschien wel meer manieren, maar die kan ik even niet zo één twee drie, zie ik nog even niet zo op die manier.

**
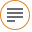
 10:24 ¶ 114 in Interview 8 31-03-2021--edited**

: Hmm nee, ik denk dat je er wel goed voor moet maken dat het er niet altijd is, want dan, het moet wel een ook een speciaal ding zijn, denk ik, want als het te normaal wordt dan gaan kinderen er misschien ook geen zin meer in hebbeen, terwijl, het nu juist een boost geeft om te willen leren, denk ik.

**
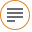
 10:26 ¶ 120 – 123 in Interview 8 31-03-2021--edited**

Als je dat met gezichtsherkenning gebruik, dan denk ik wel dat de robot een kind echt wel kan sturen daarin ja.

00:27:22 MS: Hoe zou je dat voor je zien?

00:27:28 Interview participant 8: Bij lezen, als je woord goed gelezen hebt, dat die dan, goed, en je doet goed mee, dat soort dingen.

00:27:34 MS: Oké, eigenlijk motiverend ook optreden. Hoe zouden we er nou voor kunnen zorgen dat kinderen niet te gehecht raken aan robots?

**
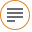
 10:27 ¶ 126 in Interview 8 31-03-2021--edited**

Ja, ik denk zelf ook wel in de gaten houden van de hoeveelheid tijd en de opdrachten die je waarbij je hem in gaat zetten, zeg maar, en robots zelf... ik vind het lastig, want dat motiveren moet je misschien niet bij elke opdracht doen, maar het is wel goed als die het wel doet, dus je moet het ook niet, ja, ik denk dat je een goede balans ergens moet zoeken, maar er waar de balans is, dat weet ik ook mee.

**
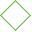
 ○ Attachement_more susceptible**

**11 Quotations:**

**
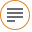
 2:29 ¶ 71 – 75 in Interview 2 12-02-2021.mp4--edited**

Absoluut absoluut, ik denk – dat zag ik bij die roze olifant natuurlijk ook wel – dat sommige kinderen meer trokken dan andere kinderen of die... een aantal kinderen waren. In eerste instantie hadden niet zoiets mee. Hadden er niks, niet zoveel daarmee. Maar ze waren stiekem heel nieuwsgierig, maar zn durfde niet te veel te laten zien en dan komt er uiteindelijk toch wel naar boven. Ja, sommige kinderen zijn er misschien wel sneller op uitgekeken dan andere. Dat heb je ook. Hè.

00:27:05 MS: Wat kenmerkt de kinderen, die er als eerder te trekken?

00:27:11 Interview participant 2: Nou viel mij sowieso op dat het vooral de meisjes waren die daar snel op op af ging. Bij af alphamini was het eigenlijk. Ja is zowel bij de jongens en meisjes enthousiast, maar misschien bij de roze olifant, omdat het een schattige robot was dat de jongens nog sneller zoiets hebben van, ik zie wel wat die kan.

00:27:29 MS: Ja.

00:27:30 Interview participant 2: Dus dat trok eerste de meisjes er erg naar toe, en de leerlingen die wat teruggetrokken er waren. Die waren vanzichzelf. Sowieso denk ik al wat rustiger Het waren een paar die die ook op sociaal emotioneel gebied heel teruggetrokken zijn. Maar dat waren de leerlingen die vervolgens heel actief met de robotarm aan de slag gingen. Dus die hadden misschien niet zozeer die binding nodig. Die wilde vooral de functionaliteit van de robotarm wil zien. Dat vonden zij dan belangrijker. Dus, ja, dat is denk ik ook echt wel het kind eigen waar hebben ze vroeger mee gespeeld? Wat vinden ze interessant? Vinden zij functionaliteit belangrijker dan dan dat ze echt binding? Dan ben je willen hebben. Sommige kinderen hoeven dan niet eens binding mee te hebben, denk ik die willen gewoon zien die kan.

**
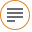
 2:30 ¶ 76 – 77 in Interview 2 12-02-2021.mp4--edited**

MS: Ja, wat je zegt je, je noemt, die, noemt die kinderen ook eerder, ook die sociaal minder vaardige kinderen, dat die ook geïnteresseerd waren in de robots, maar mogelijk meer op de functionele kant van van de robot. Dan als de sociale kant.

00:28:28 Interview participant 2: Ja, precies.

**
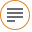
 3:27 ¶ 69 – 72 in Interview 1.mp4--edited**

: Ja, en je hebt dus ook zelf gezien en je hebt wel uitgelegd wat je hebt gezien zijn dr. Kinderen denk je die hier ook weer meer gevoelig voor zijn dan andere kinderen.

00:23:29 Interview participant 1: Ja, ja, ook weer die die die wat sociaal zwakkeren, die wat onzeker dat die kinderen, die waren daar heel erg gevoelig voor. En ik was iets magisch. Ook met veel fantasie mn werd ik het ook interessant. Mijn dochters hebben ook ik heb ook heel een Ronald die thuis af en toe – en die zijn ook altijd heel erg mee begaan. Zeg maar die dat mijn kinderen heel zwak zijn, maar ze zijn er echt niet opgegroeid. Zeg maar de afgelopen jaren met meerdere robotjes hier thuis en ze zijn ook altijd wel een soort van zorgzaam. Ook omdat het een dure apparaten zijn, maar ook omdat ze weten dat ze wat leuks mee kunnen doen, is het leuke dingen doen. Dus het zorgt ervoor dat ze wel ja, niet niet alle kinderen hebben, maar ze moeten dan lange tijd heb ik idee met zn apparaatje in aanraking komen en wat ik denk ook dat is dat de fantasie hoe erg meespeelt. Mijn mijn is een heel fantasierijk gaan is en ehm. Ja, die kunnen zich heel erg mooi verliezen in in al die dingen, dus ook dat een robot die ziel heeft zon gesprek hebben ook wel eens gehad met elkaar in. Hoe dan? Neem maar wat ik de inzetten. Dus ja, dat is echt bij welk type klanten zeg, maar ja.

00:24:45 MS: En dat dat dat zijn dus de de wat meer wat sociaal zwakkeren of de kinderen met met veel fantasie.

00:24:52 Interview participant 1: Ja, ja, als ik het zo in de in de beleving zie. Wel ja, degene die al heel mail met techniek leuk vinden, zeg maar niet in de technische kant in, of die daar wel heel erg getrokken door raken, ook altijd bezig met dingen uit elkaar halen of zo maar dat soort kinderen in die zijn ook altijd heel erg begaan met zn apparaat, het is toch wel heel heel magisch om.

**
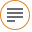
 7:15 ¶ 145 – 147 in Interview 5 3-3-2021.mp4--edited**

ja dat denk ik, kijk, ik heb wel een meisje in de klas. Nu hebben we weer – is natuurlijk een tijd niet geweest, die moeite heeft om weer praten tegen mij. Als ze dan weer een paar weken hier is, dan gaat het weer makkelijker het is natuurlijk een kleuter, hè dus, die hebben dat wel eens vaker, dit probleem gaat oplossen als het groter is, maar er zijn best wel kinderen die soms heel veel moeite hebben om, die dan toch toch een soort van vorm van autisme of of vorm van... Nou ja goed allerlei, er is natuurlijk van alles wat er kan zijn als je het over labels hebt. Maar ja, dat soort kinderen zou er juist misschien geholpen zijn door gewoon eens een keer een spelletje met de robot te doen.

00:45:01 MS: Dus dan heb je het over wat wat schuchtere kinderen of kinderen, in het autistisch spectrum.

00:45:07 Interview participant 5: Ja, bijvoorbeeld.

**
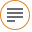
 8:6 ¶ 80 in Interview 6 12-03-2021.mp4--edited**

Mmm ja, ik kan daar gewoon een aantal jongens die het programeren echt heel leuk vonden, en dat ook echt leuk vonden om uit te zoeken, en dat je ook zei: zoek het maar uit en op gegeven moment wisten ze er bij wijze van meer van dan ik, en dat waren wel de, met name een jongen, was wel gewoon de wat slimmere jongen, maar die was sociaal ook goed, die lag ook gewoon goed in de groep ehm. En ja, ik weet niet, het kan natuurlijk een beetje soort twee kanten op gaan. Het kan natuurlijk zijn dat de einzelfganger, het type nerd, dat die wat heeft aan de sociale robot, het kan, ook zijn dat hij denk, nou ja, ik vind het leuk om te programmeren maar die uitvoerder daarvan dat, dan neem ik voor lief, soort. Als ik naar mezelf kijk, ik vind dat programmeren heel leuk, maar ik ben geen type nerd, zeg maar, zoals je het soort zwartwit zou schetsen. Dus.

**
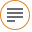
 8:15 ¶ 106 in Interview 6 12-03-2021.mp4--edited**

Ja. Dan denk ik moet name wel de kinderen die nou ja, wij bijvoorbeeld hebben we nu ook een jongen in in groep acht, game verslaafd geweest, blijf daar gevoelig voor, ja die type kinderen denk ik dat daar wel gevoeliger voor zijn, of juist de kinderen met autisme voor wie sociale contacten heel moeilijk zijn. Ja, dat kan natuurlijk deels een hulpmiddel zijn, hè, want ze kunnen voor hun een redelijk makkelijke manier toch sociale contacten hebben, maar ik denk dat dat ook wel een valkuil kan kan zijn, dat is ook niet worden gestimuleerd om de andere menselijke contacten te behouden of op te bouwen, of energie in te steken zeg maar – en dat is denk ik, een afweging die gewoon heel gedegen moet worden gemaakt, zeker bij bepaalde problematiek van kinderen of bij bepaalde gevoeligheden. Maar goed, dat is op veel gebieden. Als een kind heel gevoelig is voor... bepaalde meiden die queen bee gedrag hebben, ja, dan moet je daarin ook gaan kijken van oké hebt naast die zetten we dit kind wel. En dat is bij deze kinderen wel, als je weet hoe gevoelig zijn voor invloed van technologie en ja, hoeveel lager is dan in aanraking komen met de computer en met een robot? En tot waar blijft het gezond? Ik denk zeker wel dat de kinderen zijn die daar die daar gevoelig voor zijn, ja

**
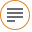
 9:9 ¶ 60 – 63 in Interview 7 12-03-2021.mp4--edited**

Dus het zouden dan kinderen met ADHD kunnen zijn of hoogbegaafde kinderen.

00:15:35 Interview participant 7: Met autisme.

00:15:36 MS: Autisme

00:15:36 Interview participant 7: Autistisch spectrum. Maarja het zal niet voor iedereen gelden, er zullen ook kinderen zijn in het autistisch spectrum doie het echt heel eng vinden of helemaal niks vinden omdat het toch ook onverwachte dingen geeft.Dus daar zou ik wel heel voorzichtig in zijn – om dat op die manier te te typeren.

**
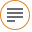
 10:17 ¶ 88 – 89 in Interview 8 31-03-2021--edited**

Je hebt het bij één kind gezien. Dat kind had dan al bepaalde stoornissen, of een speciale behoefte. Hoe uit zich dat bij bij dit specifieke kind?

00:21:25 Interview participant 8: Ja, dat elke keer als hij eerheen loopt, wil hij een knuffel geven, en als een andere groepje daar mee bezig is, dan is hij er ook wel eens moeilijk weg te slaan.

**
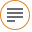
 10:23 ¶ 108 in Interview 8 31-03-2021--edited**

Mmm ja, dat kind met een hechtingsstoornis. Denk ik dan wel inderdaad, op die manier, wat we als doel hadden met die stappen voor een kind. Ik denk dat dat voor sommige kinderen wel heel veel baat zo we hebben, en ja, dat als zij er er heel veel mee gaan werken dat er dan ook weer meer hechting ontstaan.

**
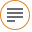
 11:18 ¶ 113 in Interview 9 2021-04-07.mp4--edited**

e introverte kinderen die daar die daar misschien dan wel een bepaalde uitlaatklep liet zien.

**
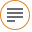
 11:19 ¶ 115 in Interview 9 2021-04-07.mp4--edited**

Ja, misschien ook wel kinderen die een veilige plek zoeken, veilige basis ehm ja, of of kinderen die, ja, ik denk gewoon even geen weerwoord kunnen hebben of geen tegenklank kunnen hebben, die daar die dat niet kunnen hendelen en dat die krijgen dat minder. Dus dan eh is dat ook weer veilig voor ze.

**
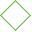
 ○ Attachement_too attached**

**23 Quotations:**

**
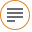
 2:34 ¶ 95 in Interview 2 12-02-2021.mp4--edited**

Nou, ik denk wat men erg het zorgwekkend zou vinden is wanneer en een kind zich volledig zo fixeren op deze robot en daarmee geen ruimte meer over te houden voor het sociale contact met de andere die groep. Ik denk dat dat moment zorgwekkend woord, want je wilt eigenlijk dat zon robot tot ingezet om niet alleen de sociale ontwikkeling van jezelf, maar met name door met je klasgenoten daaraan te werken. Daarmee bouwen op en de robot is misschien de manier waarop je kunt hebben over bijvoorbeeld emoties of gedrag. Maar uiteindelijk doe je dat samen met de je klasgenoten dus een leerling zo niet zon, robot moeten hebben op tafel. En dat is zijn begeleider. En daar is ie volledig op gefocust en de rest bestaan niet meer. Ik denk dat je dan eigenlijk een beetje een stap te ver gaat dat dan ja, dan loop je eigenlijk een beetje je doel voorbij.

**
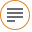
 2:35 ¶ 96 – 97 in Interview 2 12-02-2021.mp4--edited**

Ja, dus, als je groepen hebt, als die robot het centrale punt wordt en dat daardoor eigenlijk de de vriendschap relaties of de andere relaties met je klasgenootjes of met de menselijke docent daardoor in het gedrang komen.

00:34:14 Interview participant 2: Absoluut dan denk ik wel ja, ik denk het is meer een hulpmiddel het moet geen vervanging worden voor.

**
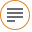
 2:52 ¶ 93 in Interview 2 12-02-2021.mp4--edited**

wanneer je het gevoel zou hebben dat deze robot bijvoorbeeld echt emoties zouden hebben, dat ze echt pijn zouden kunnen voelen. Maar ja, aan de andere kant kun je dat een kleuter kwalijk nemen wanneer hij bang is dat als als als olifant van de tafel afloopt, dat die misschien pijn heeft aan zn snuit en zou dat dan met een verlies van van realiteit gevoel zijn. Ik denk niet, dat is gewoon een jong, denk kind of hoe een jong kind denkt, dus dat vind ik heel lastig dan leg dan denk ik ook heel erg aan met wat voor leeftijd je te maken hebt.

**
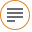
 3:29 ¶ 91 – 92 in Interview 1.mp4--edited**

MS: Maar je zegt niet dat ze dat later in haar leven problemen van krijgen.

00:30:10 Interview participant 1: Nee, nee.

**
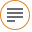
 3:53 ¶ 90 in Interview 1.mp4--edited**

als het echt een vorm van vriendschap gaat worden, zoals menselijke vriendschap als het een vorm van, want je zegt de gehechtheid dan misschien wel, maar ik tot nu toe is altijd een volwassene. Bij die dit begeleid in die dit bespreekt – tenminste – dat doe ik wel altijd wat voor apparaat is en dat het een robot. Deze emotie reet bij mij de baas zijn over en ik neem aan dat het. Ik ben nu die school, maar dat is om die weg is gegaan. Ja, dat ik ik ik denk dat altijd wel een mens bij is omdat dingen uit te leggen denkt. Tenminste, daar ga ik ervan uit, dat doe ik namelijk ook, maar niet. Iedereen is, denk ik zo maar ehm. Ik weet niet of dramatisch kan zijn. Ja, misschien ik ja heel steele mijn opa was die er ook in. Het was een broertje van een leerling, en die was is b, denk ofzo, andere en die robot stond dansen en die veel begint om en dat kinderen huilen en die schrok daarvan interim was gaan staan toen moesten lachen. Toen moest ie lachen, dacht ik: oh, dat is wel heel erg, dus ik ga niet met hele jonge kinderen. Dingen is nog niet te realiseren voor ze. Dus. Op die manier dacht ik: o ja, dan is die wel een beetje dramatisch voor het kind en een zoontje van mijn collega die ook weer naar huis genomen. Die was mijn pa voor dat ding, dat je stilstond eruit, want dat is niet eng. Maar als die anderhalf ook en toen ging niet meer aan de ene mn ouders die die was bij mn, bang voor zon, apparaat, maar dat was zon puur dat hij boog. Dus het begin was dat nog niet. Dat. Het apparaten is stiekem aan mn handen, kind of twee dus dat dat was wel heel bijzonder, maar echt ontrouw. Maar ja, ik denk niet dat hij nog wat meer naar huis nemen, want ik kan leveren misschien later het kind ouder is dan wel uitleggen. Het is.

**
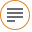
 5:19 ¶ 128 in Interview 3 17-02-2021**

Als kinderen helemaal alleen maar met de robot interactie willen, of negatieve ervaring krijgen als de robot niet aanweizg is, dan is het doel voorbij geschoten.

**
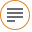
 5:23 ¶ 163 in Interview 3 17-02-2021**

Denk aan zelfde soort idee als game verslaving, helemaal online leven, en zo ook minder contact hebben met vrienden op school en daar ook de aansluiting gaan missen, minder uitngeodigd worden op verjaardagen etc. Maar op de manier waarop we nu robots inzetten zie ik weinig risico's.

**
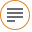
 6:24 ¶ 121 in Interview 4, 19-2-2021--edited**

: Ik denk dat je het in het onderwijs natuurlijk niet heel snel krijgt, alleen als je een robto altijd in de klas hebt, kinderen die die als te gehecht... dat zou er voor mij uit zien, dat ze op zich heel erg elke keer richten tot de robot. Dus dingen vertellen aan de robot eh zich vaker dingetjes vertellen aan de robot dan aan de juf dat, van dingen die ze mee hebben gemaakt, klein of dat ze weer binnenkomen of buitenspelen is wat er gebeurt, dat ze dan even langs langs de robot lopen om om hun hart te luchten, zo zou ik dat zien, terwijl ik denk, ja, dan ga je niet meer sociale interactie aan dat je met je klas of met je juf, maar dan probeer je dat bij een robot te halen

**
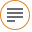
 6:25 ¶ 122 – 123 in Interview 4, 19-2-2021--edited**

Dus als het een negatief effect heeft op de sociale interactie met klasgenootjes of met met de leerkracht. Hoe zou je, denken dat ze op één meer gebruikgemaakt kan worden van die binding? Die die bijvoorbeeld die kleuters hebben bij de inzet van robots?

00:38:40 Interview participant 4: Ik denk dat je het misschien daar een soort van combi van zou kunnen maken als je als een kind heel erg gesloten is, dingen heel erg moeilijk vindt om op te pakken, dat je dan de robot zou kunnen inzetten om te kijken of of ze daardoor openen worden, bij bepaalde dingen – volgens mij is dat ook in tenminste, passen ze het ook toe psychologie bij kinderen in ziekenhuizen. Dat kun je natuurlijk ook toepassen in het onderwijs, ik weet alleen niet hoever je daarin moet gaan. Maar ik denk toch qua leren, dat het wel kan werken.

**
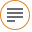
 6:26 ¶ 125 in Interview 4, 19-2-2021--edited**

Nou, je wil eigenlijk natuurlijk dat ze dat ze openstellen naar een leerkracht, dus je moet daar wel een soort een goeie overgang in maken dat je dat ze weet je het is mooi als even opstart door een robot, dat een kind weer gemotiveerd raakte, wat opener wordt, maar pak het wel weer op tijd te staan als dat ze dat ook naar jouw gaan worden [opener], zeg maar, zorgen dat er een mooie balans in is.

**
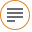
 7:12 ¶ 131 – 133 in Interview 5 3-3-2021.mp4--edited**

Kijk, als jjij dat zo zeggen, dan denk ik dat ze alleen nog maar dingen met een robot willen doen, maar dat dat zie ik hier niet gebeuren.

00:40:04 MS: Oké.

00:40:09 Interview participant 5: De robot is, de robot is leuk leuk, dus ze zijn gemotiveerd om met hem te werken, juist omdat het een ander werkvorm is, en ik vind juist ehm kinderen leren, als je altijd alles op dezelfde manier moet doen, dan stomp je een beetje af, maar door juist zo bij die coöperatieve werkvormen door het dan zo te doen en dan zo te doen en dan zo te doen en dan zo te doen wordt, het is, is het elke keer weer gemotiveerd, is elke keer weer leuk, door het andere jasje wordt het weer gaan ze weer, zijn ze weer geïnspireerd om ermee aan de gang te gaan. Dus het is gewoon ook een werkvorm om ermee aan de gang te... Dus ik denk niet dat, als het hier altijd is wordt hij weer gewoon.

**
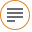
 7:14 ¶ 140 – 141 in Interview 5 3-3-2021.mp4--edited**

MS: Dus ik, ik hoor je, als ik het als ik het goed begrijp, zeg je nou er: zijn kinderen met problemen bijvoorbeeld die die bang zijn om voor een oordeel van een mens of of dat dan dat ze als gek worden beschouwd, bijvoorbeeld een andere problemen hebben. Die zouden het misschien wel gevoeliger kunnen zijn voor die binding met die robot en als dat, als je dat in het extreme trekt – en je zegt wat is dan te gehecht dat ze alleen nog maar willen interacteren met de robot.

00:43:30 Interview participant 5: Nouja jij hebt het over te gehecht natuurlijk, want dat was ook wel jouw vraag. Maar ik denk dat dat ja, dat dat ook wel een heel goed iets kan zijn, door ook nog een doel van de robot om deze in te gaan zetten. En en ja, misschien kan er dan bij helpen om wel je opdrachten te doen, dat je daarna wel durft omdat je hebt kunnen oefenen met iemand/robot die met dat niet oordeelt, dat je daarna ook de stap kan maken met iemand anders die wel een mens, een echt mens is maar dat is dan niet meer in jouw interview kopje van te gehegd.

**
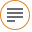
 8:11 ¶ 96 in Interview 6 12-03-2021.mp4--edited**

6: Dat dan de sociale component weg gaat en dat je alleen nog maar sociaal contact heb met een robot. dat je geen vriendjes meer hebt, maar je... en ja. ik denk dat dat het is.

**
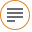
 8:13 ¶ 102 in Interview 6 12-03-2021.mp4--edited**

Als je daardoor, als je alleen de robto als vriend ziet en geen geen andere vriendjes hebt, als je tegen de robot praat alsof het een mens is, en als je de robot ziet waarheid. Als je hem zelf programmeert je natuurlijk nog heel goed in de hand wat hij wat kan en wat hij doet, maar op het moment dat die is geprogrammeerd, en je hebt eigenlijk alleen maar contact met die robot, ja dat lijkt me niet zo'n strak plan. Voor hoe ik het nu zie, het kan natuurlijk dat het allemaal mooi, anders wordt ontwikkeld. Maar dat is wel iets waar voor moet worden gewaakt.

**
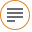
 8:14 ¶ 104 in Interview 6 12-03-2021.mp4--edited**

Nee, want dan is het nog een schoolse setting en is het eigenlijk een handlanger van de leerkracht. En ik denk dat dan het wordt gebruikt als ondersteuning alsof het een soort onderwijsassistent is. Maar op het moment dat de leerling denkt: oh, ik wil eigenlijk alleen nog maar naar de robot onderwijsassistent en niet meer naar de juf, want dat is wel lekker makkelijk of die, daar krijg je geen weerwoord van of, die zegt me niet wat ik moet doen, maar daar kan ik gewoon doen wat ik wil. Of daar voel ik me eigenlijk fijner bij, want dat is geen mens. Ja, dat is denk ik, wel je, dan worden ze wel problematisch.

**
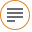
 9:16 ¶ 84 – 86 in Interview 7 12-03-2021.mp4--edited**

Nou dat dat kinderen echt verdrietig zijn als hij er een dagje niet is, bijvoorbeeld, of dat zo overstuur raken als je niet doet wat ze willen dat die doet, dan denk ik wel dat je te te gehecht raakt of of ja, zoals zoals kinderverliefdheid, weet je, dat ze zeggen van ik wil trouwen met een robot, dat soort dingen, dan moet je wel oppassen, dan ga je de verkeerde kant op. Ik weet niet of dat gaat gebeuren, maar dat zijn wel signalen van wachten even, misschien moeten we hier wel even wat mee gaan doen.

00:23:54 MS: Dus je zou het kunnen zien als als kinderen echt verdrietig zijn als ze de robot daar niet meer is, of mensen die er niet is als ze zeggen, ik wil met de robot trouwen. Zijn er nog andere dingen waarvan je zegt: nou, dat zou echt de een signaal zijn?

00:24:11 Interview participant 7: Als ze zeggen de robot echt mijn vriend en jij niet, weet je, dat dat ie ingezet wordt als een soort ruilmiddel. Kinderen kun anderen best wel onder druk zetten, maar dan ga je wel op glad ijs te bevinden.

**
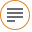
 10:19 ¶ 94 in Interview 8 31-03-2021--edited**

Nu, als ik heel duidelijk zegt, nu weggaan, dan luisteren hij wel. Maar als hij dat niet doet dan wordt het wel problematisch natuurlijk.

**
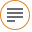
 10:20 ¶ 96 in Interview 8 31-03-2021--edited**

Misschien als elke les dat zou willen, of als er echt heel veel vraag naar is.

**
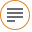
 10:21 ¶ 97 – 101 in Interview 8 31-03-2021--edited**

En waarom zou dat problematisch zijn?

00:23:16 Interview participant 8: Nou, omdat je ook op andere manieren moet leren.

00:23:24 MS: En waarom?

00:23:28 Interview participant 8: Het grootste gedeelte leer je uit het boek, en ja, je moet ook zelf het kunnen lezen en dan wordt je actiever denk ik.

00:23:39 MS: Oké, dus, als iemand zich te veel hecht aan een robot en hij heeft te veel contact of hij wil te veel contact of zij met die robot, dan zeg jij, dan wordt het een beetje gemakkelijk ofzo het leren een beetje. Dan gaan kinderen niet meer echt actief aan de slag.

**
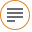
 11:14 ¶ 103 in Interview 9 2021-04-07.mp4--edited**

Als het niet zonder kan, denk ik: ja.

**
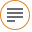
 11:15 ¶ 105 in Interview 9 2021-04-07.mp4--edited**

In in de zorg en heimwee misschien ook wel daarna of geobsedeerd erdoor ik denk dat het op die manier zich zou kunnen uiten

**
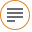
 11:16 ¶ 107 in Interview 9 2021-04-07.mp4--edited**

Dat alleen alles om daarom moet draaien en dat die overal in het gesprek ook bij betrokken wordt. Ik denk dat dat ja, het is een beetje mijn aanname natuurlijk, maar zo ziet ja, dan denk ik wel dat dat het niet zonder kan.

**
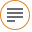
 11:17 ¶ 109 in Interview 9 2021-04-07.mp4--edited**

maar als ze echt zorgen maken en als het belemmeren het is in het dagelijks handelen, dan denk ik dat het, dat je dat er wel zorgen zijn

**
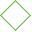
 ○ Attachment**

**19 Quotations:**

**
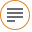
 2:9 ¶ 5 in Interview 2 12-02-2021.mp4--edited**

En ze vroegen ook meteen wanneer die we terug zou komen en iedereen wilde voor hun zorgen je merkte echt dat daar het zorg aspect heel erg naar boven kwam. Dus dat het eigenlijk een beetje een maatje was geworden. En hetzelfde was ook bij Alphamini, ze wisten ook heel goed, het is iets breekt basis. Fragieles, je moet er voorzichtig mee zijn, maar je doet wel wat hij zegt. En als jij iets vraagt dan reageert hij op wat je vraagt en als jij hem iets laat doen, dan doet hij dat hij dat ook en ze vonden dat. Ze bouwden er heel snel een band mee op en dat vond ik zo, zo grappig om te zien eigenlijk, dat en dat is natuurlijk ook wat je wil, maar dat is altijd de vraag. Hé. Gebeurt dat dan ook echt en hoelang duurt dat zou dat normaal duren, en ik merkte dat dat bij de jonge kinderen sowieso heel snel ging. Ik denk dat dat natuurlijk ook wel een beetje de kleuters het eigen van hetjonge kind is dat je natuurlijk heel erg is. Nou, open staat voor dat soort dingen je ben nieuwsgierig je accepteert wat sneller, maar ook bij groep vijf en je zou denken: zon, zon, paartse speelgoed Olifant spreekt dat zon, kind van groep vijf nog aan. Nou, ja, vonden ze geweldig, vonden ze top en zn konden het koppelen aan het speelgoed wat ze thuis hadden

**
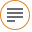
 2:26 ¶ 63 in Interview 2 12-02-2021.mp4--edited**

Ik denk het wel, denk ik zou zeggen waarom niet, en ik denk dat hetzelfde als ik naar mijzelf kijk als kind en naar het soort speelgoed dat onder andere bijna robots waren. Daar had ik ook genoeg binding mee en en zeker als een onderdeel wordt van de klas en van het onderwijs en het niet eens meer los van elkaar kunt zien. Dan denk ik dat die binding automatisch ontstaat.

**
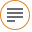
 2:27 ¶ 65 in Interview 2 12-02-2021.mp4--edited**

: Ja, absoluut, en dan zeker wel, hè, want we hebben ook met technieken gemerkt waar je niet zo snel binding mee hebt. Dus werken met sensoren en sratch robotjes nou ja, daar, daar heb je niet zo snel binding mee. Maar wanneer je een olifant neemt, of je neemt af een alpha mini die er ook echt uitziet als een robot pop. We hebben ook een dinosaurus die je kunt aaien, die net zo reageert als de olifant zien. Die zijn er een beetje uit als knuffels en ze laten gezichtsuitdrukkingen zien en ze reageren op wat jij doet, ik denk dat daar echt wel vooral dat ze daar wel echt wel binding mee krijgen, en dat heb ik ook met alpha mini gezien. En heb ik toen met die olifant ook gezien. Ze vonden het heel jammer, want ik moest een toen moest ik de olifant wegstoppen, weet ik nog en ik kan er toen in een doosje gezet nadat ik zn koekje waar die dan op reageerde als hij dat dan krijgt, en ik had hem in de berging gezet. Dus ikhaalde heel voorzichtig die doos te voorschijn heel veel zich het deksel eraf en ik had hem stiekem wel aangezet. Dus hij kwam eruit en toen pfrrrr je vonden het echt geweldig. Maar ja, toen moest ik hem wegstoppen en ik denk ik kan het niet uitzetten en in een doos pleuren en weg weet je wel, je moet wel de fantasie wel wel levend houden. Dus dan ging hij lekker slapen. En dan deden we een dekentje er over heen en zijn een koekje weg en deksel erop – en ze vonden dat super jammer – dat ie wegging ze hadden echt zoiets, hij mag gewoon hier staan en mag je lekker slapen. En dat was voor groep vijf. Dan zou je eigenlijk al denken. Ze zijn de kinderen lekker een beetje voorbij, maar dat kwam kan me helemaal terug naar boven.

**
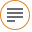
 2:28 ¶ 68 – 69 in Interview 2 12-02-2021.mp4--edited**

oe zie je dat kinderen zich verbonden voelen aan zon Robot?

00:25:51 Interview participant 2: Nou, ze kunnen er niet vanaf blijven ze willen alles proberen wat die kan. Ze gaan ook echt op ontdekkingstocht uit. Als ik me hier aai, wat gebeurt er dan? Ze willen hem allemaal vasthouden en ze zijn er heel voorzichtig mee, want ze weten ook heel goed dat ze er niet moeten laten vallen, want dan die of kapot gaan. Maar het kan ook zelfs pijn doen, misschien wel als er zelfs nog verder gaan. Dus ze zijn heel zorgvuldig ermee heel zorgzaam, maar ook heel nieuwsgierig en ook de reactie heel emotioneel dat ze moeten lachen als een bepaald geluid maakt, je ziet dan echt die ogen groot worden. Dus ja, dat zijn voor mij wel signalen dat ik denk. Nou ja, ze hebben echt wel, ja, ze hebben wel wat met deze robot.

**
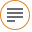
 3:26 ¶ 65 – 68 in Interview 1.mp4--edited**

, ik heb een paar vragen nog over de de emotionele binding met met de robot denk je dat kinderen een binding aan kunnen gaan met met een robots de emotionele binding zich verbonden kunnen voelen.

00:22:03 Interview participant 1: Heb ik gezien ja, ik moet de school waar ik werkte, daar dr. Ook als eerste hadden daar altijd leerlingen die mij al was naar de kleuters al bezig waren met robots, en die waren heel blij. Dat zijn heel blij om te zien, dus ze behandelen we echt als een een meisje, zeg maar in in zon is er geen dat echt? Het is gewoon een machine, maar voor hun was het echt een een een persona is. Een enkele kinderen hoor waren drie of vier kinderen habets, maar die waren echt helemaal niet op basis van oké. Dit is nu apparaatje als een knuffel ideeën, zeg maar was het op voor zon, speelgoed, muntje ofzo? Weet je wel dat? Ja, en dat is echt ja, die hadden echt aan, want daarmee ja.

00:22:53 MS: En.

00:22:54 Interview participant 1: Als een fout dat is ook zon, mooie mensen schrikken altijd jij fout. En dan denk ik, gaat welvarend zoals maar het is allemaal niet. Ja, ik heb dat helemaal niet, maar die kinderen, die zijn echt wel als je langer mee bezig zijn, nog jong. Al heel dan had ik echt zoiets van. Je moet echt helemaal ja echt verbonden, zeg maar ja.

**
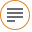
 5:21 ¶ 143 in Interview 3 17-02-2021**

ja dat denk ik wel

**
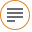
 5:22 ¶ 148 in Interview 3 17-02-2021**

Ja alleen dat vriendje

**
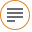
 6:22 ¶ 113 in Interview 4, 19-2-2021--edited**

Ik denk kleine kinderen, wel, ja, groep 8... nou ik denk het eigenlijk niet.

**
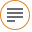
 6:23 ¶ 115 in Interview 4, 19-2-2021--edited**

Nou, bij de kleuters merk je het door vragen stellen die ze stellen, of ik er wel echt voor zorg zeg maar, dus dan krijg je echt wel vragen van of hij ook logeert en waar hij dan slaapt. Ik denk ja hij slaapt natuurlijk helemaal niet haha, dus door de vragen die door de kinderen op dat niveau gesteld worden, merk je dat je je echt moet moet uitleggen. jullie weten stiekem wel dat een machine is, maar het is ook echt niet dat dat, en dat die af en toe niest. Ja, dat is ook door iemand ingezet dat hij dat doet, en dat ie op een gegeven moment roept goh ik heb honger. Hij reageert natuurlijk ook echt dat kinderen dat ze denken: Oh, hij heeft ook honger, hij is ook verkouden, dus daar ga je oo gewoon op een leuke manier het grsprek mee aan. Een deel van de klueters heeft het helemaal door, maar sommigen zit daar toch wel een beetje dubbel in, Net zoals sommige gevoeliger zijn, denk ik, om veel met hun pop te praten en te doen, zullen sommigen ook met met de robot hebben.

**
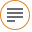
 7:9 ¶ 112 – 115 in Interview 5 3-3-2021.mp4--edited**

MS: Oké, ehm als we kijken naar de de mogelijk emotionele binding met een robot, dus de de de band die een kind kan voelen met een robot, wordt ook wel eens gesproken over soort vriend of bijna een vriendje.

00:36:13 Interview participant 5: Ja.

00:36:13 MS: Een huisdier of een of een of een ander soort wezen.

00:36:18 Interview participant 5: Nou ik merk wel dat als ik met de robot door de klas loop, of door de gangen loop dan reageren, kinderen: oo daar heb je Robo, weet je wel.

**
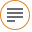
 7:10 ¶ 120 – 123 in Interview 5 3-3-2021.mp4--edited**

denk je dat kinderen zich verbonden kunnen voelen met die robot?

00:37:46 Interview participant 5: Dan moet ie toch wel mee bij ze in in de klas zijn, denk ik, echt wel kijk, nu zien ze hem af en toe toe en hij, kijk hij zou feitelijk ook dagelijks gebruikt kunnen worden, hè, zou ik hem op het kies bord zetten, want we hebben zon, digi, keuze, bord om te kiezen en je mag ook met Robo een spelletje doen, en ik zet elke dag een spelletje klaar, zou ik heus wel willen, maar die tijd ontbreekt mij dan dus ook gewoon, dat zou kunnen als je met twee personen voor een groep zou zijn. Dan zou je altijd een hoek met RObot kunnen doen, of een mat of weet ik niet wat, dan wordt het wel meer iets wat ook echt bij onze groep hoort, maar ja, dat hoort die niet. Daar komt ie gewoon te kort voor, Feitelijk zou zoiets best wel kunnen, maar die dingen zijn hartstikke duur, hè.

00:38:29 MS: Zeker zeker, er zijn ze komen niet voor niks heb je, heb je in je klas, wel als eens een soort verbondenheid tussen kind en robot gezien, dus dat ze dat ze hem inderdaad bij naam noemen of zeggen: dit is mijn vriendje of zich echt op op die manier verbonden voelen.

00:38:49 Interview participant 5: Nee, meer gewoon dat ik merk dat ze de verbinding hebben net als iemand anders die ook bij ons op schoolwoont , dus een net als een, kijk ze kennen heel veel meesters en juffen, ze kennen niet alle meesters en juffen maar bijna alle kinderen kennen wel Robo. Weet je zo, en dat ze hem zien van oo die, die hoort ook bij ons, maar niet zozeer in de groep, maar meer als die woont ook bij ons op school. Ja, dat is natuurlijk heel klein, maar dat komt omdat die dan toch te kort bij ons is.

**
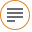
 7:11 ¶ 124 – 127 in Interview 5 3-3-2021.mp4--edited**

Maar ze zeggen wel, hij, hij woont op school.

00:39:19 Interview participant 5: ja hiij woont hier, ja.

00:39:22 MS: Is dat dan anders dan een een een handpop, bijvoorbeeld of een of een...

00:39:27 Interview participant 5: Nee, dat is, dat is hetzelfde denk ik, die woont ook bij ons. Die is natuurlijk ook vaak heel vaak stil, en die komt ineens tot leven als de juf hem pakt.

**
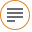
 8:10 ¶ 94 in Interview 6 12-03-2021.mp4--edited**

k hoop, ik weet niet of dat hoop is ik, ik ben bang van wel, maar ik zeg: ik ben bang omdat je nu natuurlijk nog wel eens schrikbarende dingen ziet, negatieve dingen laat ik het zo zeggen, en ik denk dat het, als je dat goed inzet, maar ik weet niet zo goed wat ik versta onder goed, maar als het goed inzet dat dat het dan ook goed kan. Maar uhm, ja je ziet nu natuurlijk wel dat kinderen zo vaak de computer als waarheid zien, met name jonge kinderen. Wat ze zien op de computer wat er gebeurt op de computer wat er nou ja bij wijze van spreken, hun virtuele vriend, via een online chat, is de waarheid, of in een in een online spel, want dat dat is bij wijze van spreken hun beste vriend in het extreme geval. Dus ja, dat is ook een soort van een robot, alleen zit die dan in een scherm en daar voelen kinderen zich zeker door aangetrokken. Ja. Alleen dat vind ik dan weer een wat midnere ontwikkeling, ten minste, er gebeuren gewoon nare dingen op het internet laat ik het zo zeggen.

**
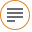
 8:12 ¶ 99 – 100 in Interview 6 12-03-2021.mp4--edited**

Oké, heb je dit ook meegemaakt in je eigen klassen?

00:32:26 Interview participant 6: Nee.

**
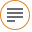
 9:14 ¶ 78 in Interview 7 12-03-2021.mp4--edited**

Nou ja, ongetwijfeld, maar dat zal niet voor alle kinderen gelden. Ik denk dat de meeste kinderen zich net zo verbonden voelen met de robot als dat ze zich met een ander ding verbonden kunnen voelen, als een spelcomputer of een boek of of een stuk speelgoed, of een fiets. Gebruiksvoorwerpen, hè dus. Ze kunnen dus wel van dingen houden. Ze kunnen wel daar heel gelukkig van worden of heel blij van worden of misschien ook wel heel verdrietig, maar echt echt, zoals houden van je vader of moeder of van je hond, dat is toch anders. Ik denk dat dat voor de meeste kinderen niet zo is, al zullen er wel kinderen zijn, maar dan ga je toch wel meer richting de kinderen die we net ook bij naar voren te komen, hè, kinderen die vallen binnen het autistisch spectrum, een beetje in die die trant, die gaan toch sowieso relaties al op een andere manier aan. Dus dat zou ik voor de robot kunnen gelden.

**
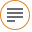
 10:16 ¶ 87 in Interview 8 31-03-2021--edited**

Ik heb er wel één die inderdaad wel eens eeen knuffel geeft, zat ik me net te bedenken, dus meer, die heeft ook een hechtingsstoornis. Ik weet niet of het daar iets te maken zou kunnen hebbenen – dus ik denk een paar wel, maar ik denk dat een grote groep het ook wel als een ICT middels ziet.

**
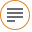
 10:18 ¶ 90 – 91 in Interview 8 31-03-2021--edited**

ké, dit heb je dus ook gezien in jouw klas. Zeg maar dat deze soort ja zo toch soort binding acht iets ontstaat, of dat iemand toch verbonden wil zijn met die robot. Bij één kinderen heb je daar bij meerdere gezien?

00:22:01 Interview participant 8: Nee, alleen bij hem.

**
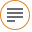
 11:12 ¶ 97 in Interview 9 2021-04-07.mp4--edited**

Dat vind ik heel moeilijk zeggen, dat weet ik niet, nee, dat durf ik geen antwoord op geven

**
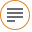
 11:13 ¶ 99 in Interview 9 2021-04-07.mp4--edited**

9: Ehm ja, omdat het stukje emotionele binding ze kijken dr echt naar uit, ze vinden het heel leuk. Ze, ze krijgen er ook wel zelfvertrouwen van. Maar of ze me echt dan denk ik ook van, nou zouden ze hem nou missen als ze thuis zijn, bij wijze van, of zouden ze zich zorgen maken: hoe het met hem gaat, als ze er niet bij zijn. Dat weet ik niet, dat heb ik nog niet gehoord of bekeken en daar heb ik ook echt geen zicht op gehad of ontdekt.

**
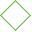
 ○ BEST: apply when needed**

**8 Quotations:**

**
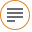
 5:40 ¶ 168 in Interview 3 17-02-2021**

iedere klas een robot en structureel inzetten

**
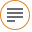
 5:43 ¶ 168 in Interview 3 17-02-2021**

Ook een duidelijke Why, niet zoals nu eerst de tech en daarna een doel zoeken

**
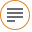
 5:44 ¶ 173 in Interview 3 17-02-2021**

Kinderen niet altijd laten werken met de robot, dat de robot niet de focus wordt van het leven van kinderen

**
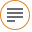
 8:36 ¶ 84 in Interview 6 12-03-2021.mp4--edited**

altijd naast iets zetten, als aanvulling, maar ik denk niet dat een robot de sociale skills van een kind zo kan beïnvloeden dat het kan zijn in plaats van, bijvoorbeeld een kanjer training die wordt gebruikt of een andere sociale vaardigheid methode

**
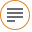
 9:21 ¶ 110 in Interview 7 12-03-2021.mp4--edited**

Niet inzetten om het inzetten

**
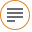
 9:34 ¶ 108 in Interview 7 12-03-2021.mp4--edited**

s. Wat ik zeg, boeken zet je ook in daar waar nodig is. En als je die niet nodig hebt, moet je ze vooral niet inzetten. Zorgt voor afwisseling. Kinderen houden ook van afwisseling, zeker in de huidige tijd. Kinderen vinden het prettig om afwisselend te leren en elk kind leert ook anders. Dus je moet ook zorgen dat je iets afwissend aanbiedt, dus ook niet een robot de hele dag elke dag aan, dat hoeft niet oké, wees, daar mag je ook wel terughoudend in zijn.

**
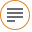
 10:45 ¶ 114 in Interview 8 31-03-2021--edited**

mm nee, ik denk dat je er wel goed voor moet maken dat het er niet altijd is, want dan, het moet wel een ook een speciaal ding zijn, denk ik, want als het te normaal wordt dan gaan kinderen er misschien ook geen zin meer in hebbeen, terwijl, het nu juist een boost geeft om te willen leren, denk ik.

**
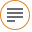
 10:46 ¶ 126 in Interview 8 31-03-2021--edited**

ik denk zelf ook wel in de gaten houden van de hoeveelheid tijd en de opdrachten die je waarbij je hem in gaat zetten, zeg maar, en robots zelf...

**
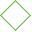
 ○ BEST: extra assistents**

**2 Quotations:**

**
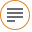
 6:31 ¶ 133 in Interview 4, 19-2-2021--edited**

Ik denk dat dat, wij hebben bijvoorbeeld extra hulp in de klas, de mensen die apart gaan zitten rtérs of andere die met kinderen aan de slag gaat, dat die eigenlijk die vaardigheden, ook gaan leren en dat die sneller een robot erbij pakken.

**
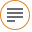
 7:29 ¶ 89 in Interview 5 3-3-2021.mp4--edited**

doordat we meer ook wat een klas assistenten krijgen wordt het die gaan, ben ik ook gedeeltelijk ook aan instrueren om in die robot aan de gang te gaan, want die kan ook met hun – die kunnen makkelijker met groepjes kinderen dingen doen, want het kan best wel met een groepje

**
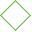
 ○ BEST: introduction by exp. teacher**

**1 Quotations:**

**
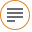
 6:57 ¶ 71 – 72 in Interview 4, 19-2-2021--edited**

Dat leerkrachten eerst een introductie krijgen of een les samen met een ervaren leerkracht met de robot en dan zelf aan de slag.

00:20:08 Interview participant 4: Eh ik denk dat dat ze heel erg helpt, omdat je eerst heel erg aan het stoeien bent. Hoe het allemaal in elkaar zit, en dat is eigenlijk gewoon, aangezien je zo veel vakken geeft op basisschool. Is het gewoon echt zonde van je tij

**
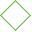
 ○ BEST: leerkracht aanwezig**

**8 Quotations:**

**
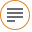
 2:58 ¶ 49 in Interview 2 12-02-2021.mp4--edited**

ls je het maar rustig introduceert en je laat ze kennismaken en je moet niet.... niet met zon, pop of met zn olifant in het gezicht douwe en zeggen dit is leuk. Kijk raak aan tof, nee, je moet ze gewoon. Dat ding moet goed neerzetten en laat ze zelf maar uitproberen en zelf die grenzen opzoeken. Dus ik denk denken: ja, je hebt altijd wel risico's met alles wat je doet en wat voor soort onderwijs je ook aanbiedt. Maar je moet gewoon oog houden op op je leerlingen en ze goed genoeg kennen.

**
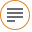
 3:61 ¶ 96 in Interview 1.mp4--edited**

die rol van de docent of de de volwassenen op de de de mens erbij vind ik wel heel belangrijk, niet altijd samen dat ze er samen zijn, maar dat er naderhand op daarvoor wel een gesprek plaatsvindt over wat er gaat of is gebeurd, of hoe het ging en wat het dan met het kind – want dat zijn de interessante aspecten – vind ik altijd wat ze ervan. En dan weet je ook of je een keer de minder gang moet laten gaan of juist niet nie

**
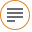
 5:20 ¶ 133 in Interview 3 17-02-2021**

Door een leraar die het in de gaten houd.

**
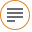
 5:39 ¶ 168 in Interview 3 17-02-2021**

In combinatie met een leerkracht of volwassenen

**
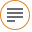
 10:42 ¶ 64 in Interview 8 31-03-2021--edited**

ou, zeker in het begin en op deze leeftijd zou ik eerst nog wel veel er bij zijn of in dezelfde ruimte inderdaad en ideaal zou zijn eigenlijk wel uit de klas, nu worden kindreen wel eens afgeleid, .... onhoorbaar, verbinding valt weg....

**
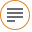
 10:43 ¶ 127 – 128 in Interview 8 31-03-2021--edited**

e leerkracht moet dat eigenlijk ook kunnen inschatten wanneer het te gehecht raakt. Die moet je moet als wij in de loop, in de loep blijven.

00:28:57 Interview participant 8: Ja, ik denk wel dat die meestal ook de kinderen ziet en dan weten het verstandig is om juist wel of niet in zetten.

**
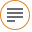
 10:44 ¶ 139 – 140 in Interview 8 31-03-2021--edited**

MS: Waarom is de aanwezigheid van die ouderen of een begeleider belangrijk?

00:31:25 Interview participant 8: Omdat ik denk dat, als wer wat fout gaat, kost het als ik wil helpen te veel tijd, tussen de klas en een robot te wisselen. Maar dat is zeker bij jonge kinderen, ik denk dat dat bij oudere dat het dan wel beter gaat.

**
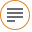
 11:38 ¶ 95 in Interview 9 2021-04-07.mp4--edited**

In deze setting niet, nee, misschien bij de kleuters kan het nog wel meerwaarde zijn, maar dat het zou zelfstandig moeten kunnen.

**
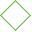
 ○ BEST: making contact with the child**

**2 Quotations:**

**
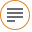
 9:35 ¶ 67 in Interview 7 12-03-2021.mp4--edited**

contact maakt met met kinderen, dus een robot zal er ook als, uit moeten zien, als iets waar je contact mee kan maken,

**
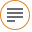
 9:38 ¶ 98 in Interview 7 12-03-2021.mp4--edited**

. Ja, dat zal vooral over vragen gaan, vragen laat stellen of zo, maar het doel blijft om kinderen met elkaar te laten communiceren. Dat moet eigenlijk al voorop staan.

**
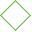
 ○ BEST: mindset other teachers/ management**

**3 Quotations:**

**
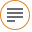
 6:58 ¶ 135 in Interview 4, 19-2-2021--edited**

Dus de drempels worden wel steeds kleiner, en ik denk door de ontwikkelingen omdat heel veel digitale is dat het ook steeds kleiner gaat worden, waardoor het steeds makkelijker gaat worden om dan de robot de klas in te schuiven.

**
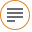
 7:28 ¶ 89 in Interview 5 3-3-2021.mp4--edited**

een team die acht je staat, de directie die achter je staat, een team die achter je staat en iemand die tijd heeft om aansturen

**
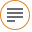
 11:37 ¶ 129 in Interview 9 2021-04-07.mp4--edited**

één verantwoordelijke, die wij dus nu hebben, maar ook constant levendig te houden, door te benoemen, maar ook in de team momenten aan te kaarten van wat is er gebeurd? Hoe kunnen we het aanscherpen? wat kunnen we nog meer? Ja, omdat gesprek vooral, ja, echt in je beleid te te borgen van de aanpak. Dat is belangrijk, maar het moet wel echt een drive zijn om het om het in te zetten, want ik zie de voordelen zeker dan alleen. het is gewoon wel arbeidsintensief nog, en ik denk dat het wel beter gaat, maar nu is het heel intensief.

**
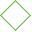
 ○ BEST: multiple classes working togetter**

**1 Quotations:**

**8:37 ¶ 84 in Interview 6 12-03-2021.mp4--edited**

En eh bijvoorbeeld met dat circuit ja, dan laat ik ook wel een kind van groep 5 samenwerkingen met een kind van groep, zeven, of groep acht, en als de kinderen van groep acht gaan helpen bij de kleuters of ze hebben ook wel geholpen met de NAo bijvoorbeel

**○ BEST: other room**

**1 Quotations:**

**5:42 ¶ 168 in Interview 3 17-02-2021**

ls je robots gaan inzetten is er ruimte voor nodig in de school

**○ BEST: partents involv.**

**1 Quotations:**

**5:41 ¶ 168 in Interview 3 17-02-2021**

ouders zijnnog niet toegerust om dit te waarborgen, school heeft een verantwoordelijkheid om ze goed voor te lichten.

**○ BEST: slow intro with children**

**4 Quotations:**

**2:57 ¶ 59 in Interview 2 12-02-2021.mp4--edited**

rustig introductie, dat kinderen snappen hoe zon robot werk, want dat is natuurlijk eigenlijk ook wel wat je wilt bereiken.

**2:58 ¶ 49 in Interview 2 12-02-2021.mp4--edited**

ls je het maar rustig introduceert en je laat ze kennismaken en je moet niet.... niet met zon, pop of met zn olifant in het gezicht douwe en zeggen dit is leuk. Kijk raak aan tof, nee, je moet ze gewoon. Dat ding moet goed neerzetten en laat ze zelf maar uitproberen en zelf die grenzen opzoeken. Dus ik denk denken: ja, je hebt altijd wel risico's met alles wat je doet en wat voor soort onderwijs je ook aanbiedt. Maar je moet gewoon oog houden op op je leerlingen en ze goed genoeg kennen.

**3:60 ¶ 94 in Interview 1.mp4--edited**

a, want ik al zei de rol van de mens, vind ik altijd heel belangrijk, nog steeds vooral bij jonge kinderen, om het te nuanceren voor hun op te laten begrijpen hoe het zit, omdat ze dat dat begrip nog niet hebben.

**6:59 ¶ 101 in Interview 4, 19-2-2021--edited**

Ik denk dat het goed is als je ze daar een keuze in hebben,

**○ BEST: small groups**

**4 Quotations:**

**9:36 ¶ 71 in Interview 7 12-03-2021.mp4--edited**

ou, ik vind dat het verstandig om in ieder geval nooit alleen met een robot te werken, maar altijd in een in een tweetal of een drietal.

**9:37 ¶ 71 in Interview 7 12-03-2021.mp4--edited**

Dan moet je namelijk sowieso al onderling met zn tweeën of met zn drieën communiceren Dan stimuleer je ook die communicatie. Dat, dat zou ik sowieso doen, en dat doen we ook altijd.

**10:41 ¶ 60 in Interview 8 31-03-2021--edited**

makkelijkste is en met kleine groepjes.

**11:36 ¶ 89 in Interview 9 2021-04-07.mp4--edited**

et ideaalplaatje dus wat mij betreft, de kinderen zijn in de groep aan het werk en er gaan kleine groepjes op enkelingen met de robot op de gang de activiteiten uitvoeren en zo zou ik het, zo zie ik het, en dan het liefst in de cyclus achterelkaar door.

**○ Bonding, buddy, friend, social actor**

**5 Quotations:**

**2:44 ¶ 5 in Interview 2 12-02-2021.mp4--edited**

En ze vroegen ook meteen wanneer die we terug zou komen en iedereen wilde voor hun zorgen je merkte echt dat daar het zorg aspect heel erg naar boven kwam. Dus dat het eigenlijk een beetje een maatje was geworden.

**2:45 ¶ 5 in Interview 2 12-02-2021.mp4--edited**

e bouwden er heel snel een band mee op en dat vond ik zo, zo grappig om te zien eigenlijk, dat en dat is natuurlijk ook wat je wil, maar dat is altijd de vraag. Hé. Gebeurt dat dan ook echt en hoelang duurt dat zou dat normaal duren, en ik merkte dat dat bij de jonge kinderen sowieso heel snel ging.

**5:30 ¶ 108 in Interview 3 17-02-2021**

Leerling gehad die zei dat de robot zijn beste vriend was,

**6:49 ¶ 109 in Interview 4, 19-2-2021--edited**

Ja, dan denk ik wel dat sommige kinderen gaan spontaan met een robot praten, of ermee spelen alsof het een vriendje is.

**8:24 ¶ 70 in Interview 6 12-03-2021.mp4--edited**

k kan me misschien voorstellen dat als je het gebruikt als een soort van maatje die je hebt voor programmeren, die misschien dingen terug zegt op bepaalde dingen. Nouja, als een soort van Siri, hoe moet ik reageren als, weetje een beetje zo, dat kan bijdragen

**○ Bonding, worries**

**4 Quotations:**

**2:50 ¶ 5 in Interview 2 12-02-2021.mp4--edited**

het zorg aspect heel erg naar boven kwam

**2:51 ¶ 69 in Interview 2 12-02-2021.mp4--edited**

Dus ze zijn heel zorgvuldig ermee heel zorgzaam,

**3:52 ¶ 66 in Interview 1.mp4--edited**

s ze behandelen we echt als een een meisje,

**6:50 ¶ 115 in Interview 4, 19-2-2021--edited**

krijg je echt wel vragen van of hij ook logeert en waar hij dan slaapt. Ik denk ja hij slaapt natuurlijk helemaal niet haha

**○ Challlenges**

**10 Quotations:**

**7:1 ¶ 42 in Interview 5 3-3-2021.mp4--edited**

Wel meer klassenassistenten laatste jaren krijgen wat meer ondersteuning en dat geeft wel input, want daardoor kunnen ze ook zeggen: Nou, die klassen assistent kan ook wel even met die robot aan de gang, want je moet hem pakken, je moet je er in verdiepen en je moet ook opruimen. Ja, het uurtje na schooltijd gaat echt heel snel, dus.

**7:17 ¶ 149 in Interview 5 3-3-2021.mp4--edited**

maar in de waan van de dag vergeten ze dat aan mij te vragen of hebben ze op dat moment niet even, je moet ik.... Dat is misschien het voordeel dat ik juist een kleuter leerkracht ben. Wij denken altijd ik doelen en we hebben geen methode, dus ik moet altijd zelf mijn materialen zoeken, hoe ik een bepaald doel wil bereiken bij kleuters, de leerkrachten hebben die hele methode die ze moeten volgen, daar staat alles al in idus die zijn niet getraind om steeds te denken hoe kan ik het nu anders doen, want we hebben gewoon die methode en dat is wel een beetje een.... Ze moeten dus eigenlijk als er een weekplanning maken voor de week daarna –denken he dit kan ik wel even met de robot doen, daar denken ze gewoon niet aan.

**7:18 ¶ 151 in Interview 5 3-3-2021.mp4--edited**

Ze vergeet gewoon, in de waan, ze vergeten het gewoon ook om daar rekening mee te houden, of aan mij te vragen. Ze weten wel dat ze tafels kunnen oefenen en maar de kinderen zijn makkelijker sneller gezet en die andere tafel oefening van bijvoorbeeld van Amprasoft dan dat ze even de robot pakken. Tegenwoordig heeft Robot in de klas, ook zon reken robot, dus hoef je niet een appje zelf te maken. Dus dat kan heel snel werken. Maar ja, ik, ik heb hem wel al aan groep vier ook laten zien dat het er is, maar het komt dan nog niet goed verder. Dat komt ook omdat de tijd, ook van mij, op een gegeven moment gelimiteerd is, en ik ze niet steeds, ze zien me al komen daar komt Margriet weer, want ik, we hebben ook een gewone leerlijn programmeren in de klas in de school. Dus ik heb de leerkrachten van groep vijf laatst ook al even Dash al weer onder hun neus gedrukt. Van weet u nog, hier hebben jullie Dash even, hoe werkt het ook allemaal weer, hebben jullie al een idee, hoe jullie dit dit jaar in kunnen zetten, zodat ze allemaal een keer mar Dash geprogrammeerd hebben, ja, dat vind ik ook heel belangrijk, want dat helpt ook als ze straks in groep zeven zitten en de NAO robot mogen programmeren.

**9:11 ¶ 69 in Interview 7 12-03-2021.mp4--edited**

Nou een robot is natuurlijk geen echt mens, en de kinderen moeten dat wel blijven beseffen. Hoe menselijke het wordt, het is nog steeds een apparaat, is nog steeds een machine, waar je geen menselijke menselijke relatie mee aan kan gaan. Een robot zal,zich als je vriend kunnen gedragen, maar het zal nooit echt je vrienden te zijn. Zeg maar zoals een mens dat zou zijn, of een hond hè, een hond kan wel, je vriend zijn natuurlijk, maar het blijft een apparaat. En als die stuk is, zeg maar, ja, dan dan doet ie het niet. Dus het zou een opstapje kunnen zijn om makkelijker contact te maken met andere mensen. Maar het zou het niet moeten vervangen.

**9:13 ¶ 73 – 75 in Interview 7 12-03-2021.mp4--edited**

Nou ik heb gesproken ook met studenten die dat hebben onderzocht.

00:20:28 Interview participant 7: Eigenlijk die zeggen van ja, dat dat systeem wat wat deze robot gebruikt dat staat eigenlijk wagenwijd open, dus dat wil zeggen als je de juiste ip adressen achterhaald., de jusite webadressen kun je in feite de camera gebruiken. Zou je in feite de microfoon kunnen beluisteren. Ja, dat is dat is via niet beveiligdde webpagina er zijn wel wat dingen, waarvan ik denk van ja.. dan moeten we wel goed op letten dat wel in orde is, en in feite is dat niet in orde, althans niet naar wat je de huidige maatstaven zou mogen verwachten. Dat gaat wel om kinderen, en dat is, dat ligt natuurlijk sowieso altijd gevoelig.

00:21:09 MS: Zeker, dat blijft ook een belangrijk aandachtspunt.

**9:22 ¶ 112 in Interview 7 12-03-2021.mp4--edited**

ou ja, dat stukje bezorgdheid van ouders, dat is wel iets wat wat wat altijd meegenomen moet worden bij bij de de aanschaf van de inzet van zo'n robot. Dat is iets wat je wat je nooit moet onderschatten, en ik blijf dat wel belangrijk vinden dat het op een veilige verantwoorde manier gebeurt dat als je een robot in gaat zitten dat je daar ook de ouders in ieder geval in meeneemt en van op de hoogte houdt van wat nou precies aan het doen zijn, ook omdat je met kinderen werkt. Daar zitten gewoon best wel een risico's aan. Risico zullen er altijd zijn. Net als dat je software gebruikt van externe leveranciers, ja, dan moet je eigenlijk een verwerkersovereenkomsten hebben, want ze verwerken data van je, nou ja van de robot hebben we dat dan niet, want in principe doet hij het meestee locaal, zeggen ze, maar ja, is dat wel zo? Hij is toch verbonden met internet. Nou, daar zitten wel risico's aan vast, daar mag je best wel voorzichtig mee zijn.

**10:13 ¶ 77 – 78 in Interview 8 31-03-2021--edited**

En wat zou volgens jou goede begeleiding zijn? Hoe zou dat eruitzien?

00:18:10 Interview participant 8: Ik denk wel iemand die ook bijvoorbeeld een beetje het programma weet, van stel hij stopt ergens zodat je dan weer snel weet hoe je 'm weer op moet starten. Of nou ja, ik heb standaard ingesteld, weet je het niet druk op linkervoet, dat ze dat soort dingetjes goed weten dat het proces vloeiend verloopt.

**10:31 ¶ 136 – 138 in Interview 8 31-03-2021--edited**

De leerkracht geeft les, en de robot staat bijvoorbeeld bij twee kinderen dat die vertelt, je moet nu een opdracht lezen of zo, dat die dan te afleidend is, dat is op dit moment denk ik de vorm om die de leerkracht meestal zouden kiezen.

00:30:56 MS: En dan de vorm: welke vorm zou dan jouw voorkeur hebben?

00:31:02 Interview participant 8: Ja, of echt met kleine groep is werken, en verschillende onderdelen wat wij nu doen, of echt uit de klas eventueel met ouders of assistenten..

**10:33 ¶ 144 – 147 in Interview 8 31-03-2021--edited**

gebruiksvriendelijker zijn en dat dat ook nog als dat gebruiksvriendelijk is, dat misschien leerkracht ook nog meer dan één willen verdiepen.

00:32:50 MS: En ga ik nu de portal van Robert in de klas te denken.

00:32:53 Interview participant 8: Ja, ja.

00:32:57 MS: Helder ik, ik heb me dr – zit gebruiksvriendelijk, pleit de applicatie.

**11:2 ¶ 55 in Interview 9 2021-04-07.mp4--edited**

: In het klaarzetten van de oefening. Het groepje kan er wel mee aan de slag, maar dat gaat best wel wat vooraf, in het programmeren. Kijk, zit het veel meer in je onderwijs verweven... Dan kun je ook zeggen: groep acht, die maakt iets voor groep één. Maar en dan is dat voor groep acht, voor de voor de meer intelligente groep die dat als uitdaging ziet, maar dat het zo ver is het gewoon niet. Maar ik zie de zeker echt goede kansen in. Ja.

**○ Fair of missing skills of knowledge**

**2 Quotations:**

**6:5 ¶ 12 in Interview 4, 19-2-2021--edited**

Het is meer de de techniek, dat ze bang zijn dat hen in de steek laat en dat mensen dan niet weten wat ze moeten doen en hij wordt wel eens te warm en daarom gaat stoppenof bij de kinderen lopen programmatjes vast. Ze zijn ook bang dat ze eh. Dat kinderen meerweten, dus dat je ze niet verder kan helpen voor hun gevoel dat ze echt alles ervan moeten weten van programmeren w omdat je kinderen in je hoofd moet helpen te ontwikkelen. Maar dat hoeft bij programeren eigenlijk niet, kinderen halen mij ook in, dat is gewoon zo. Die zijn zo snel. Ja, dat geeft ook helemaal niks, dan moet je gewoon mee gaan.

**6:6 ¶ 14 in Interview 4, 19-2-2021--edited**

dat ze nog niet het gevoel hebben dat ze het echt onder controle hebben. Ja, dan pakken ze toch niet snel.

**○ High-cost**

**1 Quotations:**

**6:7 ¶ 50 in Interview 4, 19-2-2021--edited**

: Daarvoor nemen we de Alpha mini's, omdat ze hetzelfde kunnen, maar ja... jaarlijkst kost de NAO ook al 1000 licentie kosten, dus het is gewoon een hele dure grap. Natuurlijk, en die Alpha mini's kan ik er dan vier hiervan aangeschafen, ipv één NAO.

**○ More susceptible: shy, ASD**

**13 Quotations:**

**3:54 ¶ 34 in Interview 1.mp4--edited**

schuchtere ook wat meer aparte kinderen. Die zijn heel erg getrokken door zo'n robot.

**3:55 ¶ 36 in Interview 1.mp4--edited**

die wat lager scoorden, of die juist sociaal wat zwakker zijn, want die gingen wel, die waren dan zo gedreven om met dat ding te werken dat ja, ik vind dat nog leuker dan een kind die het allemaal wel kom en eigenlijk heel snel een interesse verlore

**3:56 ¶ 40 in Interview 1.mp4--edited**

kinderen wat minder goed in een groep staan, nog minder sociaal vaardig zijn dan, en welk kinderen dat wel zijn. Dat heeft te maken met een bepaalde uiterlijke kenmerken. Wat sjofele loop uhm echt letterlijk uiterlijke kenmerken gewoon. Ze zijn wat sukkeliger zeg om met zo negatief te zeggen. Ze zijn soms ook heel snel boos omdat ze zich joh. Ja, ze moet voelen dat ze echt moeten opboksen tegen die andere kinderen. Ja, het zijn vaak een beetje de de de sulletjes van de klas die dat hebben end at kunnen meisjes of jongesn zijn zijn

**3:57 ¶ 42 in Interview 1.mp4--edited**

het zijn vaak de sulletjes, de wat de kleding is wat minder mooi, zeg maar of, past niet altijd even goed of van dei snottebellen hebben ze soms. Het zijn van die hele typerende dingen, dingetjes,,, het is heel zielig maar, ik moet het eigenlijk helemaal niet zeggen maar, het is wel zo.

**3:58 ¶ 112 in Interview 1.mp4--edited**

want daar zit dan, om het even bij sulletjes te houden, daar zitten de sulletjes van de school dus die gaan wel, en ze krijgen nu niet altijd van dat dat soort dingen, want dat is voor hun niveau niet haalbaar, zeg maar, om het maar even heel negatief te stellen, maar juist die kinderen hebben hier juist baat bij wat ze goeien op één of andere manier krijgen ze lichtjes in hun ogen, want ze zijn vaak natuurlijk onzeker over wat ze kunnen, niet kunnen, als ze van normaal onderwijs vanaf komen. Want ja, daar konden ze vanaflles niet. Dus ik denk dat hij daar gewoon veel beter thuis is. In het regulier onderwijs – ik heb ik – heb ik echt mijn twijfels er over. ja ook omdat ik niet zie dat heel veel scholen het omarm, nog steeds heel weinig scholen die het gebruiken.

**5:35 ¶ 98 in Interview 3 17-02-2021**

Sociaal zwakere kinderen, kinderen met ASD

**5:36 ¶ 113 in Interview 3 17-02-2021**

Sommige wat schuchtere kinderen kwamen erdoor meer los, en gingen ook dingen aan andere kinderen uitleggen

**6:56 ¶ 95 in Interview 4, 19-2-2021--edited**

die echt sociaal minder, die dat wel heel erg leuk vindt

**8:33 ¶ 76 in Interview 6 12-03-2021.mp4--edited**

e nerd in de klas, zonder aansluiting, die heel goed is in programmeren, heel heel zwartwit zegdt, en die kan de robot toch laten doen en zeggen, maar dan krijg hij [de leerling] wel wat meer aanzien. Dus dat ik misschien meer een gevolg van l zijn of dat. Oh, dat wil ik ook nou, laat maar met jou gaan een samenwerking want jij bent hier goed in, ... onverstaanbaar... in terwijl acht niet meer zo ervan, want het dan dat zou.

**8:34 ¶ 106 in Interview 6 12-03-2021.mp4--edited**

Dan denk ik moet name wel de kinderen die nou ja, wij bijvoorbeeld hebben we nu ook een jongen in in groep acht, game verslaafd geweest, blijf daar gevoelig voor, ja die type kinderen denk ik dat daar wel gevoeliger voor zijn, of juist de kinderen met autisme voor wie sociale contacten heel moeilijk zijn.

**9:33 ¶ 61 in Interview 7 12-03-2021.mp4--edited**

Met autisme.

**10:37 ¶ 56 in Interview 8 31-03-2021--edited**

et jongentje met autisme wat we denken te weten, want meestal is nog niet vastgesteld op deze leeftijd, maar die, ja moest ergens wel weer wennen, ofzo want het is even wat anders in de klas. Maar omdat het circuit ook nieuw was, moest die daar ook aan wennen.

**11:34 ¶ 113 in Interview 9 2021-04-07.mp4--edited**

introverte kinderen die daar die daar misschien dan wel een bepaalde uitlaatklep liet zien.

**○ More susceptible: ADHD**

**3 Quotations:**

**9:31 ¶ 57 in Interview 7 12-03-2021.mp4--edited**

kinderen die hoogsentitief zijn of die in ieder geval snel geprikkeld zijn door iets wat beweegt.

**9:32 ¶ 59 in Interview 7 12-03-2021.mp4--edited**

jewil natuurlijk niet in labels praten, maar de kinderen die een bepaalde aandachst behoefte hebben die kunnen niet helemaal in opgaan, hè dus. Als je kinderen met ADHD of gewoon kinderen die hoogbegaafdheid, die zouden hierdoor wel op een bepaalde manier , aangetrokken kunnen worden als het in hun straatje past en dan zijn er wel heel veel mogelijkheden om hiermee mee te werken.

**10:38 ¶ 56 in Interview 8 31-03-2021--edited**

. Maar hij reageerde er wel, goed op en nu, iemand ide ik verdenk van ADHD, die waren gewoon enthousiast.

**○ More susceptible: bonding issues**

**1 Quotations:**

**10:39 ¶ 108 in Interview 8 31-03-2021--edited**

hechtingsstoornis

**○ More susceptible: gamers**

**1 Quotations:**

**8:35 ¶ 106 in Interview 6 12-03-2021.mp4--edited**

game verslaafd gewees

**○ More susceptible: interested in STEM**

**3 Quotations:**

**5:38 ¶ 123 in Interview 3 17-02-2021**

kindderen die vanuit huis al bezig zijn met ICT

**6:55 ¶ 95 in Interview 4, 19-2-2021--edited**

kinderen die gewoon heel erg geïnteresseerd zijn in in robots en programeren,

**11:33 ¶ 79 in Interview 9 2021-04-07.mp4--edited**

die heeft thuis daar komt daar minder mee in aanraking. Een en ander kind die heel veel ermee in aanraking komt, de bedoel ik mee met met met computers, schermpjes dat soort dingen, die vindt het minder snel speciaal. Ehm ja, dus daar zie ik wel verschillen.

**○ More susceptible: less skilled**

**2 Quotations:**

**3:59 ¶ 36 in Interview 1.mp4--edited**

kind te pakken die wat lager scoorden

**5:37 ¶ 123 in Interview 3 17-02-2021**

De misschien wat minder vaardige kinderen

**○ More susceptible: young children**

**1 Quotations:**

**2:56 ¶ 5 in Interview 2 12-02-2021.mp4--edited**

ik merkte dat dat bij de jonge kinderen sowieso heel snel ging. Ik denk dat dat natuurlijk ook wel een beetje de kleuters het eigen van hetjonge kind is dat je natuurlijk heel erg is. Nou, open staat voor dat soort dingen je ben nieuwsgierig je accepteert wat sneller, maar ook bij groep vijf

**○ Reactions of other teachers, negative**

**22 Quotations:**

**2:18 ¶ 28 in Interview 2 12-02-2021.mp4--edited**

,ik denk wel wat je net ook al zelf aangaf dat veel leerkrachten het wel eens een drempel zullen aanvragen of zoiets in de klas te zetten. Zeker als je al een een ja docent bent die al een heel aantal jaren in het in het vak zit, en dan kom je ineens met zoiets. Ik denk wel, dat probeer ik aan mijn lessen ook altijd op te letten dat het zo ontworpen wordt dat de drempel zo laag mogelijk wordt, dat je alles zo duidelijk mogelijk eigenlijk al dat je alles over de leerkracht hebt gedaan en dat alleen maar de robot een lokaal of te zetten en hij heeft een lespakket voor zich en hij weet precies wat ie eruit kunnen halen hij of zij trouwens en hij weet precies welke programmeer stap hij zou kunnen zetten, maar ook genoeg ruimte bieden ze dat de leerkracht zelf ook nog dingen kan toevoegen. Kan aanpassen, ik denk dat ja dat dat eventueel de drempel zou kunnen verlagen, want ik kan me voorstellen dat... ik ik ik ben met robots gewend te werken, maar ik kan me voorstellen dat andere leerkrachten dat wat moeilijker vinden

**2:19 ¶ 30 in Interview 2 12-02-2021.mp4--edited**

Het is iets nieuws en ik denk dat heel veel leerkrachten ook niet weten hoe makkelijk het kan zijn. Want ik dacht ook wat ik in het begin ook al aangaf. Als je naar een jaar geleden had gezegd dat ik dit doen en zo en dan had ik ook gezegd, nee, ik, ik ben niet van de cijfers en programmeren. Laten we dat niet doen. Maar ik ben er ook achtergekomen makkelijker is. En ja, ik zeg altijd, ik zeg altijd op zeker op dit gebied. Als ik het kan, dan kan iedereen het, dus ik denk dat je vooral moet ervaren hoe simpel het is om het überhaupt te programmeren en hoe makkelijker hij aan te sluiten is op de bestaande lessen al, want ik denk dat heel veel leerkrachten bang zijn om te veel af te wijken van de lessen de lessen die er al zijn en de doelen die die moeten aantonen, want je wilt toch wel altijd ervoor zorgen dat je wel bepaalde leerdoelen wel gewoon aanbiedt in je lesaanbod. Een dan is het alleen een manier om te vinden waarin voor werk je dan zon, robot. Maar ja.

**2:20 ¶ 31 – 34 in Interview 2 12-02-2021.mp4--edited**

En zit dan in dat de de een soort angst waar je die je noemt om die leerdoelen niet te behalen of zit het meer in de gewoon de angst voor iets nieuws of voor extra werkdruk of?

00:16:04 Interview participant 2: Ja, ik denk dat het inderdaad snel als een extra werkdruk wordt ervaren.

00:16:08 MS: Oke.

00:16:09 Interview participant 2: Dat denk ik wel omdat je van je gevoel dan snel lijkt het alsof je iets nieuws moet gaan toepassen in je onderwijs, terwijl het eigenlijk niet altijd hoeft te zijn. En het is altijd: ja, leeft altijd extra werkdruk op als als je het gevoel dat er iets meer bijkomt. Dus ik denk dat veel leerkrachten dat zo kunnen ervaren. En dan wil ik niet de worden in de mond leggen van alle andere leerkrachten, er zijn er ook die er heel anders over denken.

**3:32 ¶ 106 in Interview 1.mp4--edited**

Positief negatief kan natuurlijk ook negatief is het het gewoon heel ingewikkeld apparaat is en dat er gewoon heel weinig is om met de robot te werken nog, ook wel doen de makers daar heel erg hun beste voor, en dat het gewoon best wel moeilijk is om te integreren. omdat het zo een gevoelig apparaat is dus bijvoorbeeld is– we hebben nou september ook geen robots kunnen gebruiken. Want we hadeddn nieuwe wifi en alles en de hele firewall die liet mn gewoon niet door. Dus we kunnen pas sinds deze week eindelijk weer de robots als noemaal gebruiken, maar dan ben je een half jaar verder omdat we er maar niet achterkwamen wat het probleem was, samen met met ontwikkelaar. Dus ja, dat is toch is dood zonde dat je zon apparaat niet kan gebruiken en dat soort manke dus het is vooral de infrastructuur, de praktische dingen van zn apparaat, het niet die niet durven werken met zn apparaat het is vaak maar 1 mens in de school die doen, dus het is heel erg leerkracht gebonden of ze pakken. Dus dat vind ik een en daardoor gaan leerkrachten zien wat voor effect heeft, maar om het dan zelf te gaan doen neeee, dat is nog een hele grote berg voor heel leerkrachten. Dus dat stukje zie ik wel en maar ze zien wel dan weer dat het dat kinderen goed reageren op dus ehm. Deels zie je nu wel de positieve effecten, maar ze zijn zijn er nog te bang zelf. Of nog niet kundig genoeg in hun ogen om het in te gaan zetten is ja, dat vind ik dan wel eens lastig, hoor daar lopen. Daardoor is er ook nog steeds in het hele ontdekken, fase waar we in zitten en komen we maarniet verder in het echt verdiepen.

**3:33 ¶ 109 – 110 in Interview 1.mp4--edited**

MS: Komt dat door door de technische obstakels die die je noemt, of zijn er ook andere obstakels die die daar een rol spelen.

00:35:14 Interview participant 1: Vooral technisch, oké, ja, dat is echt vooral technisch als ik, want op één school was een plan, maar dat is even in de koelkast gezet. Is het plan om leerlingen Robert experts te maken uit de bovenbouw die de onderbouw dan komen neerzetten aanzetten en met de kinderen aan de gang gaan. Daardoor haal je het technische aspecten weg bij de leerkreacht door dat andere kinderen dat gaan doen waardoor die ook die een bepaalde rol krijgen, en dat zorgt ervoor dat ze het eerder gaan pakken, want ze hoeven de technische dingen niet te doen, de kinderen gewoon leren met de robot bus is het makkelijker in in te passen in je dag dan wanneer je zelf dat dding moet pakken, aan moet zetten in moet loggen, aan moet zetten. Dat is echt veel tijd, kost dat, als het dan niet doet, moet je dat weer op zien te lossen. Ik deed het allemaal wel tussendoor , want ja, dat vond ik gewoon. Heel leuk, en je kon gewon meekijken wat er fout ging, vonden ze prachtig als juf iets niet lukt, dus dan kon ik ook laten zien dat ik ook fouten maak,om die dingen, en niet alles wist. Dus ja, ik vind het alleen maar leuke les niet alle leerkrachten denken zo dus, als je dat stuk dat technische weghaalt bij ze, dan zijn ze al eerder geneigd. Maar dan nog moet je heel goed kijken. Maar waneer gaan we inzetten dat, ja dat, dat vinden ze echt heel moeilijk. Dat is misschien nu vanwege de corona en technisch verplichten die je moet maken, dus die stap die we hebben gezet daarin, is het misschien anders. Misschien is het nu, als ik het nu zou doen dat ze anders naar technologie kijken, want ze moest er bij ons nu nu allemaal aan de macbook Dat wilde ze niet. En toen kwam ik Coronaen en nu zaten ze allemaal aan de macbook en gingen ze eindelijk in teams werken, dus ik hoop dat daardoor de technologie omarmd wordt, zodat dit ook beter gaat. Dus daar ben ik wel heel nieuwsschierig naar of idt misschien effect gaat heb. bij het inzetten van de robot – dus dat, maar dan ben ik nog niet, dat heb ik nog niet kunnen proberen.

**3:34 ¶ 111 – 114 in Interview 1.mp4--edited**

Als je zo de deze, technische uitdaging, ook andere uitdagingen overziet, denk je dan dat robots, sociaal robots, een een plek hebben in het primair onderwijs. En zo ja, welke plek dan.

00:37:12 Interview participant 1: Mijn eerste gevoel zegt nee, omdat ik... ten eerste zijn het hele kostbare dingen waar het basisonderwijs het geld niet, geen geld voor heeft, dus dat is een stuk financieel en ten tweede is het niet tot nu toe zie ik hem niet nog de opbrengst leveren die die zou kunnen leveren, die bijvoorbeeld een lesprogramma of een computerprogramma die ook of met de kinderne kan leveren. Ik weet wel dat hij ook op sociaal gebied veel meer bij kinderen teweegbrengt. Ook vanwege de emotioneel betrokkenheid die kinderen kinderen hebben, dus ik weet dat leren dieper gaat, dat weet ik, maar het wordt niet gevoerd in het onderwijs. Ik zie hem wel eerder bij het speciaal onderwijs, daar zie ik hem wel eerder omdat daar ten eerste een hele ook minded, meer open minded manier van lesgeven is, ehm en je daar heel specifiek ook op één ding kan gaan: ja, ik weet niet, ik denk niet dat hij daar beter past, ik weet niet waarom, maar ik denk dat hij daar gewoon beter tot tot zn recht komt en ik heb het niet gezien, maar een collega van mij bij een speciaalonderwijs over ons stichting geweest en daar werd hij echt heel goed ontvangen ehm, want daar zit dan, om het even bij sulletjes te houden, daar zitten de sulletjes van de school dus die gaan wel, en ze krijgen nu niet altijd van dat dat soort dingen, want dat is voor hun niveau niet haalbaar, zeg maar, om het maar even heel negatief te stellen, maar juist die kinderen hebben hier juist baat bij wat ze goeien op één of andere manier krijgen ze lichtjes in hun ogen, want ze zijn vaak natuurlijk onzeker over wat ze kunnen, niet kunnen, als ze van normaal onderwijs vanaf komen. Want ja, daar konden ze vanaflles niet. Dus ik denk dat hij daar gewoon veel beter thuis is. In het regulier onderwijs – ik heb ik – heb ik echt mijn twijfels er over. ja ook omdat ik niet zie dat heel veel scholen het omarm, nog steeds heel weinig scholen die het gebruiken.

00:39:05 MS: En hoe komt dat?

00:39:08 Interview participant 1: De meerwaarde wordt niet gevoelt. Het.. kijk ik, ik weet dit en jij weten de Elly weten het dat het een diepere laag van leren geeft, dat het door de emotie... dat het idee is dat het beter is dan een schermpje, alleen het is nog niet zo in het onderwijs, dat dat ze dat ook begrijpen en dat het effect, en we hebben nog geen effect kunnen meten ervan. Dat maakt dat het nog niet ingebed is, zeg maar in het onderwijs. Stel dat je wel kan zeggen, we hebben onderzoeken gedaan van een aantal jaren en en we weten gewoon dat het significant beter leert of anders leert, of voor die en die kinderen, maargoed daar zijn we nog mee bezig. Die resultaten heb je nog niet en dat maakt dat het nog niet met onderzoek bevestigd kan worden en daarvoor krijgen we geen directeuren mee of stichting mee of wat dan ook. Dus dat is gewoon heel lastig. Ja.

**3:37 ¶ 115 – 126 in Interview 1.mp4--edited**

n misschien een van de laatste vragen ehm de uitdagingen die je noemt en vooral op praktisch van aard.

00:40:10 Interview participant 1: Ja

00:40:12 MS: En misschien zelf bedrijfskundig dat je zegt de de kostenbatenanalyse is, is nog niet.

00:40:18 Interview participant 1: Wel een dingetje, ja, ja, zeker.

00:40:21 MS: Ze zijn er ook, of hoor je ook nog andere bezwaren. De min mogelijk morele bezwaren over de inzet van robots?

00:40:35 Interview participant 1: Ja, die was een tijdje terug, bij een interview van een ander onderzoek over de de privacy en zo ervan.

00:40:40 MS: Ja.

00:40:41 Interview participant 1: Want hij heeft een camera de NAo en waar stond dat dan de camera en wat gaat ermee gebeuren, dat is vooral dat privacy, moreel heb ik nog niet echt helemaal gehoord. Ik heb wel een leerling gehad in groep 7 of acht geloof, die wolde absoluut niet met de robot werken, ze moeten een werkstuk maken... Ja, maar ik ga niet mijn mijn woorden aan die robot geven. Het is toch mijn werkstuk, die had wel een bepaalde afecctie data zij haar werkstuk, dat zij haar tekst tekst in die robot moest gaan zetten, want ze moesten samen met de robot in de opdracht iets gaan vertellen, dat vond ze toch echt niet kunnen. Dus dat is de eerste keer dat ze....ik mijn woorden (onverstaanbaar) Nee, dat doe ik niet dus dat is echt gedaan, dat was de eerste keer, dat was. is dat een beetje een beetje een moreel kwestie denk je eingenlijk.

00:41:22 MS: Ja, het zou kunnen .

00:41:23 Interview participant 1: Dus dat was de eerste keer en verder hoor ik hem nog niet.

00:41:29 MS: Nee, niet van ouders, docenten, anderen, nee?

00:41:32 Interview participant 1: Nee, meer dat, ja ik heb wel wat ouderen die waarom moet ik met een robot werken? weet je. dat dat dat negatieve over. Waarom is het nodig, dat. meer van ik ken het niet dus waarom moet ik dit, zoiets. dus dat. Dat is een beetje dan de houding maar verder verder niet nog echt heel erg negatief ofzo. Of uit een morele kant, nee. Maar leerkrachten kunnen wel zo reageren, dat ze het ook niet echt nodig vinden. Maar dat was wat ik al zei die urgentie of het nut ervan zien ze nog niet altijd, dus ik heb ook al leerkrachten die dingen in de kast wilde hebben, want ze vonden het toch niet nodig, omdat kinderen te leren. Maar het is meer vanuit een principieel begrip ofzo, ik weet niet of dat moreel was.

**3:38 ¶ 130 in Interview 1.mp4--edited**

1: Die willen ook geen macbook hebben, die vinden een digiboard al heel imponerent, laat staan als we een robot in de klas gingen zetten. Dus dat ja, dat heb je ook die leerkrachten, nu misschien wat minder, maar ja dat, daar liep ik wel tegen aan soms ja.

**5:26 ¶ 188 in Interview 3 17-02-2021**

Mede leerkrachten vinden het verhoogd enthousiasteme van kinderen leuk, maar vinden het nog moeilijk technisch gezien om het in te zetten.

**6:3 ¶ 8 in Interview 4, 19-2-2021--edited**

En dan merk je toch dat de nieuwe groep acht leerkrachten meer even de drempel over moeten dat ze hem moeten pakken, dus het is een uitdaging om te zorgen dat programmeren niet bij 1n persoon hoort, maar dat het gewoon een onderdeel van eh van een lesprogramma gaat worden

**6:5 ¶ 12 in Interview 4, 19-2-2021--edited**

Het is meer de de techniek, dat ze bang zijn dat hen in de steek laat en dat mensen dan niet weten wat ze moeten doen en hij wordt wel eens te warm en daarom gaat stoppenof bij de kinderen lopen programmatjes vast. Ze zijn ook bang dat ze eh. Dat kinderen meerweten, dus dat je ze niet verder kan helpen voor hun gevoel dat ze echt alles ervan moeten weten van programmeren w omdat je kinderen in je hoofd moet helpen te ontwikkelen. Maar dat hoeft bij programeren eigenlijk niet, kinderen halen mij ook in, dat is gewoon zo. Die zijn zo snel. Ja, dat geeft ook helemaal niks, dan moet je gewoon mee gaan.

**6:6 ¶ 14 in Interview 4, 19-2-2021--edited**

dat ze nog niet het gevoel hebben dat ze het echt onder controle hebben. Ja, dan pakken ze toch niet snel.

**6:33 ¶ 135 in Interview 4, 19-2-2021--edited**

Maar ze zien natuurlijk ook ja, dat is gewoon weer nieuwe is. En hoe ga je precies toepassen, en hoe moet ik dat doen, het is weer iets te bij. Nouja het is niet iets erbij, je moet het in plaats van, gaan doen. Dingen anders doen is altijd in het onderwijs. Dus daar zit er nog wel wat uitdagingen om het allemaal geïntegreerd te krijgen, omdat ik zeg gewoon bijvoorbeeld je je moet voor aardrijkskunde die les laten vallen en je zet er gewoon de robot voor in. Dat zijn hick-ups waar je tegenaan loopt in het onderwijs, heel veel houden zich heel strak aan hun planning, wat ze dat jaar moeten doen. En daar moet ik gewoon tussen gaan komen. En sommigen vinden het heel vervelend als ICT dingetjes niet werken.

**6:35 ¶ 137 in Interview 4, 19-2-2021--edited**

Er bang voor zijn en tijd is altijd wel een soort dingetje. Je bent gewoon nooit klaar als leerkracht. Toen ik hier net komen werken toen zei mijn broer, oo lekker toch ook tegen mij toch elke dag gewoon om kwart over drie uit als de kinderen uit zijn. zo werkt dat gewoon niet. Dus je hebt altijd wat te doen. Het stopt gewoon nooit, en dat is de keuze wat je maakt als leerkracht, waar ga je tijd in investeren, ga ik nu investeren om mezelf te gaan verdiepen in programmeren of ga ik die tijd besteden aan eh. De rekencoördinator heeft ook heel veel leuke dingen, dat we met rekenen weer wat moeten gaan doen. Dus ga ik me daar in verdiepen, of... weet je... er worden altijd zo veel keuzes neergelegd. We zijn nu ook bezig op een school met mindset, met les over de hersenen gaan geven. Ja, super, maar dat is weer een keuze erbij, en daar merk je wel, er zijn elke keer nieuwe dingen. We hebben het directe instructie model gehad, dan heb je dit weer er komt elke keer vernieuwing en daardoor ja, moet je je plekje vel veroveren met programmeren.

**8:16 ¶ 110 in Interview 6 12-03-2021.mp4--edited**

de ene leerkracht hoe het allemaal heel spannend en vindt dat al bij wijze van spreken spannend om in te loggen op een laptop in plaats van op een vaste computer, dat type leerkracht met name hadden wat wat oudere leerkrachten, ook leerkrachten, die dachten o god dit kan ik echt niet, maar joh als je mij helpt, dan wil ik het wel proberen ehm of iemand anders. Of leerkrachten die zeggen van nou, ik vind het heel spannend, maar als de kinderen van groep acht komen, dan vind ik het leuk, en ook wel leerkrachten die zeggen ooo ik ben zo blij dat jij het doet, dan hoef ik het niet te doen

**8:18 ¶ 111 in Interview 6 12-03-2021.mp4--edited**

En hoor ik die zeggen dat de meeste bezwaren van van leerkrachten praktisch van aard zijn eigenlijk, als het gaat om het met meer missen van vaardigheden of de tijd er niet voor zien of voor voor hebben?

**8:19 ¶ 113 – 114 in Interview 6 12-03-2021.mp4--edited**

ren er nog andere bezwaar, morele bezwaren tegen de inzet van robots?

00:40:03 Interview participant 6: Nee, iedereen had wel zoiets van het hoort er nu gewoon bij. Dit is gewoon naar de nieuwe ontwikkelingen in het onderwijs, heb ik het gevoel, en er is vast een enkeling denken: Nou, het onderwijs is dat prima, zoals nu geven, maar op zich was de sfeer op school. Wel, oké, dit hoort erbij erbij en dit gaan doen.

**9:19 ¶ 105 – 106 in Interview 7 12-03-2021.mp4--edited**

De obstakels die je noemt, of de uitdagingen, klinken vooral praktisch van aard, dus de vooral de vaardigheden van de van de leerkrachten die ze moeten opdoen, zijn er ook nog andere bezwaren?

00:29:13 Interview participant 7: Ja, het stukje tijd wordt ook altijd genoemd, maar dat is eigenlijk alles wat in het onderwijs, er komt altijd de factor tijd kijken en daarvan voor tijd ook altijd de oplossing er is altijd kijken hoe je dingen kan integreren en weeg dingen af. Wat levert het op? Als de robot niks bijdraagt? Zet hem dan ook niet in hè, als als een iPad niks bijdraagt, zet je hem ook niet in. Maar als het wel iets bijdraagt, ja, wat wat kun je dan in je rekenonderwijs veranderen om die robot erbij te betrekken? Of als je iets met logo logaritmische wil doen, ja, dan kun je natuurlijk super die robot inzetten. Als je iets met taal wil stimuleren, als je gesprekken wil laten ontstaan tussen de leerlingen – ja, dan kun je die robot supergoed inzetten. Dus dan haal je een stukje van de robot in je les, om er weer iets mee te mee te doen, zodat je de robot weer in kan zetten.

**10:14 ¶ 82 in Interview 8 31-03-2021--edited**

Ja, dat programma bijvoorbeeld, dat zijn best wel wat stapjes want je moet doen, wil je het zelf helemaal een les maken. Ik denk dat ze dat nog niet onder de knie krijgen na – en je moet ook bijvoorbeeld eerst de robot aan internet zetten koppelen, dus er zijn best wel wat stappen wat denk ik dat het bij een paar belemmert

**10:15 ¶ 84 – 85 in Interview 8 31-03-2021--edited**

is dat dan de tijd die je erin moet steken die mist, of is het de kennis over die onderwerpen, of misschien angst vanwege de robots?

00:20:20 Interview participant 8: Eerst wel kennis en daarna ook wat tijd en misschien ook wel het nut ervan inzien.

**10:29 ¶ 130 in Interview 8 31-03-2021--edited**

Nou om er nu in te verdiepen, dat kost wel tijd, maar dan dat bedoel je niet helemaal nou, ik denk voor nu in de klas dus nog heel erg af leidend is als je het ja als je bijvoorbeeld klassikaal les geeft en één, twee kinderen zijn ermee bezig. Dat lukt nu nog niet, dus ik denk dat je wel echt goed moet kijken naar de vormen en dat dat nog een bezwaar is, van hoe moeten we het inzetten.

**11:25 ¶ 127 in Interview 9 2021-04-07.mp4--edited**

Nou ja, wat ik benoemde, dus het is best bewerkelijk, dat kost echt wel tijd, ook om het te integreren in je onderwijs, dat het verder het veel discipline dat het blijft gebeuren.

**○ Reactions of other teachers, positive**

**11 Quotations:**

**3:31 ¶ 106 in Interview 1.mp4--edited**

Ja, de de positieve dingen die ik hoor, is dat kinderen heel enthousiast terugkomen in de klas of is enthousiast zijn als het gaat gebeuren, is de. De betrokkenheid is heel hoog, dat vinden leraren heel leuk om te zien eh. Ik denk ook omdat het een hogere mate van betrokken is, dan in de klas gezien wordt op bepaalde momenten. Daar is kind natuurlijk vaak ook vaak betrokken, maar het enthousiasme straalt er of naar stroomt er zowat vanaf als kinderen terugkomen op met mij gaan werken. Dus dat vinden ze allemaal heel positief. Ook dat sommige kinderen, die dus niet zo vaardig zijn, of niet zo sterk opeens een een hele andere gezicht laten zien. Dus ze zien opeens andere kinderen soms ook. Dus dat is ook heel goed om voor leerkrachten die kant van de kinderen te zien is. Dat vind ik altijd leuk als dat gedaan als het als dat gelukt is, zeg maar niet van tevoren weten bij welke kinderen moet. Maar het is leuk als kinderen dat ook echt laten zien met zn gezicht hebben.

**5:26 ¶ 188 in Interview 3 17-02-2021**

Mede leerkrachten vinden het verhoogd enthousiasteme van kinderen leuk, maar vinden het nog moeilijk technisch gezien om het in te zetten.

**6:4 ¶ 12 in Interview 4, 19-2-2021--edited**

Op zich is de werking is de werking van een NAO robot wel makkelijker als je het één keer heb gedaan dan weet je echt hoe het werkt.

**6:32 ¶ 135 in Interview 4, 19-2-2021--edited**

er et algemeen zijn leerkrachten heel enthousiat,

**6:36 ¶ 139 in Interview 4, 19-2-2021--edited**

iedereen staat er echt wel. Eigenlijk zijn ze allemaal wel enthousiast.

**7:16 ¶ 149 in Interview 5 3-3-2021.mp4--edited**

Nou ja, kijk, ze zijn allemaal wel positief, ze willen ze allemaal wel dat de robot gebruiken in de klas

**8:17 ¶ 110 in Interview 6 12-03-2021.mp4--edited**

n ook wel leerkrachten, die, ja op zich wel voor open stonden en het wel wilde proberen, maar ik merkte ik wel bij de meeste leerkrachten dat het gevoel van, ik ben blij dat jij het doet, dan heof ik het niet te doen, dat dat daar een beetje overheerste, maar ik moet zeggen: de de sfeer op de op de Pieters op een gegeven moment wel zo dat ze dachten oke.

**9:18 ¶ 102 in Interview 7 12-03-2021.mp4--edited**

De positieve dingen: het is echter duidelijk de robot heel erg aanspreekt, ze vinden het echt heel erg, leerlingen vinden het heel interessant en leerkrachten die gaan er heel vaak dan echt wel mee, die die zijn van dan niet terughoudend. Die zien heus wel de leer potentie van een robot, hè, dus dat je daar echt wel een positief effect mee kan bereiken, maar de de dat stukje techniek. Dat is voor leerkrachten... daar ligt wel de uitdaging. Die proberen zoveel mogelijk weg te nemen. Wij roepen echt van joh. Als je bepaalde oefenmateriaal wil hebben, dan kunnen wij dat zorgen. Als wij met met jouw leerlingen met de robot moeten werken, dan gaan wij dat doen, hè dus. Wij proberen zo veel mogelijk uit handen te nemen en daarnaast zijn we een trainingsprogramma aan het opzetten om leerkracht het trainen, om leerlingen te trainen, die dan ook weer andere leerlingen kunnen trainen. Ja, echt zo veel mogelijk de weg effenen eigenlijk om het het zo makkelijk mogelijk te maken, want niemand heeft het geleerd tijdens de studie, hè, dat blijkt.. Maar dit is wel iets wat ja nu allemaal nog heel vers moderne nieuwe is, maar over over tien jaar over 20 jaar. Dan dan dan heb je hier onherroepelijk mee te maken – dat kan bijna niet anders, hè, zoals ooit alle digiborden de school in zijn gekomen en ooit alle alle computers de school in zijn gekomen. Dit wordt wel ergens een blijvertje.

**11:20 ¶ 117 in Interview 9 2021-04-07.mp4--edited**

We hebben ervoor gekozen om één leerkracht echt overal in te zetten, een Onderwijsassistent in de klas rondgaat dus ook echt een leerkracht te ontlasten en die geeft aan dat ze gewoon echt wel de betrokkenheid van kinderen ziet het is natuurlijk heel uitdagend, het is heel speciaal dat ze er mee mogen werken, dus daarin zie je echt wel betrokkenheid bij de kinderen en de motivatie om om het te doen, om om het goed te doen ook, dat is wat ziet.

**11:21 ¶ 119 in Interview 9 2021-04-07.mp4--edited**

Ja, gaaf dat zon ding bij ons op school is, dat vooral van nou, dit hebben wij en dat is wel heel, dat is echt een kans.

**11:22 ¶ 121 in Interview 9 2021-04-07.mp4--edited**

echt als een kans. Wij zijn ook wel een kleine school die de leerlingen nodig hebben, dus je zoekt ook wel daar in de kansen van Hé, wat kan ons nou steviger maken en een goed neerzetten? En ik zie hier in echt wel, de andere ook wel, de kansen van.

**○ SED: Ability to express oneself**

**6 Quotations:**

**3:48 ¶ 48 in Interview 1.mp4--edited**

: Ja, ik had één leerling kleuter destijds, die wilde totala niet leren, dat komt niet veel voor, maar die wilde echt, die had helemaal geen interesse in lezen of in letters of in rekeken of nog wat, maar die robot... dat was me toch echt wel hoor.

**6:48 ¶ 101 in Interview 4, 19-2-2021--edited**

, daar samen bijvoorbeeld, dat ze een hele veilige manier, waardoor we kinderen onbewust voor voor groepen staan, dat ze daar toch gaan aan wennen

**8:8 ¶ 85 – 86 in Interview 6 12-03-2021.mp4--edited**

MS: En, je noemde die robot kan misschien ook een negatief effect hebben op de sociale vaardigheden van kinderen of de sociale ontwikkeling. Hoe zou je de robot moeten inzetten zodat je dat voorkomt?

00:29:02 Interview participant 6: Nou, ik denk toch in die zin beperkt inzetten en dan bedoel ik niet niet dat je hem weinig aanbiedt, maar, weet je, dan een half uur per dag, of een uur op een dag of, en dat het niet alleen maar computers is, of dat als je hem inzet dat het dan is met een samenwerking opdracht.

**8:25 ¶ 74 in Interview 6 12-03-2021.mp4--edited**

Maar als je het samen laat doen, het licht, denk ik, net aan wat wat de opdracht is, is het: maak een presentatie, ja, dan is dit vind ik het een leuke aanvulling op, want je kan het samen met de robot doen, maar het is niet dat dat nou je heel veel socialer maakt. Het enige is wel dat je dan natuurlijk moet bedenken, oké, wat ik zeggen, en wat gaat de robot zeggen: wat geef ik soort van uit handen en kinderen gegevenhet wel uit handen aan de robot. Maar dat hebben ze zelf gedaan, zeg maar, het is natuurlijk het verdelen in, wat zeg ik? Wat zeg ji

**11:30 ¶ 63 in Interview 9 2021-04-07.mp4--edited**

Ddat vind ik wel een moeilijke vraag. Aan de ene kant, ja, zoals wat ik zei, dat jongetje dat dan ineens gaat praten omdat het laagdrempeliger is, dan zie ik een positieve factor, ehm. Dus wat dat betreft denk ik dat het zeker wel wat biedt. Ja, maar ik weet niet of dat voor elk kind zo is, dat durf ik niet te zeggen.

**11:32 ¶ 125 in Interview 9 2021-04-07.mp4--edited**

Ja, ik vind de veiligheid. Ik denk dat ze zich, kinderen die heel zeker zijn, dat die eerder daarmee aan de slag gaan dan, of het antwoord benoemen dan de twijfelaars die dan denk: ik: zeg maar niks, want straks het antwoord fout, en als je dan alleen maar zon kaartje voor hoeft te houden, dan is het natuurlijk al laagdrempeliger, dus dat dat zie ik wel een positief effect hebben.

**○ SED: Ability to express oneself - diff. topics**

**2 Quotations:**

**2:47 ¶ 36 in Interview 2 12-02-2021.mp4--edited**

Waarom zou je, doordat het juist iets is wat ehm, een beetje wat verderweg van je af staat en wat minder persoonlijk is, dus ik denk ik makkelijker op zon moment een moeilijkere dingen te bespreken en zeker op gebied van sociaal, emotioneel.

**6:44 ¶ 81 in Interview 4, 19-2-2021--edited**

en ik denk toch dat kinderen tegen een robot soms dingetjes, wel zouden durven zeggen of, omdat het toch anders is dan tegen de juf zeggen. Dit volgens mij ja, hij is wel iets wat wat kinderen tiggert

**○ SED: Ability to be patient and listen to others**

**7 Quotations:**

**3:50 ¶ 62 in Interview 1.mp4--edited**

Ik dan een pittige klas gedrag, dus dat vond ik helemaal leuk om te zien dat het bij mij dus ook niet helemaal lukte, maar dr. Robot lukte het dus wel, en dat was misschien ook binnen de nieuwigheid, want ik had een pagina gebruikt.

**7:22 ¶ 93 in Interview 5 3-3-2021.mp4--edited**

Als ze met de robot werk ehm, dan moeten ze ten eerste heel goed luisteren, dus de luistervaardigheden, dus wees stil als een andere wat zegt, want die robot, ja die kan reageren op wat zij zeggen, maar alleen op wat ik ingeprogrammeerd hebben, als zij er vanalles door heen tetteren dan dan hoort hij dat niet natuurlijk, dus sowieso geeft dat wel, en dat is ook een sociale vaardigheid, om goed naar elkaar luisteren en op je beurt wachten. Ik denk, dat is wel echt wel. De robot afdwingt.

**7:23 ¶ 95 in Interview 5 3-3-2021.mp4--edited**

Je moet dus ook de rust bewaren en je moet je impulsen ook bij je houden, want anders, je moet ook nog voorzichtig zijn, dus ik vind het ook een stukje, kinderen denderen overal bij wijze van spreken

**7:24 ¶ 101 in Interview 5 3-3-2021.mp4--edited**

dat ik gewoon met de juf werk en uiteindelijk al zou je dat in een school meer gaan doen, dan zou dat effect kunnen hebben op het moment dat ze ook met andere werken, want dat is natuurlijk de bedoeling, of met elkaar werken, ik moet goed luisteren, dus dat ja, dat is niet iets wat even hup even geregeld is. Dit is met meer sociale vaardigheden zo inderdaad het is echt een heel langdurig proces.

**7:25 ¶ 102 in Interview 5 3-3-2021.mp4--edited**

n zeg je de: ik heb in de klas wel gezien dat ze op elkaar moesten wachten, meer moeten samenwerken als zemet de robot bezig zijn

**7:26 ¶ 107 in Interview 5 3-3-2021.mp4--edited**

kinderen die het lastig vinden om op hun beurt wachten dat die in de knoop komen, want ze willen, hij moet eerst, de robot vraagt iets en soms herhaalt hij het twee keer dat, doe ik dan weer expres, maar na die ene vraag hebben zij het al gehoord en willen ze al reageren.

**7:27 ¶ 102 in Interview 5 3-3-2021.mp4--edited**

Dus als ik, als ik je goed horen, dan zeg je de: ik heb in de klas wel gezien dat ze op elkaar moesten wachten, meer moeten samenwerken als zemet de robot bezig zijn. Maar wil je dit effect van de robot zien op lange termijn, of moet wilde je robot echt effect hebben, dan moet je derobot op lange termijn inzetten om echt structureel effect te zien.

**○ SED: Ability to express oneself - emotions**

**1 Quotations:**

**2:46 ¶ 36 in Interview 2 12-02-2021.mp4--edited**

Zeker bij kleuters, een emotie en bepaalde bewoordingen van emotie. Ook woordenschat. Uitbreiding is kun je zo met zn spreken. Wat voor emotie zie je nu bij bij hem en kun je dat nadoen? En wanneer heb je zo een emotie? Dus kun je eigenlijk zeker kinderen die dat lastig vinden.

**○ SED: Afraid for the robot**

**3 Quotations:**

**2:23 ¶ 45 – 47 in Interview 2 12-02-2021.mp4--edited**

Mmm, wat dan ben je negatieve effecten, bedoel je dan ja, nou ja hetzelfde. Kijk in hetzelfde met zo'n, als ik dan weer zo'n voorbeeld van een pop neem, sommige kinderen zijn doodsbang voor die poppen.

00:20:41 MS: Mmm.

00:20:41 Interview participant 2: Die moet je daar niet gaan gebruiken, dus ik denk dat het wel heel goed is om een beeld te hebben waar staan waar je leerlingen en kunnen ze daarmee omgaan? Of werd wat meer introductie met het met het object in dit geval dan zo'n robot? Maar ik heb het eigenlijk zelf nog niet gezien en zeker met die olifant. Ik kan me niet voorstellen dat er kinderen zijn die ehm die die niet zouden toelaten omdat het een heel herkenbaar herkenbaar voorwerpen is omda thet heel erg lijkt op speelgoed.

**9:25 ¶ 42 in Interview 7 12-03-2021.mp4--edited**

Nou ik merk wel sommige kinderen vinden het wel echt eng, die vinden het spannend. Spannend, kan ook zijn op een van een leuke manier, maar zeker jongere kinderen vinden, dat vinden het echt wel eng. Ik heb bijvoorbeeld thuis neergezet, en mijn mijn oudste zoon die was, die was niet zo blij.

**9:26 ¶ 44 in Interview 7 12-03-2021.mp4--edited**

Toen hij aan ging vond het wel echt, eh kwam onverwacht, en het is dan toch ja, toch een afstandelijk ding, dus een relatie aangaan met een met een robot is, is is is toch voor jonge kinderen is dat heel lastig, want zij gaan daardoor, zij beseffen niet zo goed van hé, het is een machine, het is een ding, maar 't beweegt en het praten en dat geeft licht en geluid komt er natuurlijk uit. Die vinden dat wel, Daar moet je ze wel op voorbereiden. Oudere kinderen, die hebben juist wel dat heel interessantte, die duiken er eigenlijk echt bovenop, die vinden het zo, die ik ken uiteindelijk alleen van televisie van films, het is niet zo gewoon nog, dus die vinden dat wel heel interessant, van oooh hij kan praten, oooh hij kan dansen en ooh ik kan hem ook laten dansen, ja, dat dat dat trekt wel enorm aan.

**○ SED: Curiosity stimulation**

**2 Quotations:**

**2:48 ¶ 39 in Interview 2 12-02-2021.mp4--edited**

nieuwsgierig en dat hij toch wel zoiets van. Ik wil eigenlijk ook wat mee doen en dat was voor hem was dat een enorme stap om te overbruggen, want hij laat normaal zij zegt ik doe niet mee, dan doet ie niet mee en dan doet ie ook echt voor de rest van de dag niet mee.

**6:54 ¶ 123 in Interview 4, 19-2-2021--edited**

k denk dat je het misschien daar een soort van combi van zou kunnen maken als je als een kind heel erg gesloten is, dingen heel erg moeilijk vindt om op te pakken, dat je dan de robot zou kunnen inzetten om te kijken of of ze daardoor openen worden, bij bepaalde dingen – volgens mij is dat ook in tenminste, passen ze het ook toe psychologie bij kinderen in ziekenhuizen. Dat kun je natuurlijk ook toepassen in het onderwijs, ik weet alleen niet hoever je daarin moet gaan. Maar ik denk toch qua leren, dat het wel kan werken.

**○ SED: helping others**

**9 Quotations:**

**3:45 ¶ 46 in Interview 1.mp4--edited**

eg maar in hun tol rol en mmm en en soms is het ook lastig – vinden het moeilijk om aan andere kinderen uit te leggen, want het is sociaal, dat is bij hun natuurlijk een beetje lastig. Dat is dan weer een goede. En dan ga ik niet terug. Ja, maar hoe leg het uit en zorg ervoor dat je niet alles voordoet, of uitlegd, en laat zien en daarna zelf laten doen,. ik leer ze zo ook een beetje coach te zijn van andere kinderen. Dus dat groeit, dat groeit eigenlijk steeds meer. In die rol.

**3:49 ¶ 58 in Interview 1.mp4--edited**

En dan had één een tweetal lukte: het niet, is de voorganger die ging mee helpen uit zichzelf.

**5:29 ¶ 103 in Interview 3 17-02-2021**

motivation,en samenwerkingen skills

**8:23 ¶ 70 in Interview 6 12-03-2021.mp4--edited**

mdat we ze ook vaak in 2tallen laten programmeren, is dat sowieso samen gewoon sociaal bezig waren. Ehm dus dat was in die zin invloed op de sociale component van het samen bezig zijn

**8:26 ¶ 75 in Interview 6 12-03-2021.mp4--edited**

De, en maar daarnaast, en daarnaast kan het goed zijn voor de samenwerking van kinderen als ze samen met zon, robot interacteren met andere leerlingen.

**8:27 ¶ 75 in Interview 6 12-03-2021.mp4--edited**

Maar als een leerling alleen met die robot aan de slag gaat, kan het ook een negatief effect hebben op de sociale interactie met andere.

**8:28 ¶ 74 in Interview 6 12-03-2021.mp4--edited**

. Maar als je het samen laat doen, het licht, denk ik, net aan wat wat de opdracht is, is het: maak een presentatie, ja, dan is dit vind ik het een leuke aanvulling op, want je kan het samen met de robot doen, maar het is niet

**8:29 ¶ 74 in Interview 6 12-03-2021.mp4--edited**

we zitten allemaal steeds meer in die telefoon in de computer en zo'n robot moet worden geprogrammeerd, volgens de computer. En het is natuurlijk ook wel heel makkelijk om in jouw computer te zitten en te programmeren, o hij doet het, oké en ik ga door een programmeren als ze het alleen zouden doen. En nou, ja, we het dan een sociale robot is of een normale robot zeg, maar, dan kan dat juist soort de andere kant opgaan. Maar als je het samen laat doen, het licht, denk ik, net aan wat wat de opdracht is, is het: maak een presentatie, ja, dan is dit vind ik het een leuke aanvulling op, want je kan het samen met de robot doen, maar het is niet dat dat nou je heel veel socialer maakt

**9:27 ¶ 48 in Interview 7 12-03-2021.mp4--edited**

e hebben op een school waar kinderen, waar een aantal kinderen uit een andere de cultuur komen. Die praten moeilijk Nederlands die praten thuis geen Nederlands. Die vinden het lastig om in het openbaar te spreken en een robot helpt daarbij en en helpt dus bij hun eigen taalontwikkeling, waardoor ze ook makkelijker contact maken met leeftijdsgenoten.

**○ SED: Self-confidence**

**8 Quotations:**

**3:43 ¶ 36 in Interview 1.mp4--edited**

iet sociaal niet zon sterk stonden, kon ik daardoor juist op een podium zetten, want zij werd dan robot expert zeggen. Zij gingen juist andere kinderen weer leren of de juf helpen dus ze groeide daarvoor in hun hele zijn, zeg maar dat hun plekje in de klas veranderde daarvoor.

**6:43 ¶ 81 in Interview 4, 19-2-2021--edited**

Maar dan is zo robot is voor iedereen weer gelijk, omdat je sowieso het programmeren liggen sommige kinderen dan in één keer weer, dus die bloeien dan op, wat andere kinderen dan weer niet leuk vinden. Dus je ziet andere kinderen in één keer omhoog komen. Dat vind ik heel leuk aan programmeren, en je kan namelijk al op hele eenvoudige manier dingen te doen, dus iedereen heeft succes

**6:47 ¶ 97 in Interview 4, 19-2-2021--edited**

k denk dat voor het zelfvertrouwen, kinderen die inderdaad niet vor een groep durven te spreken, voor het zelfvertrouwen wel goed is, want uiteindelijk staan ze toch onbewust, voor de klas, naast die robot

**6:48 ¶ 101 in Interview 4, 19-2-2021--edited**

, daar samen bijvoorbeeld, dat ze een hele veilige manier, waardoor we kinderen onbewust voor voor groepen staan, dat ze daar toch gaan aan wennen

**9:27 ¶ 48 in Interview 7 12-03-2021.mp4--edited**

e hebben op een school waar kinderen, waar een aantal kinderen uit een andere de cultuur komen. Die praten moeilijk Nederlands die praten thuis geen Nederlands. Die vinden het lastig om in het openbaar te spreken en een robot helpt daarbij en en helpt dus bij hun eigen taalontwikkeling, waardoor ze ook makkelijker contact maken met leeftijdsgenoten.

**9:28 ¶ 48 in Interview 7 12-03-2021.mp4--edited**

Dat gaat in dit geval en om kinderen uit groep zeven of acht, maar kinderen voelen zich daardoor wel zelfverzekerd. Ja, en dat is natuurlijk het gaat uiteindelijk niet om de binding die een keer met een robot aangaat. Maar als die verbinding helpt om weer sociale relaties op te bouwen met leeftijdgenoten of met andere, dan dan is het natuurlijk een een mooie winst situatie.

**9:29 ¶ 48 – 50 in Interview 7 12-03-2021.mp4--edited**

Maar als die verbinding helpt om weer sociale relaties op te bouwen met leeftijdgenoten of met andere, dan dan is het natuurlijk een een mooie winst situatie.

00:12:16 MS: En dat dit heb je ook gezien in die klassen, dat ik.

00:12:19 Interview participant 7: Dat is wat we hebben meegemaakt, ja.

**11:31 ¶ 67 in Interview 9 2021-04-07.mp4--edited**

Nou, ik denk dat het wel kinderen de drempel over kan brengen en een drempel van hé, ik kan het toch, niemand anders heeft gezien, maar ik heb wel geoefend, een stukje zelfverzekerdheid kan geven, zelfvertrouwen, dus ik denk dat het daarin wel echt een een positieve invloed kan hebben.

**○ Social dev. impact general**

**49 Quotations:**

**2:9 ¶ 5 in Interview 2 12-02-2021.mp4--edited**

En ze vroegen ook meteen wanneer die we terug zou komen en iedereen wilde voor hun zorgen je merkte echt dat daar het zorg aspect heel erg naar boven kwam. Dus dat het eigenlijk een beetje een maatje was geworden. En hetzelfde was ook bij Alphamini, ze wisten ook heel goed, het is iets breekt basis. Fragieles, je moet er voorzichtig mee zijn, maar je doet wel wat hij zegt. En als jij iets vraagt dan reageert hij op wat je vraagt en als jij hem iets laat doen, dan doet hij dat hij dat ook en ze vonden dat. Ze bouwden er heel snel een band mee op en dat vond ik zo, zo grappig om te zien eigenlijk, dat en dat is natuurlijk ook wat je wil, maar dat is altijd de vraag. Hé. Gebeurt dat dan ook echt en hoelang duurt dat zou dat normaal duren, en ik merkte dat dat bij de jonge kinderen sowieso heel snel ging. Ik denk dat dat natuurlijk ook wel een beetje de kleuters het eigen van hetjonge kind is dat je natuurlijk heel erg is. Nou, open staat voor dat soort dingen je ben nieuwsgierig je accepteert wat sneller, maar ook bij groep vijf en je zou denken: zon, zon, paartse speelgoed Olifant spreekt dat zon, kind van groep vijf nog aan. Nou, ja, vonden ze geweldig, vonden ze top en zn konden het koppelen aan het speelgoed wat ze thuis hadden

**2:21 ¶ 36 – 39 in Interview 2 12-02-2021.mp4--edited**

Ja, ik denk een les die ik ontworpen heb voor kleuters met een alpha mini, bij alpha mini kan je bepaalde emotie slaat uitbeelden en dan kun je bijvoorbeeld heel goed met die kinderen in gesprek gaan. Zeker bij kleuters, een emotie en bepaalde bewoordingen van emotie. Ook woordenschat. Uitbreiding is kun je zo met zn spreken. Wat voor emotie zie je nu bij bij hem en kun je dat nadoen? En wanneer heb je zo een emotie? Dus kun je eigenlijk zeker kinderen die dat lastig vinden. Het kleuteronderwijs ook vaak, bijvoorbeeld poppen, ingezet om lastige onderwerpen te bespreken. Ja, ik denk dat zon robot absoluut daarvoor ook zou kunnen worden ingezet. Waarom zou je, doordat het juist iets is wat ehm, een beetje wat verderweg van je af staat en wat minder persoonlijk is, dus ik denk ik makkelijker op zon moment een moeilijkere dingen te bespreken en zeker op gebied van sociaal, emotioneel.

00:17:58 MS: Ja, en en wat voor wat voor invloed heeft die robot dan, denk je

00:18:04 Interview participant 2: mmm.

00:18:05 Interview participant 2: Nou, als ik kijk bij de kleuters, daar had ik een paar leerlingen die op sociaal emotioneel vlak wat ja, wat wat moeilijk. Ja, een beetje achterliep in ontwikkeling op dat gebied dus, een moeilijk een emotie konden uit niet konden konden gaan veranderen en de situaties dus. Er was met name één leerling en wij waren ook heel bang toen die robot daar kwam via bang. En je hebt altijd zoiets van oké hoe gaat zon leerling daar op reageren, want dat is iets nieuws, is onbekend en je merkt ook bij deze leerlingen dat die eigenlijk in het begin ook gewoon lekker op zn stoeltje bleef zitten en de rest bewoog lekker mee. Maar op een gegeven moment werd dat je toch wel nieuwsgierig en dat hij toch wel zoiets van. Ik wil eigenlijk ook wat mee doen en dat was voor hem was dat een enorme stap om te overbruggen, want hij laat normaal zij zegt ik doe niet mee, dan doet ie niet mee en dan doet ie ook echt voor de rest van de dag niet mee. En hij was mee aan het doen en dan liet ik hem ook programmeren. Ik dacht nou ja, dan kan hij ook een paar knopjes en indrukken. En toen zag hij reactie bij de robot en hij zag dat de rest van de kinderen mee deden. Dus dat dat is een voorbeeld van de leerlingen van ik zou zeggen: die heeft toen op sociaal emotioneel gebied echt een enorme brug over overgeslagen. Dus ja, dat is voor mij een praktisch geval geweest, want ik heb een terugzien.

**2:23 ¶ 45 – 47 in Interview 2 12-02-2021.mp4--edited**

Mmm, wat dan ben je negatieve effecten, bedoel je dan ja, nou ja hetzelfde. Kijk in hetzelfde met zo'n, als ik dan weer zo'n voorbeeld van een pop neem, sommige kinderen zijn doodsbang voor die poppen.

00:20:41 MS: Mmm.

00:20:41 Interview participant 2: Die moet je daar niet gaan gebruiken, dus ik denk dat het wel heel goed is om een beeld te hebben waar staan waar je leerlingen en kunnen ze daarmee omgaan? Of werd wat meer introductie met het met het object in dit geval dan zo'n robot? Maar ik heb het eigenlijk zelf nog niet gezien en zeker met die olifant. Ik kan me niet voorstellen dat er kinderen zijn die ehm die die niet zouden toelaten omdat het een heel herkenbaar herkenbaar voorwerpen is omda thet heel erg lijkt op speelgoed.

**2:24 ¶ 49 in Interview 2 12-02-2021.mp4--edited**

Ik denk dat ook zelfs kinderen die misschien wat sneller angstig zijn, bijvoorbeeld voor dit soort dingen dat ie zelfs nog weleens als je het maar rustig introduceert en je laat ze kennismaken en je moet niet.... niet met zon, pop of met zn olifant in het gezicht douwe en zeggen dit is leuk. Kijk raak aan tof, nee, je moet ze gewoon. Dat ding moet goed neerzetten en laat ze zelf maar uitproberen en zelf die grenzen opzoeken. Dus ik denk denken: ja, je hebt altijd wel risico's met alles wat je doet en wat voor soort onderwijs je ook aanbiedt. Maar je moet gewoon oog houden op op je leerlingen en ze goed genoeg kennen.

**3:15 ¶ 34 in Interview 1.mp4--edited**

Nou, wat ik vooral zie, is dat ehm een bepaalde type kind vaak de wat... nouja . schuchtere ook wat meer aparte kinderen. Die zijn heel erg getrokken door zo'n robot. En die gaan ook helemaal vol erin waardoor ze zoveel groeien zeg maar in hun zijn, dat het daardoor in de klas ook soms lekker loopt, want je kan ze triggeren of terugpakkenpakken van hé, maar met de robot ging het wel goed is. Dus het is niet zozeer sociale vaardigheden, maar meer hoe inzicht voelt, dus is dat ook een beetje wat je bedoeld, sociaal emotioneel.

**3:19 ¶ 44 in Interview 1.mp4--edited**

: Ja, ja, want ik pakte die juist die kinderen altijd om met de robot te gaan en die gingen, die waren helemaal gepakt door dat ding die kon ik niet wegkrijgen erbij ook en kon ik juist triggeren om te zeggen: ja, hartstikke leuk die robot, maar dan moet je kunnen rekenen. Want je moet wel snappen hoe die werkt. Oh, nou, en dan gingen ze wel. Het is daardoor kon ik die link leggen. tussen Waarom? Waarom we hier dingen leren, niet alleen omdat juf het zegt maar ook omdat je er echt helemaal doen. Natuurlijk in de toekomst is – en dat maakte wel dat ik het ja die robot eigenlijk kon gebruiken om hun tot leren te krijgen een aantal kinderen. Niet allemaal, maar het was wel vaak een motivator.

**3:20 ¶ 46 in Interview 1.mp4--edited**

Noe ja, het is een hele combi. Ik begin altijd dat ze die robot leren kennen en dat ze hem mogen programeren, En dat is al een heel ding, dat vinden ze natuurlijk fantastisch, dat zij de baas zijn van zo'n mooi apparaat, ze weten allemaal het is een heel heel duur apparaat en zij zijn dan de baas en dat stukje vinden dan al machtig mooi, en zodra ik vertel: Ja. Maar jij gaat alles leren hoe die robot werk, want jij gaat straks andere kinderen helpen dan hebben ze helemaal zoiets van oe... dan weet ik echt heel veel over robots, want dat willen ze dan ook vaak, het is een opbouw... ja ik, ik laat ze, zeg maar steeds meer groeien. Zeg maar in hun tol rol en mmm en en soms is het ook lastig – vinden het moeilijk om aan andere kinderen uit te leggen, want het is sociaal, dat is bij hun natuurlijk een beetje lastig. Dat is dan weer een goede. En dan ga ik niet terug. Ja, maar hoe leg het uit en zorg ervoor dat je niet alles voordoet, of uitlegd, en laat zien en daarna zelf laten doen,. ik leer ze zo ook een beetje coach te zijn van andere kinderen. Dus dat groeit, dat groeit eigenlijk steeds meer. In die rol.

**3:21 ¶ 48 in Interview 1.mp4--edited**

: Ja, ik had één leerling kleuter destijds, die wilde totala niet leren, dat komt niet veel voor, maar die wilde echt, die had helemaal geen interesse in lezen of in letters of in rekeken of nog wat, maar die robot... dat was me toch echt wel hoor. Zodra die robot was, dan deed ie alles. Dat was zo bijzonder die ging en alles doe wat hij moest doen, maar, maar niet bij mij, maar wel met een robot. die hadden ook een drone thuis en robot robotjes en hij was. Echt Heel visueel ingesteld ook en nog steeds kwam die niet tot leren die jongens nu 400, er zon vijf zelf zo, maar die was echt wel heel erg getrokken door de robot daar, waardoor hij tot leren kwam, want daar ging ie opeens wel de cijfers stellen, dat ik dacht hé je weet het dus wel, want ik kreeg ik, kreeg het er gewoon uit, bij mij klapte hij gewoon dicht, maar bij de robot dus wel. Wel, dus dat, dat was echt heel duidelijk verschil, dat heb ik niet heel veel meegemaakt. Op die manier. Dus ik kan niet, bij jonge kinderen – is het vaak dat ze ja ze individu identificeren zich met zon robto want hij is klein en ziet er schattig uit. Dus ze denken snel dat het een mensje is. Dus daarin kon ik dan wel weer gesprekjes hebben. Maar is het dan wel een mens ? Hoe bedoel je dat dan en hoe moet je met elkaar praten als de robot praat en hij gaat niet herhalen dus je moet luisteren? Ik kon ze eerder wat andere omgangsnormen leren dan dat ze zelf echt ja, zeg ik het goed. Ja, zoiets denk ik het lastig stond op te verwaaorden.

**3:23 ¶ 54 – 58 in Interview 1.mp4--edited**

nterview participant 1: Ik vind ik echt heel moeilijk, want dat is een stukje dus totaal niet echt een thuis ben, omdat ik heel erg in die kernvakken taal en rekenen, waarvoor ik veel gebruik. Ik weet of weet ik niet zo goed. Ik zou een stukje gebruik – ik eigenlijk nooit, omdat ik dat best wel lastig vindt, ook omdat het een apparaat is in. Ik kan niet van tevoren bedenken wat de kinderen gaan zeggen als ik het programma maken. Dat maakt het voor mij moeilijk om iets met sociale dingen te doen. Ik natuurlijk wel een lesje maken over hoe we met elkaar omgaan. Maar ja, ik denk ja, ik weet niet of heel moeilijk.

00:17:28 MS: En als je dan stel pakken en wat exactere vakken zoals rekenen bijvoorbeeld en dat je daar de robot voor voor zou inzetten in je klasse, dan is het misschien ook dr. Zie je misschien ook een effect op sociaal gedrag bij kinderen dat ze inderdaad Gods tegen mekaar zeggen, niet doorheen praten.

00:17:46 Interview participant 1: Ja.

00:17:47 MS: Denkt u dat dat een een belangrijk onderdeel is, maar de inzet van robots dat die kinderen die robot wel als sociaal zien.

00:17:56 Interview participant 1: Ja, want dat, dat is wel één van mijn doelen, altijd dat ze dus ik vertel je gaat oefenen, maar je moet ook leren. Hoe zon ding, hoe het werkt, wat je hoe je omgaat met elkaar in mee omgaat bij hogere roepen, als ik dat doe ik er natuurlijk een tijdje fijn als je dan vallen en heb ik hem ook gepakt. En toen heb ik wel uitgelegd van ja, maar dat hoeft niet allemaal met robots te gaan werken. Later moet wel begrijpen wat zon ding is en dat werkt en hoe dat dan in mekaar zit, of wat je dan moet we qua gedrag en dat als het niet lukt, dan moet je met elkaar wel, ja, dus dat soort dingen, dat leg ik wel uit en het wordt ook heel natuurlijk gedaan, vaak bellers toen ik zie wel als een als ik dan op tafel te oefenen gedaan. En dan had één een tweetal lukte: het niet, is de voorganger die ging mee helpen uit zichzelf. Wat in een normale situatie niet, hoe vaak ze niet heel vaak gebeurde, dus hij lokte wel bepaald sociaal contact uit. En ik had ook van joh. Je moet ook wel helpen, want ik ben met de rest van ik zoek het lekker met elkaar. Je kan elkaar helpen doen is dus dat dat ook wat de zeg, maar dat is gewoon dat ze dat deden, want dat was een mooie, het apparaat die daar stond en mochten dr. Mee werken. Dus dat is wel interessant.

**3:24 ¶ 59 – 60 in Interview 1.mp4--edited**

Was dat ook bij de bij alle groepen zo dat we vaak in een klein groepje met Robert zaten of één op één.

00:19:15 Interview participant 1: Één op één of twee, dan moet ik altijd doen. Bijna nooit ja, ik heb wel, maar ik heb hem wel. In het feit heb ik een aantal keer details geven. Gewoon om te kijken of dat werkte op. Dat lukte ook omdat ik gewoon niet kinderen dat mijn kennis laten maken en ehm had ik wel bepaalde rol in de klas gedaan. Is ene kind moest zorgen dat het kaartje laten zien, want dan moest die verder gaan met de volgende vraag, en de andere moesten we kijken of iedereen klaar met het woord schrijven, dus ik had wel bepaalde taken gegeven aan de kinderen, dus dat vond ik ook weer sociaal zit maar weer iets van van van de kinderen, en dat vonden ze wel heel erg leuk en werd ook heel goed ontvangen door de kinderen, dat ze dus de robot hadden. En ik heb toen heel erg. Ze observeren wat er gebeurt in de klas, omdat er ook voor mij niet is allemaal heel betrokken meer. Dan ben ik me, daar ben ik het niet gehad. Dat vond ik wel een voordeel.

**3:25 ¶ 61 – 64 in Interview 1.mp4--edited**

Ja, en meer betrokken wat, hoe zie je? Hoe zou ik dat zien? In mijn klas, dat kinderen meer betrokken zijn.

00:20:16 Interview participant 1: Ze doen heel goed wat ze van het wachtwoord, want het van tevoren gezegd als met leg je potlood nemen. We kan degene die mij niet continu zien dat je klaar bent en als ik dat zij is dat niet, want ja, je verwacht dat allemaal klaar zijn. Ik dan een pittige klas gedrag, dus dat vond ik helemaal leuk om te zien dat het bij mij dus ook niet helemaal lukte, maar dr. Robot lukte het dus wel, en dat was misschien ook binnen de nieuwigheid, want ik had een pagina gebruikt. Je ziet het als ik het langer zou doen, want het dan misschien andere sector hebben – dat weet ik dus niet. Dat denk ik wel. Dat is een nieuwigheid eraf ehm, maar ik zag wel heel duidelijk is hoe ze zich gedroeg als ze echt wat bijvoorbeeld vroeg. Ik moest wel dat het meisje dan in de gaten houden is is: is iedereen echt klaar en in de extra totdat neergelegd, want daar was ik wel bij nodig. Nog steeds maar het hielp wel, want die robot daar stond dat dat ze wel allemaal. Dat zag je dan ook.

00:21:10 MS: Luisteren, maar ook echt naar de robot.

00:21:13 Interview participant 1: Ja, ja, ze moesten we, want hij praat niet altijd duidelijk en en ik had er gelukkig wel een andere stem waardoor die wat rustiger sprak, is best wel fijn om het vrouwelijke stem. Ook dat zeiden ze, dat deed en ja, ze moesten we gewoon goed opletten, want hij zei ik maar één of twee keer per maand moesten ze opschrijft of ze moesten degene die dan moest aangeven, zeg je nog een keer doen. Dus moest echt elkaar daarin. Ja, ja, ze moest echt gaan kijken en luisteren. Dat was wel een hele leuke oefening.

**5:13 ¶ 98 – 103 in Interview 3 17-02-2021**

Sociaal zwakere kinderen, kinderen met ASD

Follow up questions, more detail

You could think of issues such as language development, learning, kind/unkind behavior, social interaction, friendschip.

Vooral motivation,en samenwerkingen skills

**5:14 ¶ 103 in Interview 3 17-02-2021**

Vooral motivation,en samenwerkingen skills

**5:15 ¶ 108 in Interview 3 17-02-2021**

Leerling gehad die zei dat de robot zijn beste vriend was, en die ook iedere keer enthousiast reageerde als de robots er was.

**5:19 ¶ 128 in Interview 3 17-02-2021**

Als kinderen helemaal alleen maar met de robot interactie willen, of negatieve ervaring krijgen als de robot niet aanweizg is, dan is het doel voorbij geschoten.

**5:28 ¶ 97 in Interview 3 17-02-2021**

Sociale vaardigheden, frienschap, communicatie, taalontwikkeling, vriendelijkgedrag.

**6:9 ¶ 81 in Interview 4, 19-2-2021--edited**

Maar dan is zo robot is voor iedereen weer gelijk, omdat je sowieso het programmeren liggen sommige kinderen dan in één keer weer, dus die bloeien dan op, wat andere kinderen dan weer niet leuk vinden. Dus je ziet andere kinderen in één keer omhoog komen. Dat vind ik heel leuk aan programmeren, en je kan namelijk al op hele eenvoudige manier dingen te doen, dus iedereen heeft succes, en ik denk toch dat kinderen tegen een robot soms dingetjes, wel zouden durven zeggen of, omdat het toch anders is dan tegen de juf zeggen. Dit volgens mij ja, hij is wel iets wat wat kinderen tiggert

**6:10 ¶ 83 in Interview 4, 19-2-2021--edited**

En, dus, ik denk dat dat wel werkt. En ik denk ook bijvoorbeeld, want ik wil het eigenlijk wel inzetten bij kinderen die bijvoorbeeld eh die we moeilijk aan het werk krijgen, voor rekenen, dat ze die sommetjes laten doen bij de robot, bijvoorbeeld omdat ze toch leuk, dat vinden ze toch leuk, dat willen ze eigenlijk toch wel graag de interactie met een robot.

**6:11 ¶ 85 in Interview 4, 19-2-2021--edited**

Nou, taalontwikkeling is wel een dingetjenatuurlijk, want hij spreekt dingen nog wel eens raar uit.

**6:14 ¶ 97 in Interview 4, 19-2-2021--edited**

Ik denk dat voor het zelfvertrouwen, kinderen die inderdaad niet vor een groep durven te spreken, voor het zelfvertrouwen wel goed is, want uiteindelijk staan ze toch onbewust, voor de klas, naast die robot

**6:17 ¶ 101 in Interview 4, 19-2-2021--edited**

, daar samen bijvoorbeeld, dat ze een hele veilige manier, waardoor we kinderen onbewust voor voor groepen staan, dat ze daar toch gaan aan wennen.

**6:19 ¶ 105 in Interview 4, 19-2-2021--edited**

oest ik denk dat je daar wel, ja kinderen het op een andere manier bereikt en bij de kleuters is het gewoon wel weer leuk, dus sociale interactie natuurlijk

**6:21 ¶ 109 in Interview 4, 19-2-2021--edited**

Ja, dan denk ik wel dat sommige kinderen gaan spontaan met een robot praten, of ermee spelen alsof het een vriendje is.

**6:42 ¶ 107 in Interview 4, 19-2-2021--edited**

Nou, inderdaad sociale ontwikkeling zou je langer in de klas moeten hebben, want dan zouden kinderen misschien wel bij wijze van praatjes met een robot gaan maken of zo, maar zo lang heb je inderdaad niet in een kleuterklas.

**7:4 ¶ 93 in Interview 5 3-3-2021.mp4--edited**

Als ze met de robot werk ehm, dan moeten ze ten eerste heel goed luisteren, dus de luistervaardigheden, dus wees stil als een andere wat zegt, want die robot, ja die kan reageren op wat zij zeggen, maar alleen op wat ik ingeprogrammeerd hebben, als zij er vanalles door heen tetteren dan dan hoort hij dat niet natuurlijk, dus sowieso geeft dat wel, en dat is ook een sociale vaardigheid, om goed naar elkaar luisteren en op je beurt wachten. Ik denk, dat is wel echt wel. De robot afdwingt.

**7:5 ¶ 95 – 97 in Interview 5 3-3-2021.mp4--edited**

: Echt over sociale vaardigheden, ja, kijk het het, het is ook. Ze moeten ook op dat moment in dat groepje om de beurt werken. Maar dat is in het geheel. Dus daar dat, dat vind ik ook wel heel belangrijk, want je kunt niet allemaal... Je moet dus ook de rust bewaren en je moet je impulsen ook bij je houden, want anders, je moet ook nog voorzichtig zijn, dus ik vind het ook een stukje, kinderen denderen overal bij wijze van spreken. maar dat kan hierbij echt niet. Dus ja, daar wordt echt ook wel wat van ze gevraagd. Hij stelt de vraag een keer, die moet je ook nog onthouden. Bij sommige dingen ook wel eens als het heel belangrijk is ook wat opties ingebouwd, als je het niet, als je het niet gehoord hebt, kun je nog een keer op zn hoofd aanraken, maar dat ja, dat, dat is niet zo intuïtief. Kijk als juf heb je gelijk door, o het is niet binnengekomen, maar die robot heeft dat niet door. Dus hoe is het eist, wat dat tegaat, wel andere vaardigheden dan als je met een leerkracht werkt, werkt, en je, ik denk dat bevordert wel op dat moment van dat ze echt moeten luisteren, echt samen moeten doen. Ja, want als juf ben je eigenlijk veel te goed, dat gevoel heeft de robot niet. Hij eist het een beetje van je, dat je rekening houdt met hem en dat je....

00:31:22 MS: Dus eigenlijk de limitaties van de robot: zorgen ervoor dat kinderen moeten samenwerken, beter moeten luisteren.

00:31:31 Interview participant 5: Goed om de beurt moeten doen.

**7:6 ¶ 101 in Interview 5 3-3-2021.mp4--edited**

Nou ja, ik ik zie dat gebeurd is, maar met alles wat je wat wat geleerd moet worden. Je moet jij, ze moeten gewoon vaak mee die robot werken, want dan mmm slijpt het ook een beetje in. En dan gaan ze ook ervaren. Als ik met die robot het werk, dan moet toch even anders werken als dat ik gewoon met de juf werk en uiteindelijk al zou je dat in een school meer gaan doen, dan zou dat effect kunnen hebben op het moment dat ze ook met andere werken, want dat is natuurlijk de bedoeling, of met elkaar werken, ik moet goed luisteren, dus dat ja, dat is niet iets wat even hup even geregeld is. Dit is met meer sociale vaardigheden zo inderdaad het is echt een heel langdurig proces.

**7:7 ¶ 102 – 105 in Interview 5 3-3-2021.mp4--edited**

Dus als ik, als ik je goed horen, dan zeg je de: ik heb in de klas wel gezien dat ze op elkaar moesten wachten, meer moeten samenwerken als zemet de robot bezig zijn. Maar wil je dit effect van de robot zien op lange termijn, of moet wilde je robot echt effect hebben, dan moet je derobot op lange termijn inzetten om echt structureel effect te zien.

00:33:08 Interview participant 5: Ja, want van één of twee keertje schiet dat natuurlijk ook niet op.

00:33:13 MS: Zijn er kinderen, denk je, die gevoeliger zijn voor een robot en het effect van de robot.

00:33:28 Interview participant 5: Ja, kijk, ik zei, net al, kinderen zijn heel erg gemotiveerd, ze willen die robot wel pleasen als het ware, want ze willen heel graag met die robot werken dus, want dat te gaat, staan ze anders in een opdracht, dan dat ze met een juf of een een stagiaire of een klassenassistent aan het werk zijn. Dus en de ene ja, de ene kind is daar gevoelig het gevoel dan het andere kind. Dus ik denk wel dat dat zo is. Ik heb er geen onderzoek gedaan, Tamara heeft el dat soort onderzoek onderzoek gedaan, weet ik, ja, ik denk wel dat dat daar een verschil in zit.

**7:8 ¶ 107 – 110 in Interview 5 3-3-2021.mp4--edited**

, vind ik wel een moeilijke vraag, nou je je merkt vooral dat de kinderen die het lastig vinden om op hun beurt wachten dat die in de knoop komen, want ze willen, hij moet eerst, de robot vraagt iets en soms herhaalt hij het twee keer dat, doe ik dan weer expres, maar na die ene vraag hebben zij het al gehoord en willen ze al reageren. En ik doe natuurlijk twee keer omdat ik weet van, je moet even goed de vraag luisteren, dus je moet ook even rust op jezelf zijn en het moet even binnen komen en sommige kinderen willen natuurlijk heel snel reageren, dat als dat een als dat na drie keer vraag zo is geweest, dan hebben ze wel iets van o ik moet toch even wachten, en vooral die ik in die heel erg impulsief zijn, die ja, dat triggert ze toch wel.

00:35:09 MS: Oké.

00:35:10 Interview participant 5: Snap je wat ik bedoel? Ik merk zelf namelijk ook dat ik de lesjes ging oefenen en ging kijken of mn appje goed was dat ik dacht Margriet rustig, hij moet het eerst twee keer vragen, ik wil natuurlijk snel even checken. Het kost gewoon tijd, dus ik moest echt in een soort ruststand staan van laat het je gebeuren. Het kost gewoon vijf minuten. Dit kan je niet sneller doen. Die robot gaat gewoon niet sneller.

00:35:39 MS: Nee, dus je je zegt die die in wat impulsieve kinderen die wat sneller gaan die, ja, die merken dat effect misschien sterker omdat ze sneller willen en eigenlijk tegen een soort limitatie van de robot aanloop of die robot verplicht om gewoon rustig.

**8:1 ¶ 70 in Interview 6 12-03-2021.mp4--edited**

Ja, lastige vraag: wat ik zie je bij de kinderen, omdat we ze ook vaak in 2tallen laten programmeren, is dat sowieso samen gewoon sociaal bezig waren. Ehm dus dat was in die zin invloed op de sociale component van het samen bezig zijn, ehm. Ik weet niet goed of door de robot zelf kinderen socialer worden, ook omdat ze in de setting dat ik gebruik natuurlijk zelf programmeren ehm. Ik kan me misschien voorstellen dat als je het gebruikt als een soort van maatje die je hebt voor programmeren, die misschien dingen terug zegt op bepaalde dingen. Nouja, als een soort van Siri, hoe moet ik reageren als, weetje een beetje zo, dat kan bijdragen. Maar ja, ik denk dat gewoon in een groep zijn, met de leerkracht, met groepsgenomten, dat kinderen daar socialer van worden.

**8:3 ¶ 74 in Interview 6 12-03-2021.mp4--edited**

u, ja, we het dan een sociale robot is of een normale robot zeg, maar, dan kan dat juist soort de andere kant opgaan. Maar als je het samen laat doen, het licht, denk ik, net aan wat wat de opdracht is, is het: maak een presentatie, ja, dan is dit vind ik het een leuke aanvulling op, want je kan het samen met de robot doen, maar het is niet dat dat nou je heel veel socialer maakt. Het enige is wel dat je dan natuurlijk moet bedenken, oké, wat ik zeggen, en wat gaat de robot zeggen: wat geef ik soort van uit handen en kinderen gegevenhet wel uit handen aan de robot. Maar dat hebben ze zelf gedaan, zeg maar, het is natuurlijk het verdelen in, wat zeg ik? Wat zeg jij? Dat is makkelijker, want uiteindelijk zeg je het in principe allemaal, alleen komt het uit de mond van de robot.

**8:4 ¶ 75 in Interview 6 12-03-2021.mp4--edited**

Dus je, ik hoor je zeggen: het kan kinderen die die ja sociale skills missen extra aandacht geven als je, want er is gewoon weinig tijd op scholen of is gelimiteerd heel veel tijd. De, en maar daarnaast, en daarnaast kan het goed zijn voor de samenwerking van kinderen als ze samen met zon, robot interacteren met andere leerlingen. Maar als een leerling alleen met die robot aan de slag gaat, kan het ook een negatief effect hebben op de sociale interactie met andere.

**8:8 ¶ 85 – 86 in Interview 6 12-03-2021.mp4--edited**

MS: En, je noemde die robot kan misschien ook een negatief effect hebben op de sociale vaardigheden van kinderen of de sociale ontwikkeling. Hoe zou je de robot moeten inzetten zodat je dat voorkomt?

00:29:02 Interview participant 6: Nou, ik denk toch in die zin beperkt inzetten en dan bedoel ik niet niet dat je hem weinig aanbiedt, maar, weet je, dan een half uur per dag, of een uur op een dag of, en dat het niet alleen maar computers is, of dat als je hem inzet dat het dan is met een samenwerking opdracht.

**9:3 ¶ 42 in Interview 7 12-03-2021.mp4--edited**

Nou ik merk wel sommige kinderen vinden het wel echt eng, die vinden het spannend. Spannend, kan ook zijn op een van een leuke manier, maar zeker jongere kinderen vinden, dat vinden het echt wel eng. Ik heb bijvoorbeeld thuis neergezet, en mijn mijn oudste zoon die was, die was niet zo bli

**9:4 ¶ 44 in Interview 7 12-03-2021.mp4--edited**

: Toen hij aan ging vond het wel echt, eh kwam onverwacht, en het is dan toch ja, toch een afstandelijk ding, dus een relatie aangaan met een met een robot is, is is is toch voor jonge kinderen is dat heel lastig, want zij gaan daardoor, zij beseffen niet zo goed van hé, het is een machine, het is een ding, maar 't beweegt en het praten en dat geeft licht en geluid komt er natuurlijk uit. Die vinden dat wel, Daar moet je ze wel op voorbereiden. Oudere kinderen, die hebben juist wel dat heel interessantte, die duiken er eigenlijk echt bovenop, die vinden het zo, die ik ken uiteindelijk alleen van televisie van films, het is niet zo gewoon nog, dus die vinden dat wel heel interessant, van oooh hij kan praten, oooh hij kan dansen en ooh ik kan hem ook laten dansen, ja, dat dat dat trekt wel enorm aan.

**9:6 ¶ 47 – 48 in Interview 7 12-03-2021.mp4--edited**

En de de het effect op sociale ontwikkeling en dan kun je ook denken aan, sociaal sociale interactie met andere vriendjes met met menselijke vriendjes zeg maar of vriendelijk of onvriendelijk gedrag of taalontwikkeling, denk je dat daar een robot effect kan hebben?

00:11:26 Interview participant 7: Nou, absoluut: we hebben op een school waar kinderen, waar een aantal kinderen uit een andere de cultuur komen. Die praten moeilijk Nederlands die praten thuis geen Nederlands. Die vinden het lastig om in het openbaar te spreken en een robot helpt daarbij en en helpt dus bij hun eigen taalontwikkeling, waardoor ze ook makkelijker contact maken met leeftijdsgenoten. Dat is wel wat we gezien hebben, dus dat heeft absoluut absoluut effect. Dat gaat in dit geval en om kinderen uit groep zeven of acht, maar kinderen voelen zich daardoor wel zelfverzekerd. Ja, en dat is natuurlijk het gaat uiteindelijk niet om de binding die een keer met een robot aangaat. Maar als die verbinding helpt om weer sociale relaties op te bouwen met leeftijdgenoten of met andere, dan dan is het natuurlijk een een mooie winst situatie.

**9:7 ¶ 49 – 50 in Interview 7 12-03-2021.mp4--edited**

S: En dat dit heb je ook gezien in die klassen, dat ik.

00:12:19 Interview participant 7: Dat is wat we hebben meegemaakt, ja.

**10:2 ¶ 40 in Interview 8 31-03-2021--edited**

e hebt wel direct feedback, dus daar kwam dan denk je dan goed gevoel geven dat je het goed doet, zeg maar, met sociale....

**10:3 ¶ 44 in Interview 8 31-03-2021--edited**

Ze willen we wel echt praten met de robot maar ze merken ook wel dat die dat niet allemaal kan, maar je merkt inderdaad wel, sommige zwaaien nog, die denk o leuk, maar of ze echt vriendschappelijker naar elkaar zijn. Dat zou ik niet weten. Dat zie ik niet per se, nee.

**10:4 ¶ 45 – 46 in Interview 8 31-03-2021--edited**

ké, heb je bijvoorbeeld wel eens gezien dat kinderen gezegd hebben: de robot is een vriendje, of heb je het idee dat ze het ervaren als een als een levend iets?

00:08:03 Interview participant 8: ja een paar wel denk ik, ja, want die willen echt wel dat hij wat terug zegd als ze wat vragen, ik denk dat vooral de oudste wel door hebben, hij heeft een programma en dat doet ie, maar de jongste dat dat nog wel denken ja.

**10:5 ¶ 48 in Interview 8 31-03-2021--edited**

: Ik zie vooral heel veel enthousiasme. Ik denk dat dat het is, en dat praten zie ik ook wel, maar of ze het daardoor anders doen, dat ik ik niet, nee.

**10:6 ¶ 50 – 52 in Interview 8 31-03-2021--edited**

Ja, ook wel van: Hoi robot en dat soort dingen.

00:08:59 MS: Oké.

00:09:00 Interview participant 8: "Leuk dat je weer bent."Ja, ik weet niet precies wat ze zeggen, omdat ik zelf ook met een groepje bezig bent is het ook wel eens lastig.

**10:12 ¶ 76 in Interview 8 31-03-2021--edited**

Nou, ik denk wel dat hij lange woorden maakt hij nu, daar hapert hij wel eens in, dat is niet helemaal vloeiend. Ik denk dat als dat verbeterd kan worden, dat weet ik niet hoor, dat dat dan voor de de taal dat wel goed gaat. Dat daar geen achterstanden bij komen, en dat ruzie maken, ik denk, als je goede kleine groepjes, en een goede begeleiding, en dat het allemaal vloeiend werkt, als die voorwaarden er zijn dat het dan wel goed moet gaan.

**10:22 ¶ 104 – 106 in Interview 8 31-03-2021--edited**

Je had zo straks, maar dat is net iets anders vraag, maar ook de ruzie van als ze de robot hebben, nouja, dat moet je natuurlijk ook niet willen, maar dat is, ik weet niet meer precies je vraag, want dat hoor hier niet bij toch?

00:24:23 Interview participant 8: Je mag zeggen wat je wil, het is aan mij een vervolg dat helemaal te ordenen en in orde te krijgen. Maak je daar geen zorgen over. Wat zou jij beschouwen als te gehecht zijn aan een sociale robot? Dat was de vraag.

00:24:41 Interview participant 8: Ja, en dan wordt ruzie maken er hier niet perse bij, dan denk ik wel, dat wat ik heb gegeven, dat dat het wel is.

**10:25 ¶ 115 – 117 in Interview 8 31-03-2021--edited**

mdat het nieuw is, leren kinderen ook beter mee..

00:26:47 Interview participant 8: Ja dat denk ik wel.

00:26:51 MS: En daarom zijn ze enthousiast.

**11:5 ¶ 63 in Interview 9 2021-04-07.mp4--edited**

Aan de ene kant, ja, zoals wat ik zei, dat jongetje dat dan ineens gaat praten omdat het laagdrempeliger is, dan zie ik een positieve factor, ehm. Dus wat dat betreft denk ik dat het zeker wel wat biedt. Ja, maar ik weet niet of dat voor elk kind zo is, dat durf ik niet te zeggen.

**11:6 ¶ 67 in Interview 9 2021-04-07.mp4--edited**

ou, ik denk dat het wel kinderen de drempel over kan brengen en een drempel van hé, ik kan het toch, niemand anders heeft gezien, maar ik heb wel geoefend, een stukje zelfverzekerdheid kan geven, zelfvertrouwen, dus ik denk dat het daarin wel echt een een positieve invloed kan hebben.

**11:7 ¶ 71 in Interview 9 2021-04-07.mp4--edited**

Ze zijn wel heel erg gefocust op de naam, ze willen, ze hebben allemaal ook een naam mogen bedenken en nou ja, daar zijn we ook nog mee bezig, want we hadden voor de kerst tot de kerst, mocht je naam inleven. Na de kerst zijn we een lock down en we hebben heel veel problemen gehad met de corona dus ja dat speelt gewoon echt mee, dus ze zijn er wel echt mee bezig, ja, en en vooral ook de wat oudere kinderen die het programmeren, in mogen programmeren, die ja, die, die zijn er wel echt mee bezig van oké, en nu gaan proberen en lukt het hem. En ja, het is ook voor hem dus, maar het is dus er zit wel een stukje sociale, ja, behoefte of connectie. Ja.

**11:24 ¶ 124 – 125 in Interview 9 2021-04-07.mp4--edited**

Zijn er nog andere, een positieve effecten van de robots die die je bij kinderen ziet behalve de de de verhoogde motivatie die noemt zn verwondering?

00:22:58 Interview participant 9: Ja, ik vind de veiligheid. Ik denk dat ze zich, kinderen die heel zeker zijn, dat die eerder daarmee aan de slag gaan dan, of het antwoord benoemen dan de twijfelaars die dan denk: ik: zeg maar niks, want straks het antwoord fout, en als je dan alleen maar zon kaartje voor hoeft te houden, dan is het natuurlijk al laagdrempeliger, dus dat dat zie ik wel een positief effect hebben.

**○ Social dev. more sensitive**

**20 Quotations:**

**2:22 ¶ 43 in Interview 2 12-02-2021.mp4--edited**

Nou, ik wijk, ik vind ik lastig om te zeggen omdat ik daar denk ik, te weinig echt echt praktijkervaringen, maar je hebt niks nog op kunnen observeren. Ik zou dat dan liever over een langere periode willen kunnen zin om echt ook een verandering te kunnen zien. Het was toevallig bij die leerling dat dat ik dan ook echt in een momentopname al zag, maar ik ben er wel van overtuigd dat zeker een positieve effecten kan hebben op op zouden moeten op dat wel sociaal emotionele ontwikkeling van... ja de hele week voor de klas gestaan dus mijn praten is op... dat je die verbetering wel absoluut kunt zien. Ja.

**2:42 ¶ 5 in Interview 2 12-02-2021.mp4--edited**

n ik merkte dat dat bij de jonge kinderen sowieso heel snel ging. Ik denk dat dat natuurlijk ook wel een beetje de kleuters het eigen van hetjonge kind is dat je natuurlijk heel erg is. Nou, open staat voor dat soort dingen je ben nieuwsgierig je accepteert wat sneller, maar ook bij groep vijf en je zou denken: zon, zon, paartse speelgoed Olifant spreekt dat zon, kind van groep vijf nog aan.

**3:15 ¶ 34 in Interview 1.mp4--edited**

Nou, wat ik vooral zie, is dat ehm een bepaalde type kind vaak de wat... nouja . schuchtere ook wat meer aparte kinderen. Die zijn heel erg getrokken door zo'n robot. En die gaan ook helemaal vol erin waardoor ze zoveel groeien zeg maar in hun zijn, dat het daardoor in de klas ook soms lekker loopt, want je kan ze triggeren of terugpakkenpakken van hé, maar met de robot ging het wel goed is. Dus het is niet zozeer sociale vaardigheden, maar meer hoe inzicht voelt, dus is dat ook een beetje wat je bedoeld, sociaal emotioneel.

**3:16 ¶ 36 in Interview 1.mp4--edited**

Dat, dat heb ik vooral wel, gezien in de tijd dat ik nog op onze school vast werkte, dat ik dat ik die kinderen juist pakte, en niet niet alleen maar de kinderen die dus heel goed waren met de robot, dus alsnog te programmeren, dat ze daar niet alleen de toppers, zeg maar dus de goede kinderen qua niveau. Maar ik vond het juist leuk om de wat kinderen, de kind te pakken die wat lager scoorden, of die juist sociaal wat zwakker zijn, want die gingen wel, die waren dan zo gedreven om met dat ding te werken dat ja, ik vind dat nog leuker dan een kind die het allemaal wel kom en eigenlijk heel snel een interesse verloren en ik kon daardoor ook die kinderen die dat is niet sociaal niet zon sterk stonden, kon ik daardoor juist op een podium zetten, want zij werd dan robot expert zeggen. Zij gingen juist andere kinderen weer leren of de juf helpen dus ze groeide daarvoor in hun hele zijn, zeg maar dat hun plekje in de klas veranderde daarvoor.

**3:17 ¶ 40 in Interview 1.mp4--edited**

Ik wil niet te vele in hoekje zetten, maar ik herken je zo, als ik ze zie lopen op straat. Ik weet zo welke kinderen wat minder goed in een groep staan, nog minder sociaal vaardig zijn dan, en welk kinderen dat wel zijn. Dat heeft te maken met een bepaalde uiterlijke kenmerken. Wat sjofele loop uhm echt letterlijk uiterlijke kenmerken gewoon. Ze zijn wat sukkeliger zeg om met zo negatief te zeggen. Ze zijn soms ook heel snel boos omdat ze zich joh. Ja, ze moet voelen dat ze echt moeten opboksen tegen die andere kinderen. Ja, het zijn vaak een beetje de de de sulletjes van de klas die dat hebben end at kunnen meisjes of jongesn zijn zijn. Maar ja, ik ik ik herken ze zo. Aals ik in de klas staan, herken ik welke kinderen ik moet pakken, jouw moet ik hebben, maar dat is gewoon puur ervaring.

**3:18 ¶ 42 in Interview 1.mp4--edited**

Ja, eigenlijk wel, je weet wel heel snel, ja, je weet welke kinderen wat welke positie in de klas hebben. Dat is een, maar het zijn vaak de sulletjes, de wat de kleding is wat minder mooi, zeg maar of, past niet altijd even goed of van dei snottebellen hebben ze soms. Het zijn van die hele typerende dingen, dingetjes,,, het is heel zielig maar, ik moet het eigenlijk helemaal niet zeggen maar, het is wel zo.

**3:35 ¶ 112 in Interview 1.mp4--edited**

. Ik zie hem wel eerder bij het speciaal onderwijs, daar zie ik hem wel eerder omdat daar ten eerste een hele ook minded, meer open minded manier van lesgeven is, ehm en je daar heel specifiek ook op één ding kan gaan: ja, ik weet niet, ik denk niet dat hij daar beter past, ik weet niet waarom, maar ik denk dat hij daar gewoon beter tot tot zn recht komt en ik heb het niet gezien, maar een collega van mij bij een speciaalonderwijs over ons stichting geweest en daar werd hij echt heel goed ontvangen ehm, want daar zit dan, om het even bij sulletjes te houden, daar zitten de sulletjes van de school dus die gaan wel, en ze krijgen nu niet altijd van dat dat soort dingen, want dat is voor hun niveau niet haalbaar, zeg maar, om het maar even heel negatief te stellen, maar juist die kinderen hebben hier juist baat bij wat ze goeien op één of andere manier krijgen ze lichtjes in hun ogen, want ze zijn vaak natuurlijk onzeker over wat ze kunnen, niet kunnen, als ze van normaal onderwijs vanaf komen. Want ja, daar konden ze vanaflles niet. Dus ik denk dat hij daar gewoon veel beter thuis is. In het regulier onderwijs – ik heb ik – heb ik echt mijn twijfels er over. ja ook omdat ik niet zie dat heel veel scholen het omarm, nog steeds heel weinig scholen die het gebruiken.

**5:13 ¶ 98 – 103 in Interview 3 17-02-2021**

Sociaal zwakere kinderen, kinderen met ASD

Follow up questions, more detail

You could think of issues such as language development, learning, kind/unkind behavior, social interaction, friendschip.

Vooral motivation,en samenwerkingen skills

**5:16 ¶ 113 in Interview 3 17-02-2021**

Sommige wat schuchtere kinderen kwamen erdoor meer los, en gingen ook dingen aan andere kinderen uitleggen.

**5:17 ¶ 123 in Interview 3 17-02-2021**

De misschien wat minder vaardige kinderen, kindderen die vanuit huis al bezig zijn met ICT

**5:18 ¶ 113 in Interview 3 17-02-2021**

Sommige wat schuchtere kinderen kwamen erdoor meer los, en gingen ook dingen aan andere kinderen uitlegge

**6:12 ¶ 95 in Interview 4, 19-2-2021--edited**

kinderen die gewoon heel erg geïnteresseerd zijn in in robots en programeren

**6:13 ¶ 95 in Interview 4, 19-2-2021--edited**

echt sociaal minder, die dat wel heel erg leuk vindt.

**7:21 ¶ 107 in Interview 5 3-3-2021.mp4--edited**

Interview participant 5: Ja, vind ik wel een moeilijke vraag, nou je je merkt vooral dat de kinderen die het lastig vinden om op hun beurt wachten dat die in de knoop komen, want ze willen, hij moet eerst, de robot vraagt iets en soms herhaalt hij het twee keer dat, doe ik dan weer expres, maar na die ene vraag hebben zij het al gehoord en willen ze al reageren. En ik doe natuurlijk twee keer omdat ik weet van, je moet even goed de vraag luisteren, dus je moet ook even rust op jezelf zijn en het moet even binnen komen en sommige kinderen willen natuurlijk heel snel reageren, dat als dat een als dat na drie keer vraag zo is geweest, dan hebben ze wel iets van o ik moet toch even wachten, en vooral die ik in die heel erg impulsief zijn, die ja, dat triggert ze toch wel.

**8:5 ¶ 76 in Interview 6 12-03-2021.mp4--edited**

6: Ja, maar ik kan me wel voorstellen dat als, nouja even heel zwartwit gezegd, de nerd in de klas, zonder aansluiting, die heel goed is in programmeren, heel heel zwartwit zegdt, en die kan de robot toch laten doen en zeggen, maar dan krijg hij [de leerling] wel wat meer aanzien. Dus dat ik misschien meer een gevolg van l zijn of dat. Oh, dat wil ik ook nou, laat maar met jou gaan een samenwerking want jij bent hier goed in, ... onverstaanbaar... in terwijl acht niet meer zo ervan, want het dan dat zou.

**9:8 ¶ 57 – 59 in Interview 7 12-03-2021.mp4--edited**

Interview participant 7: Nou, dat zou niet gek zijn in ieder geval, als dat zo zijn, een robot is een apparaat dat, zeker als je een robot die wij gebruiken, als je die aanzet aanzet dan gaat het toch bewegen en geluid maken, nou, ik denkkinderen die hoogsentitief zijn of die in ieder geval snel geprikkeld zijn door iets wat beweegt. Die zullen daar heel snel zich daardoor aangetrokken voelen en zeker kinderen ook die handig worden met het programmeren gaan daar dan zelf wel, die gaan die robot dingen laten doen die zij willen dat die dat die gaan doen. Zo kun je van een relatie spreken. In feite niet natuurlijk, maar voor een kind. Voelt dat dan wel zo. Dus ja, misschien ook wel.

00:14:31 MS: De de kinderen die hier gevoelig voor zijn, voor voor de voor de robot, die daarmee eerder interacteren, of die eerder zich zelfverzekerder voelen of dat dat leuk vinden, wat wat zou je kinderen typeren? Je noemt wel even hoog, sensitiviteit zijn er ook nog andere typeringen die je zou... of eigenschappen.

00:14:51 Interview participant 7: Ja, jewil natuurlijk niet in labels praten, maar de kinderen die een bepaalde aandachst behoefte hebben die kunnen niet helemaal in opgaan, hè dus. Als je kinderen met ADHD of gewoon kinderen die hoogbegaafdheid, die zouden hierdoor wel op een bepaalde manier , aangetrokken kunnen worden als het in hun straatje past en dan zijn er wel heel veel mogelijkheden om hiermee mee te werken.

**10:7 ¶ 54 in Interview 8 31-03-2021--edited**

Ik heb vooral het idee het hier ook wel een beetje met de leeftijd te maken heeft, en die normaal al wel wat jonger in hun gedrag zijn, dat ze dat sterker hebben dat praten en dat vriendjes gevoe

**10:8 ¶ 56 in Interview 8 31-03-2021--edited**

Het jongentje met autisme wat we denken te weten, want meestal is nog niet vastgesteld op deze leeftijd, maar die, ja moest ergens wel weer wennen, ofzo want het is even wat anders in de klas. Maar omdat het circuit ook nieuw was, moest die daar ook aan wennen. Maar hij reageerde er wel, goed op en nu, iemand ide ik verdenk van ADHD, die waren gewoon enthousiast.

**11:8 ¶ 77 in Interview 9 2021-04-07.mp4--edited**

: Ja, ja, dat zie ik wel. De ene die die kijkt echt naar uit om te mogen oefenen en die die, ja, ik vind het echt een traktatie en de ander, die geeft ze niet zoveel om. En ik vind het degene die die dat als een traktatie ziet die die zie je ook heel erg betrokken bezig. Dus dat zie ik wel een... ja de één heeft het meer dan een andere.

**11:9 ¶ 79 in Interview 9 2021-04-07.mp4--edited**

Mmm het is wel een veelzijdig, vind ik. De ene die is verwondering, die is heel erg snel verwonderd en die ja, of die heeft thuis daar komt daar minder mee in aanraking. Een en ander kind die heel veel ermee in aanraking komt, de bedoel ik mee met met met computers, schermpjes dat soort dingen, die vindt het minder snel speciaal. Ehm ja, dus daar zie ik wel verschillen.

**○ Social dev. neg. impact seen**

**2 Quotations:**

**6:15 ¶ 99 in Interview 4, 19-2-2021--edited**

Nee, het is zo een klein onderdeel, eigenlijk van je lesprogramma, dat dat, nee, dat je dat negatieve

**9:15 ¶ 79 – 80 in Interview 7 12-03-2021.mp4--edited**

Heb je die verbondenheid of die gehechtheid aan een robot ook wel eens in jouw lessen meegemaakt?

00:22:35 Interview participant 7: Nee, nee, en dat dat komt omdat we dan daarvoor eigenlijk kinderen er nog te kort mee werken, en zijn een aantal scholen die hebben vast en robot staan. Dus de kans dat dat ontstaat is er natuurlijk wel, maar ik heb dat nu nog niet gezien.

**○ Socialev. best implementeren**

**11 Quotations:**

**2:25 ¶ 59 in Interview 2 12-02-2021.mp4--edited**

a, die en die de rustig introductie, dat kinderen snappen hoe zon robot werk, want dat is natuurlijk eigenlijk ook wel wat je wilt bereiken. En je kunt zo robot wel inzetten je onderwijs. En dan ja, je kunt 'm nog wel eens klassen assistent inzetten. Dan wordt ook wel gedaan, maar ik denk dat het mooie is, juist als zij weten hoe die robot werkt, waar ze sensoren zitten. Hoe pikt dit geluiden op? Hoe maak je jezelf geluid, hoe beweegt die eigenlijk, zodat je eigenlijk ook meer waardering krijgen voor die robot? En dat zorgt denk ik ook echt voordat ze geïnteresseerd raken in die robot. Want dat is eigenlijk wat je wil, want hoe meer die leerlingen geïnteresseerd worden in die robot en meerdere kinderen onder jouw groepen het horen en weten: daar is een robot. En daar ga je mee werken en dat moet, dat is gewoon de robot van de klas. En ik denk dat, als je dat gewoon goed opbouwt, dan kan ik zo snel even geen andere dingen bedenken van ik zou zeggen: op lange termijn zou voor problemen kunnen zorgen.

**2:43 ¶ 49 in Interview 2 12-02-2021.mp4--edited**

bijvoorbeeld voor dit soort dingen dat ie zelfs nog weleens als je het maar rustig introduceert en je laat ze kennismaken en je moet niet.... niet met zon, pop of met zn olifant in het gezicht douwe en zeggen dit is leuk. Kijk raak aan tof, nee, je moet ze gewoon. Dat ding moet goed neerzetten en laat ze zelf maar uitproberen en zelf die grenzen opzoeken. Dus ik denk denken: ja, je hebt altijd wel risico's met a

**3:23 ¶ 54 – 58 in Interview 1.mp4--edited**

nterview participant 1: Ik vind ik echt heel moeilijk, want dat is een stukje dus totaal niet echt een thuis ben, omdat ik heel erg in die kernvakken taal en rekenen, waarvoor ik veel gebruik. Ik weet of weet ik niet zo goed. Ik zou een stukje gebruik – ik eigenlijk nooit, omdat ik dat best wel lastig vindt, ook omdat het een apparaat is in. Ik kan niet van tevoren bedenken wat de kinderen gaan zeggen als ik het programma maken. Dat maakt het voor mij moeilijk om iets met sociale dingen te doen. Ik natuurlijk wel een lesje maken over hoe we met elkaar omgaan. Maar ja, ik denk ja, ik weet niet of heel moeilijk.

00:17:28 MS: En als je dan stel pakken en wat exactere vakken zoals rekenen bijvoorbeeld en dat je daar de robot voor voor zou inzetten in je klasse, dan is het misschien ook dr. Zie je misschien ook een effect op sociaal gedrag bij kinderen dat ze inderdaad Gods tegen mekaar zeggen, niet doorheen praten.

00:17:46 Interview participant 1: Ja.

00:17:47 MS: Denkt u dat dat een een belangrijk onderdeel is, maar de inzet van robots dat die kinderen die robot wel als sociaal zien.

00:17:56 Interview participant 1: Ja, want dat, dat is wel één van mijn doelen, altijd dat ze dus ik vertel je gaat oefenen, maar je moet ook leren. Hoe zon ding, hoe het werkt, wat je hoe je omgaat met elkaar in mee omgaat bij hogere roepen, als ik dat doe ik er natuurlijk een tijdje fijn als je dan vallen en heb ik hem ook gepakt. En toen heb ik wel uitgelegd van ja, maar dat hoeft niet allemaal met robots te gaan werken. Later moet wel begrijpen wat zon ding is en dat werkt en hoe dat dan in mekaar zit, of wat je dan moet we qua gedrag en dat als het niet lukt, dan moet je met elkaar wel, ja, dus dat soort dingen, dat leg ik wel uit en het wordt ook heel natuurlijk gedaan, vaak bellers toen ik zie wel als een als ik dan op tafel te oefenen gedaan. En dan had één een tweetal lukte: het niet, is de voorganger die ging mee helpen uit zichzelf. Wat in een normale situatie niet, hoe vaak ze niet heel vaak gebeurde, dus hij lokte wel bepaald sociaal contact uit. En ik had ook van joh. Je moet ook wel helpen, want ik ben met de rest van ik zoek het lekker met elkaar. Je kan elkaar helpen doen is dus dat dat ook wat de zeg, maar dat is gewoon dat ze dat deden, want dat was een mooie, het apparaat die daar stond en mochten dr. Mee werken. Dus dat is wel interessant.

**5:19 ¶ 128 in Interview 3 17-02-2021**

Als kinderen helemaal alleen maar met de robot interactie willen, of negatieve ervaring krijgen als de robot niet aanweizg is, dan is het doel voorbij geschoten.

**5:20 ¶ 133 in Interview 3 17-02-2021**

Door een leraar die het in de gaten houd.

**6:16 ¶ 101 in Interview 4, 19-2-2021--edited**

en. Ik denk dat het goed is als je ze daar een keuze in hebben

**8:2 ¶ 72 in Interview 6 12-03-2021.mp4--edited**

Het kan als een soort mentor kunnen zijn, het zou natuurlijk ingezet kunnen worden, als leerkracht die 30 leerlingen in de groep,je hebt oog voor allemaal, maar ze zijn ook maar vijf uur op school, en ja, gedeeld door je 25 leerlingen, dan de tijd gaat hard. Dus op die manier, stel je hebt een kind wat nou ja, gewoon sociale skills, heel erg mist en dan zou het kunnen helpen. Maar, dan denk ik nog steeds dat de kracht van de leerkracht wel groter is, maar het zou een soort aanvulling kunnen zijn. Maar hoe en in welke mate? Ja, dat vind ik lastig om te bedenken, zeg maar.

**8:7 ¶ 84 in Interview 6 12-03-2021.mp4--edited**

Mmm, ik zou het sowieso altijd naast iets zetten, als aanvulling, maar ik denk niet dat een robot de sociale skills van een kind zo kan beïnvloeden dat het kan zijn in plaats van, bijvoorbeeld een kanjer training die wordt gebruikt of een andere sociale vaardigheid methode ehm die het kinderen leert, en wat bijvoorbeeld, kijkt, wat ze een robot natuurlijk wel heel leuk maakt. Dat is ook dat circuit, wat wij dan hebben, heel leuk maakt, is dat kinderen sowieso in aanraking komen met andere kinderen en bijvoorbeeld de kinderen die zo'n lesje maken voor groep drie of vier. Ja, dat is hartstikke leuk, want dan ga je bedenklen. Oké, hoe denken andere kinderen? Hoe maak ik niet te moeilijk, en dat is wel dat je je moet gaan verplaatsen in andere kinderen, hè dus dat jij een les maakt voor een ander, kijk dat heeft natuurlijk heel veel te maken. met sociale denkwijze. En eh bijvoorbeeld met dat circuit ja, dan laat ik ook wel een kind van groep 5 samenwerkingen met een kind van groep, zeven, of groep acht, en als de kinderen van groep acht gaan helpen bij de kleuters of ze hebben ook wel geholpen met de NAo bijvoorbeeld in bijvoorbeeld groep zes, omdat de juf dat dan spannend vindt. Ja, weet je dan, komen die kinderen best wel sociaal aan hun trekken, want ze moeten andere kinderen gaan helpen, dus ja, als je het hebt over sociaal dan zit je meer op dat vlak.

**9:10 ¶ 67 in Interview 7 12-03-2021.mp4--edited**

Nou, wat belangrijk is dat dat een robot contact maakt met met kinderen, dus een robot zal er ook als, uit moeten zien, als iets waar je contact mee kan maken, dus de robot die wij hebben die heeft, het lijkt een klein mens. Het heeft armen en heeft benen, moet er herkenbaar uitziet ogen, een mond. Er is nogal wat te winnen, want alles, er zit geen zelf leerling systemen in op dit moment, dus een robot zal niet uit zichzelf vragen hoe het met je gaat, maar als als dat wel zo gebeuren, dan dan heb je toch een soort maatje eigenlijk waar je je verhaal kwijt kunt, een robot zal ook niet verder vertellen, als je een geheim tegen hem zegt, maar dan aan de andere kant, zal dan ook de beveiliging goed op orde moeten zijn, want dat hebben we ook gemerkt dat sommige dingen zijn er gewoon niet goed beveiligd. Daar zit er wel, dan ga je wel weer wat wat over ethiek praten, maar daar zitten wel haken en ogen aan, aan het gebruik van een robot. Maar een robot moet in ieder geval herkenbaar zijn voor voor kinderen. Je gaat ook niet je geheim aan je iPad vertellen, hè, zoals je het wel in een dagboek zou schrijven. Het moet iets fysiek zijn.

**9:12 ¶ 71 in Interview 7 12-03-2021.mp4--edited**

Nou, ik vind dat het verstandig om in ieder geval nooit alleen met een robot te werken, maar altijd in een in een tweetal of een drietal. Dan moet je namelijk sowieso al onderling met zn tweeën of met zn drieën communiceren Dan stimuleer je ook die communicatie. Dat, dat zou ik sowieso doen, en dat doen we ook altijd. Dat is ook omdat we in een school één robot hebben, dus dan is het ook efficiënt als je met meerderen tegelijk natuurlijk aan werk. Maar je wil ook juist die die onderlinge communicatie stimuleren. Verder ja, vooral dat. zorgt ervoor dat kinderen juist onderling communiceren, niet alleen met de robot, of dat nou gaat om het programmeren van de robot, doen we altijd in een groepsvorm, maar ook het de verwerking of het oefenen, eigenlijk ook altijd wel met minimaal twee, want dan dan stimuleer je praten juist.

**10:10 ¶ 64 in Interview 8 31-03-2021--edited**

Nou, zeker in het begin en op deze leeftijd zou ik eerst nog wel veel er bij zijn of in dezelfde ruimte inderdaad en ideaal zou zijn eigenlijk wel uit de klas, nu worden kindreen wel eens afgeleid, .... onhoorbaar, verbinding valt weg...

**○ Succes factors**

**15 Quotations:**

**6:8 ¶ 71 – 72 in Interview 4, 19-2-2021--edited**

MS: Zou dat wat jou betreft, ook nodig zijn om de robot op een juiste manier in te zetten. Dat leerkrachten eerst een introductie krijgen of een les samen met een ervaren leerkracht met de robot en dan zelf aan de slag.

00:20:08 Interview participant 4: Eh ik denk dat dat ze heel erg helpt, omdat je eerst heel erg aan het stoeien bent. Hoe het allemaal in elkaar zit, en dat is eigenlijk gewoon, aangezien je zo veel vakken geeft op basisschool. Is het gewoon echt zonde van je tijd. Qua snelheid is het gewoon heel efficiënt om eh om het samen te doen, en ik heb een soort introductie kaartjes die dan met de leerlingen doen. En dan kan ene leerkracht eigenlijk meteen meedoen, waarin eigenlijk alle functies van de robot langskomen. Het is gewoon allerlei dingetjes laten en dat ze alle functies kennen. Dat is gewoon je eerste les, en daarna kun je kijken van bij welk vak ga ik hem laten aansluiten.

**6:30 ¶ 131 in Interview 4, 19-2-2021--edited**

astig vinden om te leren ook af en toe zo robot inzetten, het enige wat je natuurlijk tegen aan gaat lopen in het huidige systeem is dat je gewoon 30 kinderen in klas zitten. Je kan niet even 1 op 1 met een robot op de gang gaan zitten, dus je hebt ze ook gewoon veel te veel kinderen, en je de hele groep is dan van slag als er één met robot mag.

**6:31 ¶ 133 in Interview 4, 19-2-2021--edited**

Ik denk dat dat, wij hebben bijvoorbeeld extra hulp in de klas, de mensen die apart gaan zitten rtérs of andere die met kinderen aan de slag gaat, dat die eigenlijk die vaardigheden, ook gaan leren en dat die sneller een robot erbij pakken.

**6:34 ¶ 135 in Interview 4, 19-2-2021--edited**

Hier in de school hebben eigenlijk alle kinderen krijgen een aantal leessen op een chrome book, dus alle leerkrachten kunnen met een chromebook werken, kinderen kunnen er mee werken. Dus de drempels worden wel steeds kleiner, en ik denk door de ontwikkelingen omdat heel veel digitale is dat het ook steeds kleiner gaat worden, waardoor het steeds makkelijker gaat worden om dan de robot de klas in te schuiven.

**7:3 ¶ 89 in Interview 5 3-3-2021.mp4--edited**

Wat ik ook wel een beetje zeg, sowieso een team die acht je staat, de directie die achter je staat, een team die achter je staat en iemand die tijd heeft om aansturen. Ik, ik ben dan die ICT coördinator en ik heb ook een dag uit de klas. Ik weet dat er ook scholen zijn, en dat hebben kleine scholen natuurlijk meer, want die hebben minder uren, maar dan is dit is gewoon heel moeilijk, om dit te doen. Ik merk wel door – dat zei het net al - doordat we meer ook wat een klas assistenten krijgen wordt het die gaan, ben ik ook gedeeltelijk ook aan instrueren om in die robot aan de gang te gaan, want die kan ook met hun – die kunnen makkelijker met groepjes kinderen dingen doen, want het kan best wel met een groepje. Maar als je als leerkracht van groep vijf denkt van hé in dit circuit, ga ik even die robot inzetten – moet die robot wel gehaald worden, opgestart worden – en we hebben het wel al over gehad om ook in elke groepen kinderen daarvoor aan te wijzen – maara die moeten dat ook het weer geïnstrueerd krijgen – en dat is het een beetje.

**9:20 ¶ 108 in Interview 7 12-03-2021.mp4--edited**

Ja, ja, maar dat is niet alleen voor de robot, maar dat geldt eigenlijk voor alles. Wat ik zeg, boeken zet je ook in daar waar nodig is. En als je die niet nodig hebt, moet je ze vooral niet inzetten. Zorgt voor afwisseling. Kinderen houden ook van afwisseling, zeker in de huidige tijd. Kinderen vinden het prettig om afwisselend te leren en elk kind leert ook anders. Dus je moet ook zorgen dat je iets afwissend aanbiedt, dus ook niet een robot de hele dag elke dag aan, dat hoeft niet oké, wees, daar mag je ook wel terughoudend in zijn.

**9:21 ¶ 110 in Interview 7 12-03-2021.mp4--edited**

Niet inzetten om het inzetten

**10:9 ¶ 60 in Interview 8 31-03-2021--edited**

Ik denk vooral wel op reken gebied dat dat het makkelijkste is en met kleine groepjes. Denk ik. Dan kun je, omdat de robot ook wel bijvoorbeeld resultaten kan bijhouden. Is dat wel snel een controlemiddel. En wij wilden het hier op school ook inzetten voor kinderen die vastliepen in het werk, bijvoorbeeld, dat als je dan als je op de linker voet drukt dat er staat lees je opdracht maar dat kost best wel veel programmeerwerk, denk ik nog. En ik weet niet hoe snel dat ingezet gaat worden, wel mooi doel, maar ik zie nu nog niet zo voor me.

**10:11 ¶ 72 in Interview 8 31-03-2021--edited**

: Ik weet dat ik ook ouders begon, want oh ja, ik merk wel als ik met een klein groepje bezig ben en een andere groep met een robot, dat het ook wel eens af leidend is. Dus ideaal lijkt mij, als ze dat groepje op de gang zit met een bijvoorbeeld een ouder.

**10:28 ¶ 127 – 128 in Interview 8 31-03-2021--edited**

: Hoor ik ook een beetje tussen de regels door, dat je zegt: de leerkracht moet dat eigenlijk ook kunnen inschatten wanneer het te gehecht raakt. Die moet je moet als wij in de loop, in de loep blijven.

00:28:57 Interview participant 8: Ja, ik denk wel dat die meestal ook de kinderen ziet en dan weten het verstandig is om juist wel of niet in zetten.

**10:32 ¶ 139 – 141 in Interview 8 31-03-2021--edited**

Waarom is de aanwezigheid van die ouderen of een begeleider belangrijk?

00:31:25 Interview participant 8: Omdat ik denk dat, als wer wat fout gaat, kost het als ik wil helpen te veel tijd, tussen de klas en een robot te wisselen. Maar dat is zeker bij jonge kinderen, ik denk dat dat bij oudere dat het dan wel beter gaat.

00:31:44 MS: Oké, helder, dat waren eigenlijk al mijn vragen al. Hartstikke bedankt voor dit voor dit interview. Ik sluit af met een laatste vraag, en dat is: ken jij nog andere docenten die eventueel mee zouden willen doen aan dit interview.

**10:40 ¶ 61 in Interview 8 31-03-2021--edited**

kleinere groepjes omdat de robot dan eigenlijk een betere bijdrage kan leveren en ook het bijhouden van bijvoorbeeld e-portfolio of resultaten

**11:11 ¶ 89 in Interview 9 2021-04-07.mp4--edited**

Het ideaalplaatje dus wat mij betreft, de kinderen zijn in de groep aan het werk en er gaan kleine groepjes op enkelingen met de robot op de gang de activiteiten uitvoeren en zo zou ik het, zo zie ik het, en dan het liefst in de cyclus achterelkaar door.

**11:26 ¶ 129 in Interview 9 2021-04-07.mp4--edited**

Ja, door door gewoon ook één verantwoordelijke, die wij dus nu hebben, maar ook constant levendig te houden, door te benoemen, maar ook in de team momenten aan te kaarten van wat is er gebeurd? Hoe kunnen we het aanscherpen? wat kunnen we nog meer? Ja, omdat gesprek vooral, ja, echt in je beleid te te borgen van de aanpak. Dat is belangrijk, maar het moet wel echt een drive zijn om het om het in te zetten, want ik zie de voordelen zeker dan alleen. het is gewoon wel arbeidsintensief nog, en ik denk dat het wel beter gaat, maar nu is het heel intensief.

**11:35 ¶ 94 – 95 in Interview 9 2021-04-07.mp4--edited**

Moeten nog iemand bij zijn, verder naast de robot?

00:17:09 Interview participant 9: In deze setting niet, nee, misschien bij de kleuters kan het nog wel meerwaarde zijn, maar dat het zou zelfstandig moeten kunnen.

**○ Too attached: age diff.**

**1 Quotations:**

**2:55 ¶ 93 in Interview 2 12-02-2021.mp4--edited**

wanneer je het gevoel zou hebben dat deze robot bijvoorbeeld echt emoties zouden hebben, dat ze echt pijn zouden kunnen voelen. Maar ja, aan de andere kant kun je dat een kleuter kwalijk nemen wanneer hij bang is dat als als als olifant van de tafel afloopt, dat die misschien pijn heeft aan zn snuit en zou dat dan met een verlies van van realiteit gevoel zijn. Ik denk niet, dat is gewoon een jong, denk kind of hoe een jong kind denkt, dus dat vind ik heel lastig dan leg dan denk ik ook heel erg aan met wat voor leeftijd je te maken hebt.

**○ Too attached: less human contact**

**8 Quotations:**

**2:53 ¶ 95 in Interview 2 12-02-2021.mp4--edited**

geen ruimte meer over te houden voor het sociale contact met de andere die groep. Ik denk dat dat moment zorgwekkend woord, want je wilt eigenlijk dat zon robot tot ingezet om niet alleen de sociale ontwikkeling van jezelf, maar met name door met je klasgenoten daaraan te werken

**2:54 ¶ 96 in Interview 2 12-02-2021.mp4--edited**

als die robot het centrale punt wordt en dat daardoor eigenlijk de de vriendschap relaties of de andere relaties met je klasgenootjes of met de menselijke docent daardoor in het gedrang komen

**5:31 ¶ 128 in Interview 3 17-02-2021**

Als kinderen helemaal alleen maar met de robot interactie willen,

**5:33 ¶ 163 in Interview 3 17-02-2021**

Denk aan zelfde soort idee als game verslaving, helemaal online leven, en zo ook minder contact hebben met vrienden op school en daar ook de aansluiting gaan missen, minder uitngeodigd worden op verjaardagen etc.

**6:52 ¶ 121 in Interview 4, 19-2-2021--edited**

Dus dingen vertellen aan de robot eh zich vaker dingetjes vertellen aan de robot dan aan de juf dat, van dingen die ze mee hebben gemaakt, klein of dat ze weer binnenkomen of buitenspelen is wat er gebeurt, dat ze dan even langs langs de robot lopen om om hun hart te luchten, zo zou ik dat zien, terwijl ik denk, ja, dan ga je niet meer sociale interactie aan dat je met je klas of met je juf, maar dan probeer je dat bij een robot te halen.

**6:53 ¶ 122 in Interview 4, 19-2-2021--edited**

us als het een negatief effect heeft op de sociale interactie met klasgenootjes of met met de leerkracht

**8:30 ¶ 96 in Interview 6 12-03-2021.mp4--edited**

Dat dan de sociale component weg gaat en dat je alleen nog maar sociaal contact heb met een robot. dat je geen vriendjes meer hebt, maar je... en ja. ik denk dat dat het is.

**8:31 ¶ 102 in Interview 6 12-03-2021.mp4--edited**

je daardoor, als je alleen de robto als vriend ziet en geen geen andere vriendjes hebt, als je tegen de robot praat alsof het een mens is, en als je de robot ziet waarheid.

**○ Too attached: not seen**

**4 Quotations:**

**3:29 ¶ 91 – 92 in Interview 1.mp4--edited**

MS: Maar je zegt niet dat ze dat later in haar leven problemen van krijgen.

00:30:10 Interview participant 1: Nee, nee.

**5:34 ¶ 163 in Interview 3 17-02-2021**

Maar op de manier waarop we nu robots inzetten zie ik weinig risico's.

**6:51 ¶ 121 in Interview 4, 19-2-2021--edited**

Ik denk dat je het in het onderwijs natuurlijk niet heel snel krijgt, alleen als je een robto altijd in de klas hebt, kinderen die die als te gehecht... dat zou er voor mij uit zien, dat ze op zich heel erg elke keer richten tot de robot.

**8:32 ¶ 104 in Interview 6 12-03-2021.mp4--edited**

Nee, want dan is het nog een schoolse setting en is het eigenlijk een handlanger van de leerkracht. En ik denk dat dan het wordt gebruikt als ondersteuning alsof het een soort onderwijsassistent is.

**○ Too attacheed: upset when the robot is not present**

**3 Quotations:**

**5:32 ¶ 128 in Interview 3 17-02-2021**

of negatieve ervaring krijgen als de robot niet aanweizg is, dan is het doel voorbij geschoten.

**9:30 ¶ 84 in Interview 7 12-03-2021.mp4--edited**

Nou dat dat kinderen echt verdrietig zijn als hij er een dagje niet is, bijvoorbeeld, of dat zo overstuur raken als je niet doet wat ze willen dat die doet, dan denk ik wel dat je te te gehecht raakt of of ja, zoals zoals kinderverliefdheid, weet je, dat ze zeggen van ik wil trouwen met een robot, dat soort dingen, dan moet je wel oppassen, dan ga je de verkeerde kant op.

**11:15 ¶ 105 in Interview 9 2021-04-07.mp4--edited**

In in de zorg en heimwee misschien ook wel daarna of geobsedeerd erdoor ik denk dat het op die manier zich zou kunnen uiten

**○ Use, easy**

**1 Quotations:**

**6:4 ¶ 12 in Interview 4, 19-2-2021--edited**

Op zich is de werking is de werking van een NAO robot wel makkelijker als je het één keer heb gedaan dan weet je echt hoe het werkt.

**○ Use: high cost**

**2 Quotations:**

**3:40 ¶ 112 in Interview 1.mp4--edited**

hele kostbare dingen waar het basisonderwijs het geld niet, geen geld voor heeft, dus dat is een stuk financieel

**8:22 ¶ 67 in Interview 6 12-03-2021.mp4--edited**

niet sociale robot als het ware, die zijn gewoon goedkoper en misschien makkelijker, waardoor er meer kan inzetten,

**○ Use: management supprt**

**3 Quotations:**

**3:41 ¶ 112 in Interview 1.mp4--edited**

maar het wordt niet gevoerd in het onderwijs.

**3:42 ¶ 114 in Interview 1.mp4--edited**

Die resultaten heb je nog niet en dat maakt dat het nog niet met onderzoek bevestigd kan worden en daarvoor krijgen we geen directeuren mee of stichting mee of wat dan ook. Dus dat is gewoon heel lastig. Ja.

**7:20 ¶ 85 in Interview 5 3-3-2021.mp4--edited**

groei van de leerkracht die nog, en dat, dan denk je nou ja, als we twee jaar verder zijn, dan weten ze het wel, maar nee, want in die twee jaar hebben ze allemaal andere dingen ook gekregen, want er is ook nog een nieuwe rekenmethode een nieuwe taalmethode, dus je moet iemand echt wel gemotiveerd zijn om dat heel erg... Dat is met kinderen ook, hoelang duurt het voordat ze tafels geautomatiseerd hebben, en dat is met leerkrachten ook,

**○ Use: motivation**

**5 Quotations:**

**2:38 ¶ 26 in Interview 2 12-02-2021.mp4--edited**

stuk prikkelender maken voor kinderen.

**6:40 ¶ 131 in Interview 4, 19-2-2021--edited**

het is gewoon iets magisch robots. Kinderen raken tochze kijken toch weer met verwondering naar een presentatie van een robot, of het prikkelt ze

**10:35 ¶ 35 in Interview 8 31-03-2021--edited**

kinderen zijn heel enthousiast en zijn ook blij, we hebben meerdere rondes, en ze zijn al blij als ze weten: de volgende ronde mag ik, ze zijn wel echt heel enthousiast erover.

**11:28 ¶ 59 in Interview 9 2021-04-07.mp4--edited**

. Het is speciaal ook wel... maar het is verwondering ook wel

**11:29 ¶ 61 in Interview 9 2021-04-07.mp4--edited**

voelen ze zich wel meer verbonden, dan met een computer.

**○ Use: motivation_2**

**6 Quotations:**

**3:46 ¶ 48 in Interview 1.mp4--edited**

ik had één leerling kleuter destijds, die wilde totala niet leren, dat komt niet veel voor, maar die wilde echt, die had helemaal geen interesse in lezen of in letters of in rekeken of nog wat, maar die robot... dat was me toch echt wel hoor. Zodra die robot was, dan deed ie alles.

**3:47 ¶ 48 in Interview 1.mp4--edited**

bij mij klapte hij gewoon dicht, maar bij de robot dus wel.

**3:51 ¶ 48 in Interview 1.mp4--edited**

t 1: Ja, ik had één leerling kleuter destijds, die wilde totala niet leren, dat komt niet veel voor, maar die wilde echt, die had helemaal geen interesse in lezen of in letters of in rekeken of nog wat, maar die robot... dat was me toch echt wel hoor

**5:29 ¶ 103 in Interview 3 17-02-2021**

motivation,en samenwerkingen skills

**6:45 ¶ 83 in Interview 4, 19-2-2021--edited**

En ik denk ook bijvoorbeeld, want ik wil het eigenlijk wel inzetten bij kinderen die bijvoorbeeld eh die we moeilijk aan het werk krijgen, voor rekenen, dat ze die sommetjes laten doen bij de robot, bijvoorbeeld omdat ze toch leuk, dat vinden ze toch leuk, dat willen ze eigenlijk toch wel graag de interactie met een robot. Dus als je dan gaat rekeken en robot die gaat sommetjes zeggen ja, dan weet je dat toch wel leuk als de robot zegt hé top!

**10:36 ¶ 48 in Interview 8 31-03-2021--edited**

Ik zie vooral heel veel enthousiasme. Ik denk dat dat het is, en dat praten zie ik ook wel, maar of ze het daardoor anders doen, dat ik ik niet, nee.

**○ Use: place in education?**

**16 Quotations:**

**2:36 ¶ 26 – 28 in Interview 2 12-02-2021.mp4--edited**

Ja, dat denk ik wel. Zeker als ik dan in ieder geval kijken vanuit een alpha mini omdat ik die lessen niet aan het ontwerpen ben, zie je dat het echt een manier kan zijn om bepaalde lessen echt te verrijken. Dus dat kan een les een stuk prikkelender maken voor kinderen.

00:12:50 MS: Mmm.

00:12:51 Interview participant 2: En ik denk ook op sociaal gebied, zeker, hè, je kunt je kunt 'm laten reageren op bepaalde dingen die hij hoort. Dus als je in de klas zet en is dat in dat die bij een bepaald volume van geluid binnen het lokaal een reactie geeft. En je hebt dat moment afgesproken met de kinderen werken nu fluisterstem en veel mensen zetten de leerkracht te zetten in het stoplicht in bijvoorbeeld, hè dat je rood is, gewoon stilte oranje, de stem en groen is mag vragen stellen: je mag gewoon overleggen. Een robot kan dan eigenlijk een soort controle middel zijn, maar dat kan ook voor die kinderen een middel zijn van oh wacht, hij luistert en hij is ons hulp, je onze maatje op dat moment om ons eraan te helpen herinneren dat we echt een te hoog volume hebben. Ik denk, als je die op dat soort momenten in zn als een soort klasse assistenten zou je het dan eigenlijk als het ware zijn, dan denk ik ook echt wel dat de kinderen daar ook echt te erkennen als een extra persoon in dat lokaal. Maar dat is puur hypothetisch nu, want dat heb ik zelf nog niet kunnen proberen. Natuurlijk. Dat zou ik wel heel graag, willen dat wel nog een keer te doen, maar die meerwaarden zie ik er zeker van in,ik denk wel wat je net ook al zelf aangaf dat veel leerkrachten het wel eens een drempel zullen aanvragen of zoiets in de klas te zetten. Zeker als je al een een ja docent bent die al een heel aantal jaren in het in het vak zit, en dan kom je ineens met zoiets. Ik denk wel, dat probeer ik aan mijn lessen ook altijd op te letten dat het zo ontworpen wordt dat de drempel zo laag mogelijk wordt, dat je alles zo duidelijk mogelijk eigenlijk al dat je alles over de leerkracht hebt gedaan en dat alleen maar de robot een lokaal of te zetten en hij heeft een lespakket voor zich en hij weet precies wat ie eruit kunnen halen hij of zij trouwens en hij weet precies welke programmeer stap hij zou kunnen zetten, maar ook genoeg ruimte bieden ze dat de leerkracht zelf ook nog dingen kan toevoegen. Kan aanpassen, ik denk dat ja dat dat eventueel de drempel zou kunnen verlagen, want ik kan me voorstellen dat... ik ik ik ben met robots gewend te werken, maar ik kan me voorstellen dat andere leerkrachten dat wat moeilijker vinden.

**2:37 ¶ 24 in Interview 2 12-02-2021.mp4--edited**

dan ook echt op op op vakdidactisch gebied, is om leerinhouden over te brengen of eigenlijk, ongeacht

**3:39 ¶ 112 – 114 in Interview 1.mp4--edited**

Mijn eerste gevoel zegt nee, omdat ik... ten eerste zijn het hele kostbare dingen waar het basisonderwijs het geld niet, geen geld voor heeft, dus dat is een stuk financieel en ten tweede is het niet tot nu toe zie ik hem niet nog de opbrengst leveren die die zou kunnen leveren, die bijvoorbeeld een lesprogramma of een computerprogramma die ook of met de kinderne kan leveren. Ik weet wel dat hij ook op sociaal gebied veel meer bij kinderen teweegbrengt. Ook vanwege de emotioneel betrokkenheid die kinderen kinderen hebben, dus ik weet dat leren dieper gaat, dat weet ik, maar het wordt niet gevoerd in het onderwijs. Ik zie hem wel eerder bij het speciaal onderwijs, daar zie ik hem wel eerder omdat daar ten eerste een hele ook minded, meer open minded manier van lesgeven is, ehm en je daar heel specifiek ook op één ding kan gaan: ja, ik weet niet, ik denk niet dat hij daar beter past, ik weet niet waarom, maar ik denk dat hij daar gewoon beter tot tot zn recht komt en ik heb het niet gezien, maar een collega van mij bij een speciaalonderwijs over ons stichting geweest en daar werd hij echt heel goed ontvangen ehm, want daar zit dan, om het even bij sulletjes te houden, daar zitten de sulletjes van de school dus die gaan wel, en ze krijgen nu niet altijd van dat dat soort dingen, want dat is voor hun niveau niet haalbaar, zeg maar, om het maar even heel negatief te stellen, maar juist die kinderen hebben hier juist baat bij wat ze goeien op één of andere manier krijgen ze lichtjes in hun ogen, want ze zijn vaak natuurlijk onzeker over wat ze kunnen, niet kunnen, als ze van normaal onderwijs vanaf komen. Want ja, daar konden ze vanaflles niet. Dus ik denk dat hij daar gewoon veel beter thuis is. In het regulier onderwijs – ik heb ik – heb ik echt mijn twijfels er over. ja ook omdat ik niet zie dat heel veel scholen het omarm, nog steeds heel weinig scholen die het gebruiken.

00:39:05 MS: En hoe komt dat?

00:39:08 Interview participant 1: De meerwaarde wordt niet gevoelt. Het.. kijk ik, ik weet dit en jij weten de Elly weten het dat het een diepere laag van leren geeft, dat het door de emotie... dat het idee is dat het beter is dan een schermpje, alleen het is nog niet zo in het onderwijs, dat dat ze dat ook begrijpen en dat het effect, en we hebben nog geen effect kunnen meten ervan. Dat maakt dat het nog niet ingebed is, zeg maar in het onderwijs. Stel dat je wel kan zeggen, we hebben onderzoeken gedaan van een aantal jaren en en we weten gewoon dat het significant beter leert of anders leert, of voor die en die kinderen, maargoed daar zijn we nog mee bezig. Die resultaten heb je nog niet en dat maakt dat het nog niet met onderzoek bevestigd kan worden en daarvoor krijgen we geen directeuren mee of stichting mee of wat dan ook. Dus dat is gewoon heel lastig. Ja.

**5:12 ¶ 89 in Interview 3 17-02-2021**

Ja dat zeker, vooral voor motivatie. Platform nu is nog niet helemaal wat ik er van verwacht, je moet er nu nog veel tijd in stoppen

**6:27 ¶ 127 in Interview 4, 19-2-2021--edited**

Ja, ja, nou ech, ik ben er helemaa voor, niet voor niets het goeipunt he.

**6:28 ¶ 129 in Interview 4, 19-2-2021--edited**

Ja, ik vind robotica en programmeren een mooie toegevoegde waare

**6:29 ¶ 131 in Interview 4, 19-2-2021--edited**

Verschillende dingen, het stukje programmeren, vind ik een vind ik heel erg waardevol omdat het een andere manier van denken is, en de problemen, oplossingen en daar zit veel creativiteit in die je in andere vakken niet op die manier kan uitleggen. En ik denk dat je kinderen, het is gewoon iets magisch robots. Kinderen raken tochze kijken toch weer met verwondering naar een presentatie van een robot, of het prikkelt ze op een andere manier. Zoals we nu bezig zijn met VR in het onderwijs, omdat je kinderen op een andere manier de les wat laten ervaren. Kun je door een robot ook kinderen op een andere manier laten ervaren, waardoor het soms beter blijft hangen. Elk kind leert natuurlijk op een andere manier. Dus dit is weer een andere mooie manier om om erbij te doen, en ik denk ook dat sommige kinderen helpt bijvoorbeeld , dan hoef je geen spreekbeurt meer te geven, dan laat je dat lekker door een robot doen. Neem je heel veel stress bij kinderen weg. Ja, ik denk dat je, maar dat is ook wel een beetje wat ik wilde heel graag ook inderdaad met kinderen die

**7:2 ¶ 85 in Interview 5 3-3-2021.mp4--edited**

Ja, ik denk dat ik denk dat er zekere ruimte is voor de robot maar, wat mij ehm. er moet wel iemand zijn die dat aanstuurt. Het staat of valt op dit moment wel met mijn rol, en dat heeft alles te maken met de werkdruk, en de groei van de leerkracht die nog, en dat, dan denk je nou ja, als we twee jaar verder zijn, dan weten ze het wel, maar nee, want in die twee jaar hebben ze allemaal andere dingen ook gekregen, want er is ook nog een nieuwe rekenmethode een nieuwe taalmethode, dus je moet iemand echt wel gemotiveerd zijn om dat heel erg... Dat is met kinderen ook, hoelang duurt het voordat ze tafels geautomatiseerd hebben, en dat is met leerkrachten ook, alleen dat gaat in jaren en daar hangt het ook nog van je team af, want het is zo specifiek wat je zelf ook... het is een heel klein gedeelte, dus op het moment dat wij in, dat hebben we ook meegemaakt, ook met die robot en ook onder andere met drie d printen. Toen ging de leerkracht weg en er kwamen leerkracht erbij? Ja, ik kom weer overnieuw beginnen, snap je het probleem, dus het, moet, het hangt ook samen met wat voor team heb je , heb je een beetje een vast team. Die blijven ze een beetje in dezelfde groep. Want als ik die leerkracht van groep zeven net een beetje zo ver heb en hij gaat daarna naar groep vijf, dan heb je ook een probleem.

**8:20 ¶ 67 in Interview 6 12-03-2021.mp4--edited**

dat ik je je goed begrijp, de de sociale robot is, is een mooie aanvulling op programmeer onderwijs. Alleen de andere robot die er al zijn, die niet sociale robot als het ware, die zijn gewoon goedkoper en misschien makkelijker, waardoor er meer kan inzetten, tegelijkertijd, en daarom ja, eigenlijk beter een rol hebben in het onderwijs.

**9:1 ¶ 38 in Interview 7 12-03-2021.mp4--edited**

Ja die hebben zeker een plek.

**9:2 ¶ 40 in Interview 7 12-03-2021.mp4--edited**

Wat wij merken – en dat is wat wat wat onderzoek ook wel uitwijzen – is dat dat kinderen op een andere manier leren van een robot. Ze gaan toch een bepaalde de verbinding aan met met derobot. Je ziet dat het ene kind leert makkelijk uit het boek andere kind leert weer makkelijker van een scherm met interactiviteit en een robot geeft weer een extra dimensie aan het onderwijs, en zeker als kinderen zelf de robot gaan programmeren, daar tegelijk ook fysiek iets aan kunnen raken iets kunnen zien, dan krijg je wel een andere ander effect dan je op een scherm een programmaatje maakt, of dat je een boek leest en dat kan heel goed, en dat moet ook allemaal naast elkaar bestaan, want het is niet het ultieme middel om met een robot te werken, helemaal niet, maar het is wel één van de middelen waarmee je dat kinderen voorbereid op een toekomst.

**10:1 ¶ 35 in Interview 8 31-03-2021--edited**

Wat ik gezien heb, rekenen dat kan die echt goed, en dan met die kaartjes werkt het ook heel goed, met lezen vind ik het nu nog lastiger, maar de kinderen zijn heel enthousiast en zijn ook blij, we hebben meerdere rondes, en ze zijn al blij als ze weten: de volgende ronde mag ik, ze zijn wel echt heel enthousiast erover.

**11:1 ¶ 53 in Interview 9 2021-04-07.mp4--edited**

Ik vind het echt een meerwaarde. Het enige nadeel is wel de intensiteit. Het is voor de de degene die de robot instelt en klaarzetten is het wel echt even intensief en daar daar zit nog wel een belemerende een factor, vind ik.

**11:3 ¶ 57 in Interview 9 2021-04-07.mp4--edited**

Ja, dus onwennig , maar ik, ja, ik vind wel dat kinderen ook weg wel wat winst uit halen. Je hebt de computer, maar een robot is nog wel. Ja, die biedt gewoon meer.

**11:4 ¶ 58 – 59 in Interview 9 2021-04-07.mp4--edited**

Wat biedt die dan meer?

00:10:17 Interview participant 9: De feedback maar toch ook een beetje de het gevoel, ja, dat klinkt... Ik zie kinderen dan ook echt.... Het is speciaal ook wel... maar het is verwondering ook wel.

**11:10 ¶ 87 in Interview 9 2021-04-07.mp4--edited**

Ik zie het wel eens echt als een ondersteunend middel. Dat is ook de reden dat er op deze manier inzetten. Natuurlijk, als je echt een goed programma hebt, dan zie ik het ook echt zo dat dat de constant groepjes achterelkaar gewoon gaan trainen. Echt de remedieren, van van de vaardigheden die al eerder geleerd zijn. Ik zie niet een robot echt instructies geven, want daar zie ik meer. Ja, dat is nog meer specifieker en op maat. Maar ja, wij hadden remedieren, of het herhalen. Ik denk dat dat echt wel een meerwaarde kan zijn in het onderwijs, ja, en daar ook ondersteunend in het onderwijsaanbod

**○ Use: role**

**11 Quotations:**

**2:39 ¶ 28 in Interview 2 12-02-2021.mp4--edited**

Een robot kan dan eigenlijk een soort controle middel zijn

**2:40 ¶ 28 in Interview 2 12-02-2021.mp4--edited**

soort klasse assistenten zou je het dan eigenlijk als het ware zijn, dan denk ik ook echt wel dat de kinderen daar ook echt te erkennen als een extra persoon in dat lokaal.

**2:41 ¶ 24 in Interview 2 12-02-2021.mp4--edited**

vakdidactisch gebied, is om leerinhouden over te brengen of eigenlijk

**3:44 ¶ 44 in Interview 1.mp4--edited**

Natuurlijk in de toekomst is – en dat maakte wel dat ik het ja die robot eigenlijk kon gebruiken om hun tot leren te krijgen een aantal kinderen. Niet allemaal, maar het was wel vaak een motivator.

**6:37 ¶ 131 in Interview 4, 19-2-2021--edited**

stukje programmeren

**6:38 ¶ 131 in Interview 4, 19-2-2021--edited**

gewoon iets magisch robots.

**6:39 ¶ 131 in Interview 4, 19-2-2021--edited**

Kun je door een robot ook kinderen op een andere manier laten ervaren, waardoor het soms beter blijft hangen

**8:21 ¶ 67 in Interview 6 12-03-2021.mp4--edited**

mooie aanvulling op programmeer onderwijs.

**9:23 ¶ 40 in Interview 7 12-03-2021.mp4--edited**

het is niet het ultieme middel om met een robot te werken, helemaal niet, maar het is wel één van de middelen waarmee je dat kinderen voorbereid op een toekomst.

**9:24 ¶ 40 in Interview 7 12-03-2021.mp4--edited**

Je ziet dat het ene kind leert makkelijk uit het boek andere kind leert weer makkelijker van een scherm met interactiviteit en een robot geeft weer een extra dimensie aan het onderwijs, en zeker als kinderen zelf de robot gaan programmeren,

**10:34 ¶ 35 in Interview 8 31-03-2021--edited**

b, rekenen dat kan die echt goed, en dan met die kaartjes werkt het ook heel goed, met lezen vind ik het nu nog lastiger,

**○ Use: speech**

**3 Quotations:**

**6:46 ¶ 85 in Interview 4, 19-2-2021--edited**

Nou, taalontwikkeling is wel een dingetjenatuurlijk, want hij spreekt dingen nog wel eens raar uit.

**9:27 ¶ 48 in Interview 7 12-03-2021.mp4--edited**

e hebben op een school waar kinderen, waar een aantal kinderen uit een andere de cultuur komen. Die praten moeilijk Nederlands die praten thuis geen Nederlands. Die vinden het lastig om in het openbaar te spreken en een robot helpt daarbij en en helpt dus bij hun eigen taalontwikkeling, waardoor ze ook makkelijker contact maken met leeftijdsgenoten.

**10:12 ¶ 76 in Interview 8 31-03-2021--edited**

Nou, ik denk wel dat hij lange woorden maakt hij nu, daar hapert hij wel eens in, dat is niet helemaal vloeiend. Ik denk dat als dat verbeterd kan worden, dat weet ik niet hoor, dat dat dan voor de de taal dat wel goed gaat. Dat daar geen achterstanden bij komen, en dat ruzie maken, ik denk, als je goede kleine groepjes, en een goede begeleiding, en dat het allemaal vloeiend werkt, als die voorwaarden er zijn dat het dan wel goed moet gaan.

**○ Use: stress reduction**

**1 Quotations:**

**6:41 ¶ 131 in Interview 4, 19-2-2021--edited**

heel veel stress bij kinderen weg.

**○ Use: Workpressure**

**3 Quotations:**

**7:19 ¶ 85 in Interview 5 3-3-2021.mp4--edited**

er moet wel iemand zijn die dat aanstuurt. Het staat of valt op dit moment wel met mijn rol, en dat heeft alles te maken met de werkdruk,

**11:2 ¶ 55 in Interview 9 2021-04-07.mp4--edited**

: In het klaarzetten van de oefening. Het groepje kan er wel mee aan de slag, maar dat gaat best wel wat vooraf, in het programmeren. Kijk, zit het veel meer in je onderwijs verweven... Dan kun je ook zeggen: groep acht, die maakt iets voor groep één. Maar en dan is dat voor groep acht, voor de voor de meer intelligente groep die dat als uitdaging ziet, maar dat het zo ver is het gewoon niet. Maar ik zie de zeker echt goede kansen in. Ja.

**11:27 ¶ 53 in Interview 9 2021-04-07.mp4--edited**

Het enige nadeel is wel de intensiteit. Het is voor de de degene die de robot instelt en klaarzetten is het wel echt even intensief en daar daar zit nog wel een belemerende een factor
