# Supplementary material for: Do Robotic Tutors Compromise the Social-Emotional Development of Children?
Source: Front Robot AI. 2022 Jan 21;9:734955. doi: 10.3389/frobt.2022.734955 (PMC8814517; doi:10.3389/frobt.2022.734955)
Supplement: Supplementary file 2 [file DataSheet2.DOCX]

| ***Time and date:***  ***Permission to record the session:*** | |  |
| --- | --- | --- |
|  |  |  |
| **Question #** | **Topic** |  |
| 1 | **Socio demographic characteristics** | Years’ work experience as a teachers |
| 2 | **Socio demographic characteristics** | What kind of education (special or regular) |
| 3 | **Socio demographic characteristics** | Level of experience with social robots (1-5) |
| 4 | **Socio demographic characteristics** | Age |
| 5 | **Socio demographic characteristics** | Gender |
|  |  |  |
| 6 | **Use the social robot** | (How) did you use a social robot in your class? |
|  | *Follow up questions, more detail* | When did you use/see these robots? |
|  | *Follow up questions, more detail* | For how long were these robots used in a class? |
|  | *Follow up questions, more detail* | **How many children** interacted with the robot? |
|  | *Follow up questions, more detail* | **How many interactions** had a child (on average) with the robot? |
|  | *Follow up questions, more detail* | For wat **subject** were these robots used? |
|  | *Follow up questions, more detail* | Which **role** did the robot take on (more social or analytical (eg. STEM)). |
|  | *Follow up questions, more detail* | Did you **measure any effect** (e.g. skills development of learning gains) of the robot? |
| 7 | **Use the social robot** | Do you think robots can have a **place in primary education**, which place and why? |
|  | *Social development* |  |
| 8 | **Social development** | What kind of **influence** do social robots in education have on the **social development** of children, how and why? |
|  | *Follow up questions, more detail* | You could think of issues such as language development, learning, kind/unkind behavior, social interaction, friendship. |
| 9 | **Social development** | Have you **encountered this** (in your own) classes? |
|  | *Follow up questions, more detail* | What happened, **what did you see**/ notice? |
| 10 | **Social development** | How would you see/notice the impact of social robots on the social development of children? |
| 11 | **Social development** | Are **some children more sensitive** for a robot impacting their social development, why, what kind of children? |
| 12 | **Social development** | How can social robots be **best implemented,** to stimulate children’s social development? |
|  |  | How can social robots be best implemented, to ensure robots do not compromise children’s social development? |
|  | *Attachment* |  |
| 13 | **Become too attached** | Do you think children can **feel attached** to social robots? |
|  | **Become too attached** | Have you **encountered** this (in your own) classes? |
|  | *Follow up questions, more detail* | What happened, **what did you see/** notice? |
| 14 | **Become too attached** | **What would be considered too attached** to social robots? |
| 15 | **Become too attached** | Are there children would be **more sensitive for becoming attached** to social robots, why, what kind of children? |
| 16 | **Become too attached** | How can social robots be best implemented? |
|  | *Follow up questions, more detail* | To make use of the positive effects of bonding, and ensure children don't get too attached to robots? |
|  | *Finale questions* |  |
|  |  | Do you know teachers with experience in applying social robots, that also would like to be interviewed? |
|  | **End of interview** | Extra question, what did you hear from other teachers? What do you hear as most positives, and obstacles. |
